# Regulation of Gene Expression of Methionine Sulfoxide Reductases and Their New Putative Roles in Plants

**DOI:** 10.3390/ijms20061309

**Published:** 2019-03-15

**Authors:** Ewa M. Kalemba, Ewelina Stolarska

**Affiliations:** Institute of Dendrology, Polish Academy of Sciences, Parkowa 5, 62-035 Kórnik, Poland; ewelina.stolarska89@gmail.com

**Keywords:** gene expression, methionine sulfoxide, responsive element, transcription factor

## Abstract

Oxidation of methionine to methionine sulfoxide is a type of posttranslational modification reversed by methionine sulfoxide reductases (Msrs), which present an exceptionally high number of gene copies in plants. The side-form general antioxidant function-specific role of each Msr isoform has not been fully studied. Thirty homologous genes of Msr type A (MsrA) and type B (MsrB) that originate from the genomes of *Arabidopsis thaliana*, *Populus trichocarpa,* and *Oryza sativa* were analyzed in silico. From 109 to 201 transcription factors and responsive elements were predicted for each gene. Among the species, 220 and 190 common transcription factors and responsive elements were detected for the *MsrA* and *MsrB* isoforms, respectively. In a comparison of 14 *MsrA* and 16 *MsrB* genes, 424 transcription factors and responsive elements were reported in both types of genes, with almost ten times fewer unique elements. The transcription factors mainly comprised plant growth and development regulators, transcription factors important in stress responses with significant overrepresentation of the myeloblastosis viral oncogene homolog (MYB) and no apical meristem, Arabidopsis transcription activation factor and cup-shaped cotyledon (NAC) families and responsive elements sensitive to ethylene, jasmonate, sugar, and prolamine. Gene Ontology term-based functional classification revealed that cellular, metabolic, and developmental process terms and the response to stimulus term dominated in the biological process category. Available experimental transcriptomic and proteomic data, in combination with a set of predictions, gave coherent results validating this research. Thus, new manners *Msr* gene expression regulation, as well as new putative roles of Msrs, are proposed.

## 1. Introduction

The oxidation of methionine (Met) to methionine sulfoxide (MetSO) is inevitable for organisms living in an aerobic environment. The enzyme that catalyzes the reduction of MetSO to Met is methionine sulfoxide reductase (Msr). According to BRaunschweig ENzyme DAtabase (BRENDA), which is a comprehensive enzyme classification system, peptide-methionine (R)-S-oxide reductase (EC 1.8.4.12) is an enzyme that is classified as an oxidoreductase that acts on a sulfur group of donors, with disulfide as an acceptor. There are three known types of Msr: type A (MsrA), type B (MsrB), and type C, which is specific to eubacteria and unicellular eukaryotes [1,2,3]. Although MsrA is a reductase that is specific to the MetSO S epimer, and MsrB is specific to the MetSO R epimer, both reduce free and protein-bound MetSO [4]. More Msr studies are related to animals, although an extremely high number of *Msr* gene copies has been found in plants. For example, Arabidopsis and rice have fourteen and six *Msr* genes, respectively [5]. Studies have identified nine *MsrB* genes in Arabidopsis [6] and four *MsrB* genes in poplar [7]. Excluding review papers, over 200 experiments that are linked to human diseases and over 200 Msr studies in other animals can be found in the National Center for Biotechnology Information (NCBI) database. Approximately 60 experimental studies have been conducted on plants and algae. The most recent review demonstrated that nearly half of the studies were conducted on Arabidopsis, and only a few of the studies were conducted on rice and poplar [8]. Based on experimental data, the role of Msrs in redox homeostasis and signaling in plants was elucidated, providing many examples of the physiological functions of plant Msrs under oxidative, abiotic, and biotic stress conditions in different species. Most of the *MsrA* and *MsrB* genes encode proteins, with masses of approximately 25 kDa and 15 kDa, respectively. There is little or no sequence homology between the MsrA and MsrB proteins [9]. Within each MsrA and MsrB plant family, the sequence identity among isoforms ranges between 1% and 15% [7]. The increased production of reactive oxygen species and the subsequent oxidative modification of proteins accompanies important physiological transitions in plants. MetSO studies in Arabidopsis under oxidative stress led to the identification of approximately 400 proteins containing this reversible posttranslational modification that are potential targets of Msrs [10]. Thus, the multiplicity of plant Msr isoforms allowed for us to suppose that the Msr system might be involved in the regulation of many aspects of plant life.

Experimental studies have revealed several factors, mainly stress factors, regulating the expression of *Msr* genes in Arabidopsis. Few experiments have been performed on poplar and rice; thus, less information is known regarding the regulation of Msr expression in these plants than in other species. In previous studies, oxidative stress affected the expression of *MsrB* Arabidopsis genes [11] and the synthesis of AtMSRA2 [12], AtMSRA4 [12,13], AtMsrB7 [14], AtMSRB9 [15], and OsMSRB5 proteins. High light intensity modified the level of AtMSRA4 [12,13]. UV treatment induced the expression of *MSRB7* and *MSRB8* genes [16]. Low temperatures impacted the levels of AtMSRB3 [17], AtMSRB1 [18], and AtMSRB2 plastid proteins [12,18], and *OsMSRA4.1* and *OsMSRB1.1* transcripts [19]. High temperature induced and repressed the AtMSRA4 and AtMSRA2 protein levels, respectively [18], and modified *OsMSRA4.1* and *OsMSRB1.1* gene expression [19]. High salinity modified the levels of AtMSRA4 [12] and all *OsMSR* transcripts [20], particularly *OsMSRA4.1* and *OsMSRB1.1* [19]. Dehydration stress enhanced the synthesis of the AtMSRB9 protein [15]. Heavy metal stress increased the accumulation of the mRNA of all AtMsrs [21] and the OsMSRB5 protein [22]. Pathogen-induced changes in Arabidopsis *MSRB7* and *MSRB8* expression [16,23] and the poplar pMsrA and MsrB2 protein levels [18] have also been reported. Treatment with NO upregulated the AtMsrB7 [24] and *OsMSR* transcripts [20]. Mannitol, methyl viologen, and abscisic acid (ABA) treatments induced *OsMSRA4.1* and *OsMSRB1.1* transcription [19]. Combined ABA and NaCl treatment increased the AtMSRA4 levels [12]. Interestingly, AtMsrB7 was found to be responsive to linolenic acid, which is the precursor of jasmonic acid (JA) [25]. PMSR2 partially regulates *A. thaliana* development [26]; however, no data are available regarding the involvement of specific Msrs in the regulation of basic biological processes, leading to plant growth and development. Microarray data analyses have revealed that the expression levels of *Msrs* are organ-specific. The highest abundance of the AtMSRA4 transcript was detected in fully developed leaves, while AtMSRA2 and AtMSRA3 were dominant in the roots, and stamen and pollen contained mostly AtMSRA3 transcripts [7,11]. For the B-type genes, AtMSRB1, AtMSRB2, and AtMSRB6 mRNAs have been reported to be abundant in leaves, whereas AtMSRB5, AtMSRB7, AtMSRB8, and AtMSRB9 have been reported to be abundant in roots [7,11,13]. In rice, *OsMSRA4.1* was constitutively expressed in all tissues, whereas *OsMSRB1.1* was more abundant in leaves, flowers, and calli than in the roots and stems.

The above experimental results certainly do not present all the possibilities of *Msrs* gene expression regulation. To determine which compounds and factors may affect *Msrs* gene expression, in silico predictions that are based on the servers and programs listed in the bioinformatics resources portal, which was shown to be statistically reliable by previous publications, may provide interesting hints for further experimental studies. The region of DNA that was located upstream of a particular gene, called a promoter, mainly regulates gene expression by proteins that are known as transcription factors (TFs), which recognize specific DNA sequences within the promoter region and specific activators binding to responsive elements (REs). A genome-wide search for putative promoters revealed that Arabidopsis, poplar, and rice contain 17,896, 17,645, and 22,258 genes, respectively, with a transcription start site (TSS) ≥ 1, comprising over 99% of the genes in each genome [27]; these results emphasize the complexity of gene expression regulation. Here, we predominantly focus on the above three model species, because their *Msr* genes are homological, and the promoter regions of their *Msr* isoforms are well defined. TSS selection depends on the cellular and tissue context, developmental and physiological stage, environmental conditions, and the set of accessible TFs [27]. For a single gene, the proximal promoter (200–300 bp upstream of the TSS), together with the distal promoter, might contain hundreds of consensus sequences for different TFs [28]. Thus, the protein-DNA complex can upregulate or downregulate gene expression. TFs recognize primary, secondary, or even additional motifs that differ from each other, emphasizing the importance of TFs in the complex regulation of gene expression [29]. TFs representing the following families have been identified in plants: v-myb avian myeloblastosis viral oncogene homolog (MYB), basic Helix-Loop-Helix (bHLH), tryptophan, arginine, lysine and tyrosine domain (WRKY), no apical meristem, Arabidopsis transcription activation factor and cup-shaped cotyledon (NAC), and basic leucine zipper (bZIP). The multifunctionality of these TFs in the regulation of biological processes, including plant growth and development (MYB, NAC, bZIP, bHLH) and various stress responses (MYB, NAC, bZIP, bHLH, WRKY), has been extensively reviewed [30]. In particular, the participation of TFs from MYB and NAC families in the regulation of plant growth and development and stress responses has been well documented [31,32]. MYB is among the largest families of plant TFs that control various plant processes, such as development, differentiation, metabolism, defense, and stress responses. R2R3-MYB is a plant-specific class of MYB groups that contains over one hundred members in the genomes of different plant species [31]. NACs belong to a plant-specific family of TFs that is involved in various plant stress responses [32]. The extensive action of NAC TFs is derived from the large number of *NAC* genes in genomes. For example, there are 117 *NAC* genes in Arabidopsis, 163 *NAC* genes in poplar, and 151 *NAC* genes in rice [32]. The expression of ANAC2, NAC001, NAC004, NAC005, NAC013, and NAC019 was shown to be strictly connected to various stress conditions ([32] and references therein). In particular, ANAC013 functions in the regulation of the oxidative stress response [33], and NAC103 [34] and NAC87 modulate ROS [35]. The MYB-NAC regulatory network assures many developmental transitions in plants. For example, the interaction of MYB108 with the *ANAC003* promoter region controls leaf senescence in Arabidopsis [36]. The cooperation of MYB and NAC TFs was expected, since both of the family members have been experimentally shown to be involved in osmotic stress and oxidative stress responses.

To verify whether different types and isoforms of Msrs are required for a range of distinct physiological states and responses to different stress conditions, the promoter regions of 30 homological *Msr* genes that represent 14 A-type isoforms and 16 B-type isoforms were analyzed in a search for TF binding sites, the specific TFs recognizing these sites and REs. The pool of predicted TFs and REs was investigated to determine whether a group of species-specific, type-specific, or isoform-specific TFs and REs could be distinguished. Based on the PLANTCARE database, 23 cis elements were predicted in the promoters of all Arabidopsis A-type *Msr* genes [13]. While suspecting that *Msr* promoters contain more REs, we performed a search using MatInspector software [37] that was based on the Plant cis-acting regulatory DNA elements (PLACE) database [38] because the MatInspector library contains 634 matrices, representing the largest library that is available for TF binding site searches.

## 2. Results

### 2.1. Prediction of Transcription Factor Binding Sites

Known and homologous *MsrA* and *MsrB* genes that originate from the genomes of Arabidopsis (*Arabidopsis thaliana*), poplar (*Populus trichocarpa*), and rice (*Oryza sativa*) were analyzed. The promoter region sequence of each gene was analyzed with a search of the REs and binding sites for plant-specific TFs to establish the potential physiological and environmental factors that regulate *Msr* gene expression. In total, 30 gene sequences were analyzed, and the number, length, and location of promoter regions and the number of coding transcripts were specified, as in Table S1. Two or more coding transcripts were established for 18 *Msr* genes, Table S1A–C. *AtMsrB5* was assigned the highest number of coding transcripts (five). Over two hundred TF binding sites were predicted for each gene, as in Table 1. The largest numbers of TF binding sites and REs were predicted for *MsrB9* originating from Arabidopsis, *MsrB3.2* originating from poplar, and *MsrA5* originating from rice. As the consensus core sequences were repeated within each promoter region, the final number of specific nonredundant TFs and REs ranged from 109–201 per gene.

**Figure 1:**
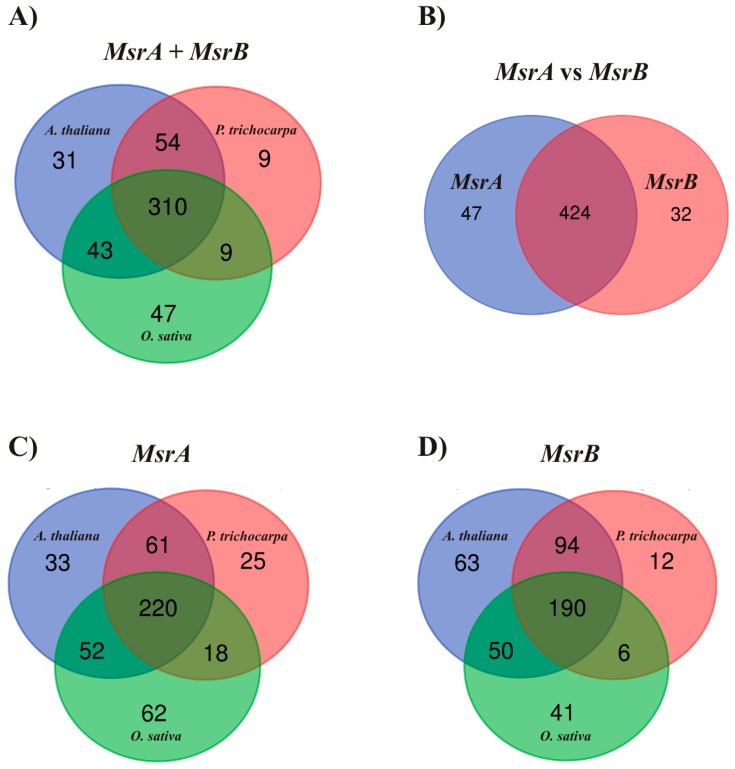
Venn diagrams comparing the transcription factor binding sites and responsive elements predicted for 30 homological genes of methionine sulfoxide reductase type A (*MsrA*) and B (*MsrB*) originating from the *Arabidopsis thaliana*, *Populus trichocarpa*, and *Oryza sativa* genomes. The three-set Venn diagrams show species-dependent (**A**), species-dependent and A-type-dependent (**C**), and species-dependent and B-type-dependent (**D**) interactions. The two-set Venn diagram shows type-dependent intersections (**B**), irrespective of species.

The TFs and REs also repeated among *Msr* isoforms and types; thus, in total, approximately 400 potential TFs and REs were predicted. A total of 310 TFs and REs overlapped among all 30 *Msrs*, as in Figure 1. A total of 220 common TFs and REs were detected for A-type *Msrs* and 190 common TFs and REs were detected for B-type *Msrs*, as in Figure 1. Arabidopsis and poplar shared the largest number of identical TFs and REs, Figure 1. Slightly fewer TFs and REs were shared between Arabidopsis and rice, while poplar and rice had the least common elements. Poplar was characterized by the lowest number of TFs and REs unique to the *Msrs* of this species. When comparing the 14 *MsrAs* and 16 *MsrBs,* a total of 424 TFs and REs were reported in both types of *Msrs*, and almost ten times fewer TFs and REs were unique to each type. The intersections were also calculated between each gene of the group of 30 TFs and REs that can potentially regulate most of the analyzed *Msrs*, as well as TFs and REs that are unique to each isoform of each type in each species, as in Table S2.

### 2.2. The Common TFs and REs

Among all the identified TFs, 73 different MYB-type TFs, 39 different NAC-type TFs, 26 zinc finger TFs, 21 homeobox domain TFs, and 16 WRKY family TFs were identified. In general, the members of the MYB and NAC families were overrepresented. Thirty-five specific MYB TFs, Table S2C, and 29 NAC-containing domain proteins were predicted to bind to the *Msr* promoter regions of all tested species, as in Table S2C. Beyond the known TFs, several response elements and motifs are recognized as activated by plant hormones (JA, abscisic acid (ABA), gibberelins, ethylene), sugar, prolamines, nitrogen, iron, microRNAs, and environmental factors, such as light, heat, and cold. The majority of genes were reported to contain a core consensus sequence for binding TFs assigned to the regulation of different growth and physiological stages, such as plant morphogenesis, as well as a group of TFs that are functionally assigned as sensitive to environmental factors, such as light, heat, drought, osmotic stress, oxidative stress, and multiple abiotic stresses, regulating plant defenses and responses to biotic and abiotic stress. The two above groups are linked because of the detection of numerous TFs acting in response to auxin, salicylic acid, JA, ethylene, auxin, ABA, and cytokinins, which have important signaling roles in both plant growth and development, as well as in response to stress conditions.

A list of TFs that are predicted to bind the promoter region in over 75% of the analyzed genes was created, as shown in Table 2. The vast majority of TFs, including homeodomain proteins (WUSCHEL and GLABROUS1), homeobox proteins (HB32 and HB34), two repressors (BELLRINGER and AS1/AS2), MYB proteins (DIVARICATA 1, ODO1), members of the KANADI class, TESMIN/TSO1-like CXC 2 (TCX2), stomatal carpenter 1 (DOF5.7), G2-like family protein (GLK), SBF-1, protodermal factor 2 (PDF2), Yabby transcription factor CRABS CLAW, and late elongated hypocotyl I (LHY), were associated with the regulation of growth and development. Other TFs, including MYB96, NAC proteins (GmNAC30, GmNAC81), class I GATA factors, trihelix transcription factor GT-1, storekeeper (STK), and bZIP protein G-Box binding factor 1 (GBF1), were involved in the stress responses. Development-related and stress-related groups of TFs included two ethylene-responsive factors (RAP2.2 and RAP2.7) and jasmonate-responsive elements. Apart from the typical TFs, the majority of the analyzed *Msrs* also contained nodulin consensus sequences, GAAA motifs that are involved in pollen-specific transcriptional activation, motifs of different sugar-responsive genes, and prolamin boxes conserved in cereal seed storage protein gene promoters.

### 2.3. Type-Specific and Species-Specific TFs and REs

Forty-seven TFs and REs that were specific to *MsrA* and 32 TFs and REs specific to *MsrB* were recognized, Table S2A. Both types of *Msr* contained representatives of the main plant TF families; however, 12 TFs and REs responsive to ethylene were uniquely found in the A-type group. The list of species-specific TFs and REs is given in Table S2B. Species-specific TFs and Res that are predicted for rice included 17 ethylene-responsive TFs, five TCP TFs, three dehydration-responsive elements, and no MYB or DOF family members. Species-specific TFs and REs that were predicted for Arabidopsis included nine NAC TFs and three TFs in the WRKY family. Only nine species-specific TFs were predicted for poplar; these TFs included three zinc finger proteins in the DOF family, two MYB TFs, and no TFs from the NAC and WRKY families. Both type-specific and species-specific groups of TFs and REs were relatively numerous and varied; thus, we searched for more pronounced differences at the isoform-specific level.

### 2.4. Isoform-Specific TFs and REs

By developing the intersections of all 30 genes (Table S2C), unique TFs and REs were found for each gene, as in Table 3. It was predicted that specific members of MYB, NAC, DOF, and WRKY families that are different from these listed in Table 2 might regulate the Msr genes. Interestingly, four different ethylene-responsive TFs were found to interact singularly with *OsMsrA5*. *AtPMSR3* was predicted to be cooperatively regulated by two hormones ethylene and JA. The expression of *OsMsrA2.2* was reported to be iron-dependent. *OsMsrA4* was described as containing unique redox-responsive elements.

### 2.5. GO Classification

**Figure 2:**
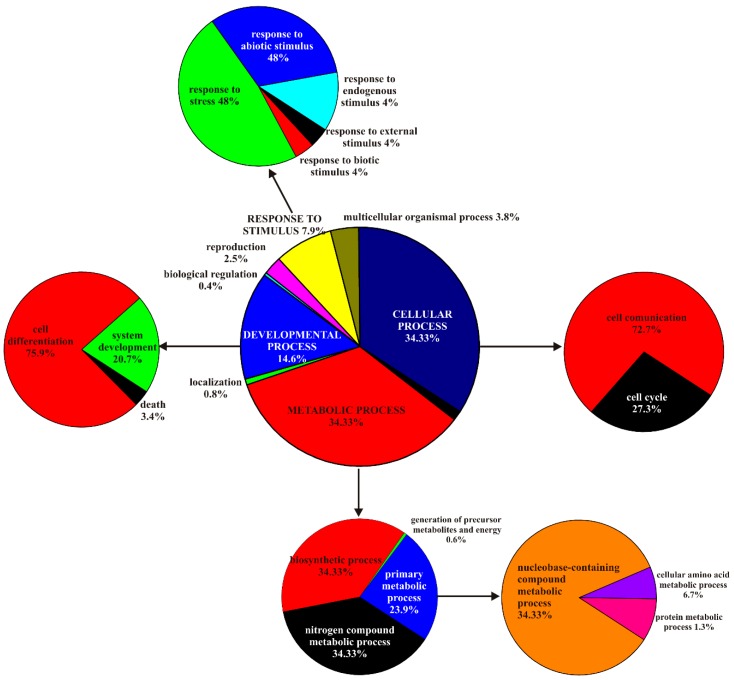
PANTHER biological process analysis based on gene ontology terms. Functional classification of genes encoding TFs predicted to interact with the promoter regions of *MsrA* and *MsrB* originating from the *Arabidopsis thaliana*, *Populus trichocarpa*, and *Oryza sativa* genomes.

To establish the participation of all detected TFs in specific biological processes, the list of gene IDs of transcription factors was functionally analyzed based on Gene Ontology (GO) terms using the protein annotation through an evolutionary relationship (PANTHER) classification system [39]. Among these TFs, 347 genes were annotated with GOs and 251 hits were represented in the biological process category, as in Figure 2. Two categories were equally overrepresented: cellular process and metabolic process. The term developmental processes comprised 14.6% of the analyzed genes, and the term response to stimulus comprised 7.9% of the analyzed genes. The four main categories were analyzed in greater detail. The cellular process category contained members of cell communication and cell cycle, which represented 72.7% and 27.3% of all hits, respectively. Within the metabolic process category, two terms, biosynthetic process and nitrogen compound metabolic process, were equally (37.8%) overrepresented. The primary metabolic process term constituted 23.9% of the hits and it represented the nucleobase-containing compound metabolic process. The developmental process terms were mainly cell differentiation and system development. Nearly half of the terms in the response to stimulus category were composed of the response to stress members. The response to abiotic, biotic, endogenous, and external stimuli terms complemented this category.

## 3. Discussion

The possible presence of up to five coding transcripts and up to 201 putative TFs and REs in a single *Msr* gene makes the regulation of *Msr* gene expression in plants highly complex. Irrespective of species-dependent or Msr type-dependent analyses, we demonstrated that up to ten times more common than unique TFs and REs might bind to the *Msr* promoter regions. Thus, the TFs and REs comprising the group of common elements appear to contain the most likely candidates regulating the expression of *Msr* genes.

### 3.1. Putative Involvement of Msrs in Regulation of Plant Growth and Development

Among the TFs and REs that were predicted to bind the majority of analyzed *Msr* genes, the bulk were assigned to the regulation of plant growth and development processes, suggesting the accompanying expression of *Msrs* during these processes. The majority of the experimentally proven functions of particular TFs comes from Arabidopsis studies. In summary, 11 of the TFs (PDF2, SBF, WUSCHEL, TESMIN/TSO1-like CXC, KANADI1, GLK, DOF5.7, LHY, repressor AS1/AS2, GBF1, STK) that were predicted to bind the promoter regions of most of the analyzed *Msrs* act in plant growth and regulation. Studies have demonstrated that the above TFs are involved in shoot development [40], lateral root formation and development [41], embryo development [40], leaf development [42,43], stomata patterning [44], chloroplast development [45], the development of reproductive tissues [46], early embryogenesis [47], seedling development [48], senescence [49], the regulation of circadian rhythms [50], and cell-cycle-dependent transcription-enabling plant growth [47]. Interestingly, seven of the TFs that are listed in Table 2 (WUSCHEL, DIVARICITA, BELLRINGER, HB32 and HB34, RAP2.7, CRABS CLAW, ODO1) control the flowering process by regulating floral development [51,52], fragrance [53], and nectary development [54] and the initiation and repression of flowering [47,49,55,56]. In this context, it can be hypothesized that Msrs are activated by specific TFs and they mediate this important developmental transition in plants. This hypothesis is supported by the fact that the biological process analysis that is based on GO terms confirmed that the predicted TFs are involved in developmental processes that include cell differentiation and the system development subcategory, which consists of plant organ development, root system development, and shoot system development child terms in the GO classification system; furthermore, this hypothesis is supported by literature data that show that PMSR2 partially regulates Arabidopsis development [26].

### 3.2. Predictions vs. Experimental Data

#### 3.2.1. The Involvement of MYB and NAC Family TFs in the Regulation of *Msr* Gene Expression

Both EFM [57] and MYB12 [58] mediate plant responses to light stimulus, and they both were found to bind promoters of MsrA in Arabidopsis. It is possible that the two MYB TFs are involved in changes in PMSR activity detected during light and dark periods [26]. Tissue-specific gene expression is regulated by TFs [59]. Similarly, MYB factors might mediate the organ-specific expression of Msrs. MYB77 was predicted to bind to the *MsrA2*, *MsrB4,* and *MsrB9* promoters, whereas MYB93 was predicted to bind to the *MsrB8* promoter. Interestingly, MYB77 [60] and MYB93 [61] are specific to lateral root growth and development, and the expression of *MsrA2*, *MsrB4*, *MsrB9*, and *MsrB8* was demonstrated to be specific to roots [7,11,13].

The members of the MYB and NAC families can be mediators in both the increased synthesis of AtMSRB9 proteins during dehydration stress [15] and the upregulation of PMSRA4 under oxidative stress conditions [13], because the *PMSR4* promoter contains 34 binding sites for MYB-type TFs and 17 binding sites for NAC-type TFs. Similarly, the promoter of *AtMsrB9* contains 42 binding sites for MYB-type TFs, including binding sites for MYB49, MYB52, and MYB96, which act under water stress [62,63,64], and 35 binding sites for NAC-type TFs, including dehydration-related NAC096 [65]. The NAC-type TFs IDEF1/IDEF2, which are similar to MYB58 [66], are linked to iron deficiency in plants [67]. Therefore, Msrs might be susceptible players in the regulation of redox-sensitive elements. This speculation is likely true, because OsMSRB5 was shown in rice seedlings to be involved in defenses against copper toxicity [22], and cadmium upregulated the entire Msr system in Arabidopsis [21]; in addition, MYB62 plays a role in the regulation of phosphate starvation responses [68].

#### 3.2.2. Hormone-Dependent Regulation of *Msr* Gene Expression

Experimental data showed that *Msr* expression in Arabidopsis is ABA- [12] and JA-dependent [25]. JA signaling is important in the responses to biotic and abiotic stresses and in plant development [69]. The JA response element was in 29 promoters of the 30 analyzed *Msr* genes. The exception with no JA response element was *OsMsrA2.2*, which had a promoter containing elements that were uniquely responsive to ABA and ethylene. *AtPMSR3* seems to be especially regulated by JA, because *AtPMSR3* was the only Msr gene containing the responsive element that is cooperatively regulated by ethylene and jasmonate. The prediction of abscisic acid-responsive element-binding factor 2 in promoters of *OsMsrA4*, *OsMsrB1,* and *AtPMSR4* is consistent with experimental data showing the ABA-induced expression of OsMsrA4.1, OsMsrB1.1 [19], and AtMSRA4 [12]. Moreover, salt stress and NO in an ABA-dependent manner increased the expression of OsMSRA4, OsMSRA5, OsMSRB1.1, OsMSRB3, and OsMSRB5, whereas OsMSRA2.1 was modulated independent of ABA [20]. MYB44, which was demonstrated to confer ABA-dependent resistance to abiotic stresses [70], was predicted to interact with the *MsrB5* homolog in poplar and rice. The effect of ethylene on *Msr* expression has not been experimentally tested in Arabidopsis, poplar, or rice. Our analyses revealed that the ethylene-responsive elements are abundant and characteristic of A-type Msrs. Ethylene strongly induced tomato SlMSRA1 [71], indicating that other isoforms of *Msrs* might also be upregulated through ethylene-mediated responses. In particular, four additional ethylene-responsive factors that were unique to the *OsMsrA5* isoform were found. Therefore, the ethylene-dependent regulation of *Msr* expression is worth consideration. TFs classified as the GA-regulated myb gene from barley were identified in 21 promoters of the 30 analyzed genes, and GA-inducible regulatory elements were identified in 11 promoters, including five isoforms different from the group of 21. There are no experimental data on GA-dependent *Msr* expression in the literature; however, cross-talk between hormones is well known. For example, MYB96, which is the TF interacting with the majority of the promoters of analyzed *Msr* genes, mediates the cross-talk between ABA and JA [62]. Cis-regulatory elements that are involved in salicylic acid induction of secretion-related genes via NPR1 were detected in 17 different *Msr* genes, including types A2-A5. In tomato, SlMsrA2, SlMsrA3, and SlMsrA5 transcripts increased upon salicylic acid treatment, whereas SlMsrA4 transcripts decreased [71], underlying the fact that the expression of *Msr* genes is hormone-sensitive. The DOF family is crucial in response to salicylic acid [72]. DOF5.7 commonly regulates *Msrs*. Moreover, *AtMsrB7* is the only *Msr*-containing DOF4.7 binding site, and DOF5.1 and DOF2.2 can singularly bind to the *PtMsrA2.1* promoter, while DOF5.8 can bind to the *PtMsrB3*.1 promoter. Auxin response elements were recognized in the promoters of 18 analyzed *Msr* genes. Auxins might indirectly affect *Msr* expression, possibly through reactions that are linked to MYB TFs, as MYB12 [58], MYB77 [60], and MYB88 [73] modulate the response to auxin, and therefore growth and developmental processes.

#### 3.2.3. Seed Traits Control

Among the Arabidopsis Msrs, two genes, i.e., *AtMsrB2* and *AtMsrB6*, have been identified as organ-specific genes in seeds [7]. The promoter regions of both genes contained binding sites for WUSCHEL, NAC2, and GLABBOROUS TFs, which were demonstrated to be involved in seed morphogenesis [74,75] and seedling development [48], respectively. The *AtMsrB6* promoter also contained binding sites that are specific to MYB56, which regulates seed size [76], and MYB107, which affects seed coat suberin assembly [77]. The *AtMsrB2* promoter included binding sites for MYB33 and MYB65, which promote protein storage reserve utilization during seed germination [78], and it uniquely contained a binding site for NAC081, which is required for normal seed development and morphology [75]. The above information again supports the compatibility between the in silico predictions and experimental evidence. We speculate that, based on the large group of predicted TFs, the Msr system is also important for other seed-associated traits, such as desiccation tolerance, seed development and dormancy, germination, and responses to oxidative stress. This speculation might be supported by experimental results in which the accumulation of MsrB was related to desiccation tolerance in seeds [79] and the fact that MSRB5 was upregulated after oxidative stress in response to the sulfur nutrition of Arabidopsis seeds [80]. A-type Msr expression was also reported during the morphogenesis and maturation of *Acer platanoides* seeds [81]. Interestingly, FUSCA3, which can bind to the *AtPMSR4* promoter, acts as a major regulator of seed maturation in Arabidopsis [82]. Thus, MsrA4 might impact seed development and seed aging by regulating redox homeostasis, because the rice *MsrA4* promoter can only interact with redox-responsive transcription factor 1. Experimental data confirmed that the Msr system is involved in seed longevity [83].

### 3.3. Perspectives

The sugar-dependent regulation of *Msr* expression definitely requires investigation in subsequent studies, because no data on this subject are available in the literature. The predicted involvement of Msrs in the regulation of plant growth and development, particularly during flowering and seed production, requires experimental confirmation. In the future, relatively simple experimental research will be required to understand the effects of plant hormones on Msr expression at the mRNA and protein levels. Among all of the predicted TFs, MYBs and NACs seem to be the most influential factors in the regulation of *Msr* gene expression, and some speculations regarding the perspectives of possible Msr functions can be proposed. It was experimentally proven that MYBs are involved in the regulation of many biological processes and stress responses, such as secondary wall biosynthesis [84] and thickening [85], stamen maturation [86], programmed cell death [78], microsporogenesis [87], wounding [88], trichome formation [89], and xylem development [90], which have not yet been linked to the corresponding *Msr* genes and proteins; these processes and responses are worth further attention. Our meta-analysis was definitely limited by the fact that not all of the plant genomes are sequenced, and the isoforms of *Msrs* genes are not characterized fully enough to study their promoter regions. However, this study provides a useful reference for researchers who are interested in the Msr system. The selection of homologous genes was planned to avoid species-specific TFs and REs. Data in the literature shows that, among the hundreds of TFs and Rest that are currently identified, 15 TFs and four REs found in the promoter region of a specific *Msr* gene can affect its expression in a manner that is specific to the TF or RE. The fact that our predictions coincided with experimental data makes our results more reliable. However, further experimental evidence is needed to confirm that our findings are linked to the regulation of *Msr* gene expression and the new putative roles of Msrs in plants.

## 4. Materials and Methods

### 4.1. Material

A total of 30 *MsrA* and *MsrB* gene sequences were analyzed. Five isoforms of *MsrA* (*PMSR1*, *PMSR2*, *PMSR3*, *PMSR4*, *PMsrA5*) and nine isoforms of *MsrB* (*MsrB1*, *PMsrB2*, *MsrB3*, *MsrB4*, *MsrB5*, *MsrB6*, *MsrB7*, *MsrB8*, *MsrB9*) genes originating from the *Arabidopsis thaliana* genome were analyzed. Gene sequences were derived from the Arabidopsis genome using the TAIR 10 database (https://www.arabidopsis.org/). Table S1A provides additional information, including Arabidopsis *Msr* descriptions. Five isoforms of *MsrA* (*MsrA2.1*, *MsrA2.2*, *MsrA4.1*, *MsrA4.2*, *MsrA5*) and four isoforms of *MsrB* (*MsrB1*, *MsrB3.1*, *MsrB3.2*, *MsrB5*) genes originating from the *Populus trichocarpa* genome were analyzed. Gene sequences were derived from the poplar genome using the Poptr 2.0 database (https://phytozome.jgi.doe.gov/pz/portal.html#info?alias=Org_Ptrichocarpa). Table S1B provides additional information, including poplar *Msr* descriptions. Four isoforms of *MsrA* (*MsrA2.1*, *MsrA2.2*, *MsrA4*, *MsrA5*) and three isoforms of *MsrB* (*MsrBl*, *MsrB3*, *MsrB5*) genes originating from the *Oryza sativa* genome were analyzed. Gene sequences were derived from the rice genome while using the MSU release 7 database (http://www.plantedb.org/OsGDB/).

### 4.2. The Search for Transcription Factor Binding Sites and Responsive Elements

The search for TF binding sites and REs was performed for 30 *Msr* genes (Table S1) while using Matlnspector software [40]. For each gene, all of the functional promoters were extracted directly from the ElDorado genome database (https://www.genomatix.de/online_help/help_eldorado/introduction.html). The location and length of each promoter were accompanied by the number of coding transcripts, and their accession numbers were specified; these numbers are given for each analyzed gene in Table S1. The promoter sequences were searched for TFBS using a comparison of gene identification numbers (IDs) against predefined IUPAC library plant TF sites based on the Plant cis-acting regulatory DNA elements (PLACE) database [41]. The Matlnspector library represents the largest library available for TF binding site searches.

### 4.3. GO-Based Functional Classification

The PANTHER classification system (http://www.pantherdb.org/) was used to assess the involvement of the genes of the predicted TFs annotated with GO terms in the biological process and molecular function categories [42]. The gene list analysis was performed using a list of gene identification numbers (ID) corresponding to the predicted TFs, Table S3. All of the IDs were derived from the NCBI gene database (https://www.ncbi.nlm.nih.gov/), specifically from the Arabidopsis genome, which was queried by the search terms.

## 5. Conclusions

Overall, the antioxidant role and the role in stress-related responses of different Msr types and isoforms have been experimentally proven, and therefore have high confidence. Here, new putative roles and regulatory mechanisms were predicted for *Msrs* in silico. Based on our research, we suggest that Msrs are involved in the regulation of many plant growth and development processes, including flowering. Among the studied phytohormones, *Msrs* are very sensitive to JA and ethylene, and ethylene principally regulates the A-type *Msrs*. *Msrs* are also under the control of gibberellins and auxins. Therefore, Msrs might mediate important hormone-dependent developmental transitions in plants and stress-related responses. Mainly MYB and NAC TFs control *Msr* expression. In particular, MYBs might determine the organ-specific expression of Msrs. Presumably, the Msr system is also significant for seed-associated traits. The predicted and described TFs might individually bind to *Msr* promoters, while considering the cellular and tissue context, developmental and physiological stage, environmental conditions, and pool of accessible TFs required for the selection of a particular TSS. Thus, the Msr system might participate in important biological processes and stress responses in plants, exhibiting a protective role.

## Figures and Tables

**Table 1:** Total number of transcription factor binding sites (TFBS) and responsive elements (REs), followed by the number of redundant transcription factors (TFs) and REs predicted for 30 homological genes of methionine sulfoxide reductase type A (*MsrA*) and B (*MsrB*) originating from the *Arabidopsis thaliana* (At), *Populus trichocarpa* (Pt), and *Oryza sativa* (Os) genomes.

Gene Name	TFBS + REs	TFs + REs
*AtPMSR1*	249	163
*AtPMSR2*	325	186
*AtPMSR3*	310	174
*AtPMSR4*	249	158
*AtMsrA5*	254	162
*AtMsrB1*	267	157
*AtMsrB2*	294	169
*AtMsrB3*	255	155
*AtMsrB4*	210	144
*AtMsrB5*	286	174
*AtMsrB6*	362	170
*AtMsrB7*	298	175
*AtMsrB8*	283	147
*AtMsrB9*	368	178
total	**4010**	**438**
*PtMsrA2.1*	327	168
*PtMsrA2.2*	298	130
*PtMsrA4.1*	320	149
*PtMsrA4.2*	252	152
*PtMsrA5*	285	154
*PtMsrB1*	258	160
*PtMsrB3.1*	289	150
*PtMsrB3.2*	366	144
*PtMsrB5*	241	145
total	**2636**	**382**
*OsMsrA2.1*	272	170
*OsMsrA2.2*	266	109
*OsMsrA4*	277	172
*OsMsrA5*	464	201
*OsMsrB1*	208	146
*OsMsrB3*	248	141
*OsMsrB5*	223	138
total	**1958**	**409**

**Table 2:** List of TFs predicted to interact with the promoter region of at least 75% of 30 homological genes of methionine sulfoxide reductase type A (*MsrA*) and B (*MsrB*) originating from the *Arabidopsis thaliana* (At), *Populus trichocarpa* (Pt), and *Oryza sativa* (Os) genomes. The core consensus sequence of each TF is underlined.

TF	Consensus Sequence	Not Detected in the Promoter of
Protodermal factor 2	aaattgcaaATTCtagg	*OsMsrA2.2*
SBF-1	ggatttagttTTAAaattt	*OsMsrA2.2*, *OsMsrA5*, *OsMsrB3*
Homeodomain protein WUSCHEL	gacgccATTAacacgtggc	*OsMsrA2.2*, *PtMsrA2.2*, *PtMsrB5*
Homeodomain GLABROUS 1	cacgtgttAATGgcgtc	*AtPMSR1*, *OsMsrA2.2*, *OsMsrB1*
TESMIN/TSO1-like CXC 2	atcaatctctcattCAAAatctcattctctc	*AtMsrB4*, *OsMsrA2.2*, *OsMsrA5*, *PtMsrA4*
KANADI	tcGAATaacaaat	*AtMsrB3*, *OsMsrA2.2*, *OsMsrB3*, *OsMsrB5*, *PtMsrB3.1*
G2-like family protein	caatagATTCctt	*OsMsrA2.2*, *OsMsrB1*, *OsMsrB3*, *OsMsrB5*, *PtMsrA4.2*
Stomatal Carpenter 1 (DOF5.7)	ttAGTTaacca	*AtMsrB7*, *OsMsrA4*, *OsMsrB3*, *PtMsrA2.2*, *PtMsrA4.2*
DIVARICATA 1	tgctcgtTATCttcagccc	*AtMsrB6*, *OsMsrA2.1*, *OsMsrA2.2*, *OsMsrA5*, *OsMsrB1*
Trihelix transcription factor GT-1	gaacatttgGTTAactaaa	*AtMsrB4*, *OsMsrA2.2*, *OsMsrA5*, *OsMsrB3*, *OsMsrB5*
Transcriptional repressor BELLRINGER	tcacaaaATTAattcttct	*AtMsrB2*, *AtMsrB5*, *AtPMSR4*, *OsMsrA2.2*, *OsMsrB1*
RAP2.2	atATCTaacaa	*AtMsrB1*, *AtMsrB8*, *OsMsrA2.2*, *OsMsrA5*, *OsMsrB1*, *OsMsrB3*
DNA-binding storekeeper protein-related transcriptional regulator (AT4G00250)	aaaattGATCcaaagct	*AtMsrB2*, *AtMsrB6*, *AtMsrB7*, *AtPMSR3*, *OsMsrA2.2*, *PtMsrA4.1*
Class I GATA factors	aagatGATAaatgtgtg	*AtMsrB6*, *AtPMSR2*, *OsMsrA2.2*, *OsMsrA5*, *OsMsrB1*, *PtMsrB5*
Homeobox protein 32	tagatttaTTATttgtatc	*AtPMSR1*, *OsMsrA2.2*, *OsMsrA4*, *OsMsrB3*, *OsMsrB5*, *PtMsrB1*, *PtMsrB5*
Myb-like protein of *Petunia hybrid* (ODO1)	ttaggattTAGTtttaaaatt	*AtMsrB3*, *AtMsrB4*, *OsMsrA2.1*, *OsMsrA4*, *OsMsrA5*, *PtMsrA2.2*, *PtMsrB3.2*
Heterodimer of NAC-domain transcription factors GmNAC30 and GmNAC81	TGTGttg	*AtMsrB2*, *OsMsrA2.1*, OsMsrA2.2, *OsMsrB1*, *OsMsrB5*, *PtMsrA4.2*, *PtMsrB5*
Myb domain protein 96 MYBCOV1	ttcgtatttAGTTaaccaaat	*AtMsrB3*, *AtMsrB4*, *OsMsrA2.2*, *OsMsrA4*, *OsMsrA5*, *PtMsrB3.2*, *PtMsrB5*
Late elongated hypocotyl 1	tgtgtaTATAttttgga	*AtMsrA5*, *AtMsrB1*, *AtMsrB4*, *OsMsrA2.2*, *OsMsrA5*, *OsMsrB3*, *PtMsrB5*
Target of early activation tagged 1 (RAP2.7)	tgacATTAaaa	*AtMsrB4*, *AtMsrB6*, *AtMsrB8*, *AtPMSR4*, *OsMsrA2.1*, *OsMsrA2.2*, *OsMsrB1*
Yabby transcription factor CRABS CLAW	aagaTGATaaatg	*AtPMSR3*, *OsMsrA2.2*, *OsMsrA4*, *OsMsrA5*, *OsMsrB1*, *OsMsrB3*, *PtMsrB5*
AS1/AS2 repressor	gctTTGAct	*AtMsrB1*, *AtPMSR1*, *OsMsrA2.2*, *OsMsrA4*, *OsMsrB1*, *OsMsrB3*, *PtMsrA4.2*
NAC with transmembrane motif 1-like 6 (NTL6/NTM1)	tttgttagatatatTAAGaaagg	*AtMsrA5*, *AtMsrB2*, *OsMsrA2.2*, *OsMsrB3*, *OsMsrB5*, *PtMsrA4.2*, *PtMsrA5*, *PtMsrB3.2*
Storekeeper (STK), plant specific DNA binding protein	cccacaTATCcactatt	*AtMsrB3*, *AtMsrB4*, *AtPMSR4*, *OsMsrA2.1*, *OsMsrA2.2*, *OsMsrA5*, *PtMsrA2.1*, *PtMsrB5*
Homeobox protein 34	ataagactTAATgaaaaca	*AtMsrA5*, *AtMsrB1*, *AtMsrB7*, *AtPMSR4*, *OsMsrA2.2*, *OsMsrA5*, *OsMsrB1*, *OsMsrB3*
bZIP protein G-Box binding factor 1	tttcacCACGtcactgctt	*AtMsrA5*, *AtMsrB4*, *AtMsrB4*, *AtPMSR1*, *OsMsrA5*, *OsMsrB5*, *PtMsrA5*, *PtMsrB3.1*, *PtMsrB5*

**Table 3:** List and number (nr) of TFs and REs predicted to interact with the promoter region of one specific *MsrA* or *MsrB* gene originating from the *Arabidopsis thaliana* (At), *Populus trichocarpa* (Pt), and *Oryza sativa* (Os) genomes. The predictions were made using MatInspector software, and intersections of the lists of elements were calculated while using the Venn web tool.

Gene Name	Nr	TFs and REs
*AtPMSR1*	1	NAC domain containing protein 3 (NAC3)
*AtPMSR3*	3	WRKY transcription factor 8 (WRKY8), R2R3-type myb-like transcription factor IIG-type binding site (MYB0), Cooperatively regulated by ethylene and jasmonate 1 (CEJ1)
*AtPMSR4*	2	Myb domain protein 116 (MYB116), RY and Sph motifs conserved in seed-specific promoters
*AtMsrA5*	1	Heat stress transcription factor C-1 (HSFC1)
*AtMsrB1*	1	NAC domain containing protein 45 (NAC045)
*AtMsrB2*	4	Transcription factor TGA3 (TGA3), Arabidopsis NAC domain containing protein 81 (NAC081), ATAF2 WRKY transcription factor 55 (WRKY55)
*AtMsrB6*	1	Indeterminate (ID)-domain 11 (IDD11)
*AtMsrB7*	2	REVEILLE 5 (RVE5), Dof zinc finger protein DOF4.7 AT4G38000 (DOF4.7)
*AtMsrB8*	2	Indeterminate (ID)-domain 5 protein RAVEN (IDD5), ABA-responsive element binding protein 3 (AREB3)
*AtMsrB9*	1	Phytochrome-interacting fator 5 (PIL6)
*PtMsrA2.1*	2	Dof zinc finger protein DOF5.1 AT5G02460 (DOF5.1), Dof zinc finger protein DOF2.2 AT2G28810 (DOF2.2)
*PtMsrA4.1*	1	C-repeat-binding factor 3 (DREB1A)
*PtMsrA4.2*	1	Myb domain protein 118 Plant Growth Activator 37 (MYB118)
*PtMsrA5*	2	B3 domain-containing transcription factor NGA4 (NGA4), Myb domain protein 111 (MYB111)
*PtMsrB3.1*	2	C-repeat-binding factor 2 (DREB1C,) Dof zinc finger protein DOF5.8 AT5G66940 (DOF5.8)
*OsMsrA2.2*	2	Iron-dependent regulatory sequence, TCP class II transcription factor (BRC1)
*OsMsrA4*	3	NAC domain containing protein 96 (NAC096), Ethylene-responsive transcription factor 105 (ERF105), Redox responsive transcription factor 1 (RRTF1)
*OsMsrA5*	8	Ethylene-responsive transcription factor 2 (ERF2), Rice bHLH protein (bHLH39), Ethylene-responsive transcription factor 10 (ERF10), Nodulin consensus sequence 3, Ethylene-responsive transcription factor 115 (ERF115), C-repeat-binding factor 2 (DREB1B), Ethylene-responsive transcription factor 5 (ERF5), TCP domain protein 21 AT5G08330 (TCP21)
*OsMsrB1*	4	Dehydration-responsive element-binding protein 2C (DREB2C), LIM domain protein binding to a PAL-box like sequence (WLIM1), E2F transcription factor 3 (E2F3), BBES1/BZR1-like protein 2 AT4G36780 (BEH2)
*OsMsrB3*	1	Transcription factor TCP15 AT1G69690 (TCP15)
*OsMsrB5*	1	Heat stress transcription factor A-1b (HSF3)

## Supplementary Materials

Supplementary materials can be found at https://www.mdpi.com/1422-0067/20/6/1309/s1.

## Abbreviations

ID	identification number
GO	gene ontology
JA	jasmonic acid
MetSO	methionine sulfoxide
Msr	methionine sulfoxide reductase
MYB	v-myb avian myeloblastosis viral oncogene homolog
NAC	no apical meristem, Arabidopsis transcription activation factor and cup-shaped cotyledon
NCBI	National Center for Biotechnology Information
REs	Responsive elements
TFs	Transcription factors
