# Supplementary material for: Regulation of Gene Expression of Methionine Sulfoxide Reductases and Their New Putative Roles in Plants
Source: Int J Mol Sci. 2019 Mar 15;20(6):1309. doi: 10.3390/ijms20061309 (PMC6471524; doi:10.3390/ijms20061309)
Supplement: Supplementary file 1 [file ijms-20-01309-s001.pdf]

Name*	Locus	Promoter	Transcript
**PMSR1**	**peptidemethionine sulfoxide reductase 1**<br>PMSR1 (GeneID:836286)<br>Chromosome 5, Contig TAIR10_5<br>Synonyms: ARABIDOPSIS THALIANA METHIONINE SULFOXIDE REDUCTASE A1, AT5G61640, ATMSRA1, K11J9.18, K11J9_18, peptidemethionine sulfoxide reductase 1, PMSR1<br>GXL_1314243	GXP_3466370 (+)<br>24773980-24775155 (1176 bp)<br><br>3 coding transcripts	GXT_28720625 (2 exons)<br>NM_001345485 (PMSR1)<br><br>GXT_24712510 (2 exons)<br>NM_125558 (PMSR1)<br>TSS = 1049<br><br>GXT_24723693 (2 exons)<br>AK227434 (PMSR1)<br>TSS = 1076
**PMSR2**	**peptidemethionine sulfoxide reductase 2**<br>PMSR2 (GeneID:830637)<br>Chromosome 5, Contig TAIR10_5<br>Synonyms: ARABIDOPSIS THALIANA METHIONINE SULFOXIDE REDUCTASE 2, AT5G07460, ATMSRA2, peptidemethionine sulfoxide reductase 2, PMSR2, T2I1.170, T2I1_170<br>GXL_1309175	GXP_3460425 (-)<br>2361793-2363228 (1436 bp)<br><br>1 coding transcript	GXT_24688085 (2 exons)<br>NM_120828 (PMSR2)
**PMSR3**	**peptidemethionine sulfoxide reductase 3**<br>PMSR3 (GeneID:830638)<br>Chromosome 5, Contig TAIR10_5<br>Synonyms: ARABIDOPSIS THALIANA METHIONINE SULFOXIDE REDUCTASE 3, AT5G07470, ATMSRA3, peptidemethionine sulfoxide reductase 3, PMSR3, T2I1.180, T2I1_180<br>GXL_1309176	GXP_3460426 (-)<br>2364171-2365479 (1309 bp)<br><br>2 coding transcripts	GXT_24688152 (2 exons)<br>NM_120829 (PMSR3)<br><br>GXT_24720272 (2 exons)<br>AK118353 (PMSR3)<br>TSS = 1209
**PMSR4**	**peptide met sulfoxide reductase 4**<br>PMSR4 (GeneID:828616)<br>Chromosome 4, Contig TAIR10_4<br>Synonyms: AT4G25130, F24A6.2, methionine sulfoxide reductase A4, MSRA4, peptide met sulfoxide reductase 4, PMSR4<br>GXL_1306811	GXP_3457608 (-)<br>12899990-12901090 (1101 bp)<br><br>1 coding transcript	GXT_24698183 (2 exons)<br>NM_118645 (PMSR4)

**MsrA5**	**Peptide methionine sulfoxide reductase family protein**<br>MSRA5 (GeneID:816315)<br>Chromosome 2, Contig TAIR10_2<br>Synonyms: AT2G18030, methionine sulfoxide reductase A5, MSRA5, T27K22.10, T27K22_10<br>GXL_1295342	GXP_3444183 (+)<br>7839037-7840208 (1172 bp)<br><br>3 coding transcripts	GXT_24701888 (4 exons)<br>NM_127359 (MSRA5)
			GXT_24701887 (3 exons)<br>NM_201754 (MSRA5)<br>TSS = 1031
			GXT_24721662 (4 exons)<br>AK227376 (MSRA5)<br>TSS = 1072s
**MsrB1**	**methionine sulfoxide reductase B 1**<br>MSRB1 (GeneID:841804)<br>Chromosome 1, Contig TAIR10_1<br>Synonyms: AT1G53670, ATMSRB1, F22G10.17, F22G10_17, methionine sulfoxide reductase B 1, MSRB1<br>GXL_1291503	GXP_3439680 (+)<br>20035631-20036802 (1172 bp)<br><br>3 coding transcripts	GXT_24712929 (5 exons)<br>NM_104245 (MSRB1)
			GXT_24712930 (5 exons)<br>NM_001124012 (MSRB1)<br>TSS = 1011
			GXT_24721486 (5 exons)<br>AK117314 (MSRB1)<br>TSS = 1072
**MsrB2**	**methionine sulfoxide reductase B 2**<br>MSRB2 (GeneID:828274)<br>Chromosome 4, Contig TAIR10_4<br>Synonyms: AT4G21860, methionine sulfoxide reductase B 2, MSRB2, T8O5.70, T8O5_70<br>GXL_1306445	GXP_3457183 (-)<br>11601429-11602724 (1296 bp)<br><br>5 coding transcripts	GXT_24693439 (3 exons)<br>NM_118306 (MSRB2)
			GXT_24693440 (3 exons)<br>NM_001084954 (MSRB2)<br>TSS = 1148
			GXT_25289186 (4 exons)<br>NM_001203862 (MSRB2)<br>TSS = 1148
			GXT_28708001 (4 exons)<br>NM_001341518 (MSRB2)<br>TSS = 1148
			GXT_24718961 (3 exons)<br>AK316971 (MSRB2)<br>TSS = 1196
**MsrB3**	**methionine sulfoxide reductase B3**<br>MSRB3 (GeneID:825817)<br>Chromosome 4, Contig TAIR10_4<br>Synonyms: AT4G04800, ATMSRB3, methionine	GXP_3455236 (+)<br>2438520-2439620 (1101 bp)<br><br>1 coding transcript	GXT_24715441 (3 exons)<br>NM_116718 (MSRB3)

	sulfoxide reductase B3, MSRB3, T4B21.6, T4B21_6<br>GXL_1304769		
**MsrB4**	**methionine sulfoxide reductase B4**<br>MSRB4 (GeneID:825818)<br>Chromosome 4, Contig TAIR10_4<br>Synonyms: AT4G04810, ATMSRB4, methionine sulfoxide reductase B4, MSRB4, T4B21.22, T4B21_22<br>GXL_1304770	GXP_3455238 (+)<br>2440241-2441341 (1101 bp)<br><br>2 coding transcripts	GXT_24715521 (3 exons)<br>NM_116719 (MSRB4)<br><br>GXT_28717681 (2 exons)<br>NM_001340514 (MSRB4)
**MsrB5**	**methionine sulfoxide reductase B5**<br>MSRB5 (GeneID:825820)<br>Chromosome 4, Contig TAIR10_4<br>Synonyms: AT4G04830, ATMSRB5, methionine sulfoxide reductase B5, MSRB5, T4B21.5, T4B21_5<br>GXL_1304771	GXP_3455239 (+)<br>2444775-2445894 (1120 bp)<br><br>2 coding transcripts	GXT_24715522 (3 exons)<br>NM_116721 (MSRB5)<br><br>GXT_25290545 (3 exons)<br>NM_001203745 (MSRB5)<br>TSS = 1020
**MsrB6**	**methionine sulfoxide reductase B6**<br>MSRB6 (GeneID:825821)<br>Chromosome 4, Contig TAIR10_4<br>Synonyms: AT4G04840, ATMSRB6, methionine sulfoxide reductase B6, MSRB6, T4B21.4, T4B21_4<br>GXL_1304772	GXP_3455240 (+)<br>2448434-2449660 (1227 bp)<br><br>2 coding transcripts	GXT_24715384 (4 exons)<br>NM_116722 (MSRB6)<br><br>GXT_24723339 (4 exons)<br>AK118878 (MSRB6)<br>TSS = 1127
**MsrB7**	**methionine sulfoxide reductase B7**<br>MSRB7 (GeneID:828271)<br>Chromosome 4, Contig TAIR10_4<br>Synonyms: AT4G21830, ATMSRB7, methionine sulfoxide reductase B7, MSRB7, T8O5.40, T8O5_40<br>GXL_1306442	GXP_3457179 (-)<br>11585291-11586542 (1252 bp)<br><br>2 coding transcripts	GXT_24693545 (3 exons)<br>NM_118303 (MSRB7)<br><br>GXT_24693546 (4 exons)<br>NM_001084953 (MSRB7)<br>TSS = 1152
**MsrB8**	**methionine sulfoxide reductase B8**<br>MSRB8 (GeneID:828272)<br>Chromosome 4, Contig TAIR10_4<br>Synonyms: AT4G21840, ATMSRB8, methionine sulfoxide reductase B8, MSRB8, T8O5.50, T8O5_50<br>GXL_1306443	GXP_3457180 (-)<br>11588273-11589373 (1101 bp)<br><br>1 coding transcript	GXT_24693377 (3 exons)<br>NM_118304 (MSRB8)
**MsrB9**	**methionine sulfoxide reductase B9**<br>MSRB9 (GeneID:828273)	GXP_3457181 (-)<br>11592138-11593425 (1288 bp)	GXT_24693438 (3 exons)<br>NM_118305 (MSRB9)

	Chromosome 4, Contig TAIR10_4<br>Synonyms: AT4G21850, ATMSRB9, methionine sulfoxide reductase B9, MSRB9, T8O5.60, T8O5_60<br>GXL_1306444	2 coding transcripts	GXT_24693437 (2 exons)<br>NM_179087 (MSRB9)<br>TSS = 1188

Table S1A The list of 5 isoforms of methionine sulfoxide reductase (Msr) type A genes and 9 isoforms of B-type Msr (MsrB) genes originated form the *Arabidopsis thaliana* genome. Gene sequences were derived from the TAIR 10 database (https://www.arabidopsis.org/). For each gene the full name, accession number (ID), chromosome location, and all synonyms are given. For each gene all promoters were extracted directly from the ElDorado genome database (https://www.genomatix.de/online_help/help_eldorado/introduction.html). The location and length of each promoter accompanied by the number of encoded transcripts are specified. For each sequence analyzed the transcript start site (TSS) is 1001 unless specified otherwise. Each transcript is characterized with the number of exons and accession number to specific mRNA sequence in National Center for Biotechnology Information database (https://www.ncbi.nlm.nih.gov).

Name*	Locus	Promoter	Transcript
**MsrA2.1**	**methionine sulfoxide reductase family protein**<br>POPTR_0012s09290g (GeneID:7484440);<br>Chromosome LGXII , Contig NC_008478<br>Synonyms: POPTRDRAFT_422117,<br>POPTR_0012s09290g<br>GXL_914392	GXP_2492359 (-)<br>10326644-10327744 (1101 bp)<br><br>1 coding transcript	GXT_23681117 (2 exons)<br>XM_002318656 (POPTR_0012s09290g)
**MsrA2.2**	**hypothetical protein**<br>POPTR_0015s09910g (GeneID:18105852)<br>Chromosome LGXV, Contig NC_008481<br>Synonyms: POPTR_0015s09910g<br>GXL_1953678	GXP_5576574 (-)<br>10974177-10975277 (1101 bp)<br><br>1 coding transcript	GXT_26747626 (2 exons)<br>XM_006374483 (POPTR_0015s09910g)
**MsrA4.1**	**hypothetical protein**<br>POPTR_0015s12390g (GeneID:7457599)<br>Chromosome LGXV, Contig NC_008481<br>Synonyms: POPTRDRAFT_824833,<br>POPTR_0015s12390g<br>GXL_918998	GXP_2497451 (-)<br>12656397-12657497 (1101 bp)<br><br>1 coding transcript	GXT_23685395 (2 exons)<br>XM_002322287 (POPTR_0015s12390g)
**MsrA4.2**	**hypothetical protein**<br>POPTR_0012s11630g (GeneID:18103860)<br>Chromosome LGXII, Contig NC_008478<br>Synonyms: POPTR_0012s11630g<br>GXL_2308570	GXP_7080902 (-)<br>11980041-11981141 (1101 bp)<br><br>1 coding transcript	GXT_28080054 (2 exons)<br>XM_006376903 (POPTR_0012s11630g)
**MsrA5**	**peptide methionine sulfoxide reductase family protein**<br>POPTR_0007s14050g (GeneID:7495540)<br>Chromosome LGVII, Contig NC_008473<br>Synonyms: POPTRDRAFT_655450,<br>POPTR_0007s14050g<br>GXL_908130	GXP_2485511 (+)<br>13975396-13976496 (1101 bp)<br><br>1 coding transcript	GXT_23688611 (4 exons)<br>XM_002310268 (POPTR_0007s14050g)
**MsrB1**	**hypothetical protein**<br>POPTR_0011s11490g (GeneID:7462308)<br>Chromosome LGXI, Contig NC_008477<br>Synonyms: POPTRDRAFT_568844,<br>POPTR_0011s11490g<br>GXL_2308225	GXP_7080439 (+)<br>14233440-14234540 (1101 bp)<br><br>2 coding transcripts	GXT_28087242 (3 exons)<br>XM_006377694 (POPTR_0011s11490g)<br><br>GXT_28087243 (4 exons)<br>XM_002316839 (POPTR_0011s11490g)

Table S1B

**MsrB3.1**	**hypothetical protein**<br>POPTR_0001s29320g (GeneID:7470782)<br>Chromosome LGI, Contig NC_008467<br>Synonyms: POPTRDRAFT_707507,<br>POPTR_0001s29320g<br>GXL_895645	GXP_2471852 (+)<br>28036075-28037175 (1101 bp)<br><br>2 coding transcripts	GXT_26739897 (2 exons)<br>XM_006369640 (POPTR_0001s29320g)<br><br>GXT_23675830 (3 exons)<br>XM_002300270 (POPTR_0001s29320g)
**MsrB3.2**	**hypothetical protein**<br>POPTR_0009s08420g (GeneID:7469676)<br>Chromosome LGIX, Contig NC_008475<br>Synonyms: POPTRDRAFT_767763,<br>POPTR_0009s08420g<br>GXL_902769	GXP_2479686 (+)<br>7712847-7713947 (1101 bp)<br><br>2 coding transcripts	GXT_26745176 (3 exons)<br>XM_006379082 (POPTR_0009s08420g)<br><br>GXT_23679128 (3 exons)<br>XM_002313915 (POPTR_0009s08420g)
**MsrB5**	**hypothetical protein**<br>POPTR_0008s20300g (GeneID:7454233)<br>Chromosome LGVIII, Contig NC_008474<br>Synonyms: POPTRDRAFT_565286,<br>POPTR_0008s20300g<br>GXL_910001	GXP_2487518 (-)<br>14053984-14055084 (1101 bp)<br><br>1 coding transcript	GXT_23679962 (3 exons)<br>XM_002312688 (POPTR_0008s20300g)

Table S1B The list of 5 isoforms of methionine sulfoxide reductase (Msr) type A genes and 4 isoforms of B-type Msr (MsrB) genes originated form the *Populus trichocarpa* genome. Gene sequences were derived from the using Poptr 2.0 database (https://phytozome.jgi.doe.gov/pz/portal.html#!info?alias=Org_Ptrichocarpa). For each gene the full name, accession number (ID), chromosome location, and all synonyms are given. For each gene all promoters were extracted directly from the ElDorado genome database (https://www.genomatix.de/online_help/help_eldorado/introduction.html). The location and length of each promoter accompanied by the number of encoded transcripts are specified. For each sequence analyzed the transcript start site (TSS) is 1001 unless specified otherwise. Each transcript is characterized with the number of exons and accession number to specific mRNA sequence in National Center for Biotechnology Information database (https://www.ncbi.nlm.nih.gov).

Gene name	Locus	Promoter	Transcript
MsrA2.1	**peptide methionine sulfoxide reductase A2-1**<br>LOC4336192 (GeneID:4336192)<br>Chromosome 4, Contig 13104<br>Synonyms: LOC4336192, MSRA2-1, OsJ_15220, OsMSRA2.1<br>GXL_810337	GXP_2162925 (-)<br>24104768-24105933 (1166 bp)<br><br>2 coding transcripts	GXT_28014730 (2 exons)<br>XM_015781799 (LOC4336192)<br><br>GXT_23452618 (2 exons)<br>AK064801 (LOC4336192)<br>TSS = 1066
MsrA2.2	**peptide methionine sulfoxide reductase A2-2**<br>LOC107277650 (GeneID:107277650)<br>Chromosome 4, Contig 13104<br>Synonyms: LOC107277650, MSRA2-2, OsMSRA2.2<br>GXL_2292712	GXP_7016752 (-)<br>24110771-24111871 (1101 bp)<br><br>1 coding transcript	GXT_28013482 (3 exons)<br>XM_015779606 (LOC107277650)
MsrA4	**peptide methionine sulfoxide reductase A4, chloroplastic**<br>LOC4349402 (GeneID:4349402)<br>Chromosome 10, Contig 13110<br>Synonyms: LOC4349402, MSRA4, OsMSRA4<br>GXL_793939	GXP_2144455 (+)<br>22253764-22254987 (1224 bp)<br><br>2 coding transcripts	GXT_28048001 (2 exons)<br>XM_015758657 (LOC4349402)<br><br>GXT_23466560 (2 exons)<br>AK067167 (LOC4349402)<br>TSS = 1020<br><br>GXT_23472590 (1 exon)<br>AK109715 (LOC4349402)<br>TSS = 1124
MsrA5	**peptide methionine sulfoxide reductase A5**<br>LOC4340052 (GeneID:4340052)<br>Chromosome 6, Contig 13106<br>Synonyms: LOC4340052, MSRA5, OsMSRA5<br>GXL_814325	GXP_2167398 (-)<br>2029286-2031318 (2033 bp)<br><br>2 coding transcripts	GXT_28017847 (4 exons)<br>XM_015787395 (LOC4340052)<br>TSS = 1018<br><br>GXT_23453583 (4 exons)<br>AK069328 (LOC4340052)<br>TSS = 1108
MsrB1	**peptide methionine sulfoxide reductase B1, chloroplastic**<br>LOC4341018 (GeneID:4341018)<br>Chromosome 6, Contig 13106<br>Synonyms: LOC4341018, MSRB1, OsJ_21331, OsMSRB1<br>GXL_815326	GXP_2168518 (-)<br>15721109-15722368 (1260 bp)<br><br>1 coding transcript	GXT_28018647 (5 exons)<br>XM_015788787 (LOC4341018)

Table S1C

MsrB3	**peptide methionine sulfoxide reductase B3, chloroplastic**<br>LOC4338744 (GeneID:4338744)<br>Chromosome 5, Contig 13105<br>Synonyms: LOC4338744, MSRB3, OsMSRB3<br>GXL_812980	GXP_2165891 (+)<br>19680750-19681945 (1196 bp)<br><br>3 coding transcripts	GXT_28039792 (3 exons)<br>XM_015781814 (LOC4338744)
			GXT_23470549 (3 exons)<br>AK071730 (LOC4338744)<br>TSS = 1019
			GXT_23470404 (3 exons)<br>AK104169 (LOC4338744)<br>TSS = 1023
MsrB5	**peptide methionine sulfoxide reductase B5**<br>LOC4332888 (GeneID:4332888)<br>Chromosome 3, Contig 13103<br>Synonyms: LOC4332888, MSRB5, OsJ_10923, OsMSRB5<br>GXL_807000	GXP_2159172 (+)<br>14009172-14010318 (1147 bp)<br><br>2 coding transcripts	GXT_28037587 (3 exons)<br>XM_015777603 (LOC4332888)
			GXT_23469473 (3 exons)<br>AK068764 (LOC4332888)<br>TSS = 1042

Table S1C The list of 4 isoforms of methionine sulfoxide reductase (Msr) type A genes and 3 isoforms of B-type Msr (MsrB) genes originated form *Oryza sativa* genome. Gene sequences were derived from the MSU release 7 database (http://www.plantgdb.org/OsGDB/). For each gene the full name, accession number (ID), chromosome location, and all synonyms are given. For each gene all promoters were extracted directly from the ElDorado genome database (https://www.genomatix.de/online_help/help_eldorado/introduction.html). The location and length of each promoter accompanied by the number of encoded transcripts are specified. For each sequence analyzed the transcript start site (TSS) is 1001 unless specified otherwise. Each transcript is characterized with the number of exons and accession number to specific mRNA sequence in National Center for Biotechnology Information database (https://www.ncbi.nlm.nih.gov).

Table S2A List of TFs and REs predicted to interact with the promoter region of one specific type (A or B) of *Msr* gene originating from the *Arabidopsis thaliana*, *Populus trichocarpa*, and *Oryza sativa* genomes. The predictions were made using MatInspector software, and the intersections of the 2 lists of elements were calculated using the Venn web tool.

	Type-specific TFs and REs
A	Ethylene-responsive transcription factor ERF014 (AT1G44830), Putative cis-acting element on various PAL and 4CL gene promoters, Ethylene-responsive transcription factor 2, Rice bHLH protein, WRKY transcription factor 8, NAM-like protein CUP-SHAPED COTYLEDON 1, BES1-interacting Myc-like protein 1, B3 domain-containing transcription factor NGA4, Myb domain protein 118 (Plant Growth Activator 37, PGA37), WRKY DNA-binding protein 40, Ethylene-responsive transcription factor 10, Mitochondrial dysfunction motif Myb domain protein 74, KNOTTED1 (KN1) and KNOTTED interacting protein (KIP) are TALE class homeodomain proteins - the KN1-KIP complex binds this DNA motif with high affinity, RNA polymerase III transcription initiation factor complex (TFIIIC), NAC domain containing protein 96, Cytokinin Response Factor 10, Nodulin consensus sequence 3, Sugar responsive elements Myb domain protein 116, Ethylene-responsive transcription factor 105, VIRE2-interacting protein 1, Ethylene-responsive transcription factor 115, R2R3-type myb-like transcription factor (IIG-type binding site), Heat stress transcription factor C-1, C-repeat-binding factor 2 (DREB1B), Cooperatively regulated by ethylene and jasmonate 1 (DEAR1), Transcription factor TCP23, Ethylene-responsive transcription factor 5, Myb domain protein 111 (secondary DNA binding preference), Iron-dependent regulatory sequence, BES1-interacting Myc-like protein 2, Ethylene-responsive transcription factor ERF112 (AT2G33710), RY and Sph motifs conserved in seed-specific promoters, TCP domain protein 7, TCP domain protein 21 (AT5G08330), C-repeat-binding factor 3 (DREB1A), TCP class II transcription factor, Ethylene-responsive transcription factor ERF019 (AT1G22810), Ethylene-responsive transcription factor ERF017 (AT1G19210), NAC (No Apical Meristem) domain transcriptional regulator superfamily protein, Ethylene-responsive transcription factor 15 (AT2G31230), Dof zinc finger protein DOF5.1 (AT5G02460), NAC domain containing protein 3 (ORE1 SISTER1), Redox responsive transcription factor 1 (secondary DNA binding preference), Ethylene-responsive transcription factor ERF018 (secondary DNA binding preference), Dof zinc finger protein DOF2.2 (AT2G28810)
B	Myb domain protein 119, Heat stress transcription factor A-1b (HSF3), Transcription factor TGA3, Indeterminate(ID)-domain 5 protein (RAVEN), BES1/BZR1 homolog 4 (AT1G78700), WRKY DNA binding protein 38, OCS-like elements, CUP-SHAPED COTYLEDON 2, HRS1 Homolog 3 (AT1G25550), REVEILLE 5, C-repeat-binding factor 2 (DREB1C), Dehydration-responsive element-binding protein 2C (secondary DNA binding preference), LIM domain protein binding to a PAL-box like sequence, Arabidopsis NAC domain containing protein 81; ATAF2 Dof zinc finger protein DOF4.7 (AT4G38000), ABA-responsive element binding protein 3, WRKY DNA binding protein 12, Homeobox-leucine zipper protein HAT2, Indeterminate(ID)-domain 11 (AtIDD11), WRKY transcription factor 55, Homeodomain protein of the Knotted class1, Phytochrome-interacting fator 5 (PIL6), NAC domain containing protein 45, Transcription factor TCP15 (AT1G69690), Dof zinc finger protein DOF5.8 (AT5G66940), E2F transcription factor 3, NTM1-LIKE 6 (NAC domain containing protein 62), Homeobox-leucine zipper protein ATHB-20, Vascular related NAC-domain protein 1, BBES1/BZR1-like protein 2 (AT4G36780), Silencing element binding factor - transcriptional repressor Myb domain protein 55

Table S2B List of TFs and REs predicted to interact with the promoter region of *Msrs* originating from one of the three studied species, i.e., *Arabidopsis thaliana* (At), *Populus trichocarpa* (Pt), and *Oryza sativa* (Os). The predictions were made using MatInspector software, and the intersections of the 3 lists of elements were calculated using the Venn web tool.

	Species-specific TFs and REs
**At**	WRKY transcription factor 8, NAM-like protein CUP-SHAPED COTYLEDON 1, Transcription factor TGA3, Indeterminate(ID)-domain 5 protein (RAVEN), BES1/BZR1 homolog 4 (AT1G78700), Dwarf and Delayed Flowering 2, WRKY DNA-binding protein 18, CUP-SHAPED COTYLEDON 2, REVEILLE 5 CUP-SHAPED COTYLEDON 3, ANT (Arabidopsis protein AINTEGUMENTA) member of the plant-specific family of AP2/EREBP-transcription factors, Arabidopsis NAC domain containing protein 81; ATAF2 Dof zinc finger protein DOF4.7 (AT4G38000), ABA-responsive element binding protein 3, RNA polymerase III transcription initiation factor complex (TFIIIC), Indeterminate(ID)-domain 11 (AtIDD11), NAC domain containing protein 2, WRKY transcription factor 55, DNA-binding protein S1FA3 HBP-1a, suggested to be involved in the cell cycle-dependent expression, Myb domain protein 116, Phytochrome-interacting fator 5 (PIL6), R2R3-type myb-like transcription factor (IIG-type binding site), Heat stress transcription factor C-1, Cooperatively regulated by ethylene and jasmonate 1 (DEAR1), NAC domain containing protein 45, BES1-interacting Myc-like protein 2 RY and Sph motifs conserved in seed-specific promoters, NAC (No Apical Meristem) domain transcriptional regulator superfamily protein, NTM1-LIKE 6 (NAC domain containing protein 62), NAC domain containing protein 3 (ORE1 SISTER1)
**Pt**	Ethylene-responsive transcription factor ERF014 (AT1G44830), B3 domain-containing transcription factor NGA4, Myb domain protein 118 (Plant Growth Activator 37, PGA37), C-repeat-binding factor 2 (DREB1C) Myb domain protein 111 (secondary DNA binding preference), Dof zinc finger protein DOF5.8 (AT5G66940), C-repeat-binding factor 3 (DREB1A), Dof zinc finger protein DOF5.1 (AT5G02460), Dof zinc finger protein DOF2.2 (AT2G28810)
**Os**	Ethylene-responsive transcription factor RAP2-3 (secondary DNA binding preference), Ethylene-responsive transcription factor 2, LOB domain-containing protein 13, Heat stress transcription factor A-1b (HSF3), Wheat NAC-domain DNA binding factor (DNA binding site I), Rice bHLH protein Ethylene-responsive transcription factor RAP2-6, Ethylene-responsive transcription factor 10, Redox responsive transcription factor 1, Dehydration-responsive element-binding protein 2G (AT5G18450), Dehydration-responsive element-binding protein 2C (secondary DNA binding preference), Ethylene-responsive transcription factor ERF057 (AT5G65130), LIM domain protein binding to a PAL-box like sequence, Target of early activation tagged (EAT) 2, Ethylene-responsive transcription factor 1B (AT3G23240), Ethylene-responsive transcription factor 1A (AT4G17500), NAC domain containing protein 96, Cytokinin Response Factor 10, Ethylene-responsive transcription factor 8 FYF Up-regulating 321 Factor 1 (AT1G71450), Nodulin consensus sequence 3, Ethylene-responsive transcription factor 105, Ethylene-responsive transcription factor 115, C-repeat-binding factor 2 (DREB1B), Transcription factor TCP23, Transcription factor TCP15 (AT1G69690), Ethylene-responsive transcription factor 5, TCP class I transcription factor X gene core promoter element 1, Iron-dependent regulatory sequence, Ethylene and salt inducible 3, Transcription factor TGA2, Human motif ten element, TCP domain protein 21 (AT5G08330), TCP class II transcription factor, FAR-RED Impaired Response 1, Ethylene-responsive transcription factor ERF019 (AT1G22810), E2F transcription factor 3, Ethylene-responsive transcription factor ERF017 (AT1G19210), Phytochrome B-regulated transcription factor PIF3 Ethylene-responsive transcription factor 15 (AT2G31230), Dehydration-responsive element-binding protein 2C, Redox responsive transcription factor 1 (secondary DNA binding preference), Transcription factor II B (TFIIB) recognition element, BBES1/BZR1-like protein 2 (AT4G36780), Ethylene-responsive transcription factor ERF018 (secondary DNA binding preference), Ethylene-responsive transcription factor RAP2-11

Table S2C The list of transcription factors and responsive elements predicted to bind the promoter region of each isoform of methionine sulfoxide reductase type A and B of three species: *Arabidopsis thaliana*, *Populus trichocarpa*, and *Oryza sativa*. The TFs were predicted using MatInspector software and the intersections of 30 lists of elements were calculated using the Venn web tool.

Gene Name	total	Transcription factor or responsive element
AtMsrA5 AtMsrB1 AtMsrB2 AtMsrB3 AtMsrB4 AtMsrB5 AtMsrB6 AtMsrB7 AtMsrB8 AtMsrB9 AtPMSR1 AtPMSR2 AtPMSR3 AtPMSR4 OsMsrA2.1 OsMsrA4 OsMsrA5 OsMsrB1 OsMsrB3 OsMsrB5 PtMsrA2.1 PtMsrA2.2 PtMsrA4.1 PtMsrA4.2 PtMsrA5 PtMsrB1 PtMsrB3.1 PtMsrB3.2 PtMsrB5	1	Protodermal factor 2
AtMsrA5 AtMsrB1 AtMsrB2 AtMsrB3 AtMsrB4 AtMsrB5 AtMsrB6 AtMsrB7 AtMsrB8 AtMsrB9 AtPMSR1 AtPMSR2 AtPMSR3 AtPMSR4 OsMsrA2.1 OsMsrA4 OsMsrA5 OsMsrB3 OsMsrB5 PtMsrA2.1 PtMsrA2.2 PtMsrA4.1 PtMsrA4.2 PtMsrA5 PtMsrB1 PtMsrB3.1 PtMsrB3.2 PtMsrB5	1	Jasmonate response element
AtMsrA5 AtMsrB1 AtMsrB2 AtMsrB3 AtMsrB4 AtMsrB5 AtMsrB6 AtMsrB7 AtMsrB8 AtMsrB9 AtPMSR1 AtPMSR2 AtPMSR3 AtPMSR4 OsMsrA2.1 OsMsrA4 OsMsrB1 OsMsrB5 PtMsrA2.1 PtMsrA2.2 PtMsrA4.1 PtMsrA4.2 PtMsrA5 PtMsrB1 PtMsrB3.1 PtMsrB3.2 PtMsrB5	1	SBF-1
AtMsrA5 AtMsrB1 AtMsrB2 AtMsrB3 AtMsrB4 AtMsrB5 AtMsrB6 AtMsrB7 AtMsrB8 AtMsrB9 AtPMSR1 AtPMSR2 AtPMSR3 AtPMSR4 OsMsrA2.1 OsMsrA4 OsMsrA5 OsMsrB1 OsMsrB3 OsMsrB5 PtMsrA2.1 PtMsrA4.1 PtMsrA4.2 PtMsrA5 PtMsrB1 PtMsrB3.1 PtMsrB3.2	1	Homeodomain protein WUSCHEL
AtMsrA5 AtMsrB1 AtMsrB2 AtMsrB3 AtMsrB4 AtMsrB5 AtMsrB6 AtMsrB7 AtMsrB8 AtMsrB9 AtPMSR2 AtPMSR3 AtPMSR4 OsMsrA2.1 OsMsrA4 OsMsrA5 OsMsrB3 OsMsrB5 PtMsrA2.1 PtMsrA2.2 PtMsrA4.1 PtMsrA4.2 PtMsrA5 PtMsrB1 PtMsrB3.1 PtMsrB3.2 PtMsrB5	1	Homeodomain GLABROUS 1
AtMsrA5 AtMsrB1 AtMsrB2 AtMsrB3 AtMsrB4 AtMsrB5 AtMsrB6 AtMsrB7 AtMsrB8 AtMsrB9 AtPMSR1 AtPMSR2 AtPMSR3 AtPMSR4 OsMsrA2.1 OsMsrB3 OsMsrB5 PtMsrA2.1 PtMsrA2.2 PtMsrA4.1 PtMsrA4.2 PtMsrA5 PtMsrB1 PtMsrB3.1 PtMsrB3.2 PtMsrB5	1	Plant TATA box
AtMsrA5 AtMsrB1 AtMsrB2 AtMsrB3 AtMsrB5 AtMsrB6 AtMsrB7 AtMsrB8 AtMsrB9 AtPMSR1 AtPMSR2 AtPMSR3 AtPMSR4 OsMsrA2.1 OsMsrA4 OsMsrB1 OsMsrB3 OsMsrB5 PtMsrA2.1 PtMsrA2.2 PtMsrA4.1 PtMsrA4.2 PtMsrB1 PtMsrB3.1 PtMsrB3.2 PtMsrB5	1	TESMIN/TSO1-like CXC 2
AtMsrA5 AtMsrB1 AtMsrB2 AtMsrB4 AtMsrB5 AtMsrB6 AtMsrB7 AtMsrB8 AtMsrB9 AtPMSR1 AtPMSR2 AtPMSR3 AtPMSR4 OsMsrA2.1 OsMsrA4 OsMsrA5 OsMsrB1 OsMsrB5 PtMsrA2.1 PtMsrA2.2 PtMsrA4.1 PtMsrA4.2 PtMsrA5 PtMsrB1 PtMsrB3.2 PtMsrB5	1	KANADI box
AtMsrA5 AtMsrB1 AtMsrB2 AtMsrB3 AtMsrB4 AtMsrB5 AtMsrB6 AtMsrB7 AtMsrB8 AtMsrB9 AtPMSR1 AtPMSR2 AtPMSR3 AtPMSR4 OsMsrA2.1 OsMsrA4 OsMsrA5 PtMsrA2.1 PtMsrA2.2 PtMsrA4.1 PtMsrA5 PtMsrB1 PtMsrB3.1 PtMsrB3.2 PtMsrB5	1	G2-like family protein

AtMsrA5 AtMsrB1 AtMsrB2 AtMsrB3 AtMsrB4 AtMsrB5 AtMsrB6 AtMsrB8 AtMsrB9 AtPMSR1 AtPMSR2 AtPMSR3 AtPMSR4 OsMsrA2.1 OsMsrA2.2 OsMsrA4 OsMsrA5 OsMsrB1 OsMsrB5 PtMsrA2.1 PtMsrA4.1 PtMsrB1 PtMsrB3.1 PtMsrB3.2 PtMsrB5	1	Stomatal Carpenter 1 (dof5.7) (secondary DNA binding preference)
AtMsrA5 AtMsrB1 AtMsrB2 AtMsrB3 AtMsrB4 AtMsrB5 AtMsrB7 AtMsrB8 AtMsrB9 AtPMSR1 AtPMSR2 AtPMSR3 AtPMSR4 OsMsrA4 OsMsrB3 OsMsrB5 PtMsrA2.1 PtMsrA2.2 PtMsrA4.1 PtMsrA4.2 PtMsrA5 PtMsrB1 PtMsrB3.1 PtMsrB3.2 PtMsrB5	1	DIVARICATA 1 (myb related R-R-type factor AT5G58900)
AtMsrA5 AtMsrB1 AtMsrB2 AtMsrB3 AtMsrB5 AtMsrB6 AtMsrB7 AtMsrB8 AtMsrB9 AtPMSR1 AtPMSR2 AtPMSR3 AtPMSR4 OsMsrA2.1 OsMsrA4 OsMsrB1 PtMsrA2.1 PtMsrA2.2 PtMsrA4.1 PtMsrA4.2 PtMsrA5 PtMsrB1 PtMsrB3.1 PtMsrB3.2 PtMsrB5	1	Trihelix transcription factor GT-1
AtMsrA5 AtMsrB1 AtMsrB3 AtMsrB4 AtMsrB6 AtMsrB7 AtMsrB8 AtMsrB9 AtPMSR1 AtPMSR2 AtPMSR3 OsMsrA2.1 OsMsrA4 OsMsrA5 OsMsrB3 OsMsrB5 PtMsrA2.1 PtMsrA2.2 PtMsrA4.1 PtMsrA4.2 PtMsrA5 PtMsrB1 PtMsrB3.1 PtMsrB3.2 PtMsrB5	1	Transcriptional repressor BELLRINGER
AtMsrA5 AtMsrB1 AtMsrB2 AtMsrB3 AtMsrB5 AtMsrB6 AtMsrB7 AtMsrB8 AtMsrB9 AtPMSR1 AtPMSR4 OsMsrA2.1 OsMsrA4 OsMsrB1 OsMsrB3 OsMsrB5 PtMsrA2.1 PtMsrA2.2 PtMsrA4.1 PtMsrA4.2 PtMsrA5 PtMsrB1 PtMsrB3.1 PtMsrB3.2 PtMsrB5	1	Nodulin consensus sequence 1
AtMsrA5 AtMsrB1 AtMsrB2 AtMsrB3 AtMsrB5 AtMsrB6 AtMsrB7 AtMsrB8 AtMsrB9 AtPMSR1 AtPMSR2 AtPMSR3 AtPMSR4 OsMsrA2.1 OsMsrA2.2 OsMsrA5 OsMsrB3 OsMsrB5 PtMsrA2.1 PtMsrA2.2 PtMsrA4.1 PtMsrA4.2 PtMsrB1 PtMsrB3.2	1	Cis-element in the GAPDH promoters conferring light inducibility
AtMsrA5 AtMsrB2 AtMsrB3 AtMsrB4 AtMsrB5 AtMsrB6 AtMsrB7 AtMsrB9 AtPMSR1 AtPMSR2 AtPMSR3 AtPMSR4 OsMsrA2.1 OsMsrA4 OsMsrB5 PtMsrA2.1 PtMsrA2.2 PtMsrA4.1 PtMsrA4.2 PtMsrA5 PtMsrB1 PtMsrB3.1 PtMsrB3.2 PtMsrB5	1	RAP2.2, involved in carotenoid and tocopherol biosynthesis and in the expression of photosynthesis-related genes
AtMsrA5 AtMsrB1 AtMsrB3 AtMsrB4 AtMsrB5 AtMsrB8 AtMsrB9 AtPMSR1 AtPMSR2 AtPMSR4 OsMsrA2.1 OsMsrA4 OsMsrA5 OsMsrB1 OsMsrB3 OsMsrB5 PtMsrA2.1 PtMsrA2.2 PtMsrA4.2 PtMsrA5 PtMsrB1 PtMsrB3.1 PtMsrB3.2 PtMsrB5	1	DNA-binding storekeeper protein-related transcriptional regulator (AT4G00250)
AtMsrA5 AtMsrB1 AtMsrB2 AtMsrB3 AtMsrB4 AtMsrB5 AtMsrB7 AtMsrB8 AtMsrB9 AtPMSR1 AtPMSR3 AtPMSR4 OsMsrA2.1 OsMsrA4 OsMsrB3 OsMsrB5 PtMsrA2.1 PtMsrA2.2 PtMsrA4.1 PtMsrA4.2 PtMsrA5 PtMsrB1 PtMsrB3.1 PtMsrB3.2	1	Class I GATA factors
AtMsrA5 AtMsrB1 AtMsrB2 AtMsrB3 AtMsrB4 AtMsrB5 AtMsrB6 AtMsrB7 AtMsrB8 AtMsrB9 AtPMSR2 AtPMSR3 AtPMSR4 OsMsrA2.1 OsMsrA5	1	Homeobox protein 32

OsMsrB1 OsMsrB5 PtMsrA2.1 PtMsrA2.2 PtMsrA4.1 PtMsrA4.2 PtMsrA5 PtMsrB3.1 PtMsrB3.2		
AtMsrA5 AtMsrB1 AtMsrB2 AtMsrB4 AtMsrB5 AtMsrB6 AtMsrB7 AtMsrB8 AtMsrB9 AtPMSR2 AtPMSR3 AtPMSR4 OsMsrA2.1 OsMsrA4 OsMsrB1 OsMsrB3 OsMsrB5 PtMsrA2.1 PtMsrA2.2 PtMsrA4.2 PtMsrA5 PtMsrB3.1 PtMsrB3.2 PtMsrB5	1	Avian C-type LTR TATA box
AtMsrA5 AtMsrB1 AtMsrB2 AtMsrB5 AtMsrB6 AtMsrB7 AtMsrB8 AtMsrB9 AtPMSR1 AtPMSR2 AtPMSR3 AtPMSR4 OsMsrA2.2 OsMsrB1 OsMsrB3 OsMsrB5 PtMsrA2.1 PtMsrA4.1 PtMsrA4.2 PtMsrA5 PtMsrB1 PtMsrB3.1 PtMsrB5	1	Myb-like protein of Petunia hybrida
AtMsrA5 AtMsrB1 AtMsrB3 AtMsrB4 AtMsrB5 AtMsrB6 AtMsrB7 AtMsrB8 AtMsrB9 AtPMSR1 AtPMSR2 AtPMSR3 AtPMSR4 OsMsrA4 OsMsrA5 OsMsrB3 PtMsrA2.1 PtMsrA2.2 PtMsrA4.1 PtMsrA5 PtMsrB1 PtMsrB3.1 PtMsrB3.2	1	Heterodimer of NAC-domain transcription factors GmNAC30 and GmNAC81
AtMsrB1 AtMsrB2 AtMsrB5 AtMsrB6 AtMsrB7 AtMsrB8 AtMsrB9 AtPMSR1 AtPMSR2 AtPMSR3 AtPMSR4 OsMsrA2.1 OsMsrA5 OsMsrB1 OsMsrB3 OsMsrB5 PtMsrA2.1 PtMsrA2.2 PtMsrA4.1 PtMsrA4.2 PtMsrA5 PtMsrB1 PtMsrB3.1	1	Myb domain protein 96 (MYBCOV1)
AtMsrB2 AtMsrB3 AtMsrB5 AtMsrB6 AtMsrB7 AtMsrB8 AtMsrB9 AtPMSR1 AtPMSR2 AtPMSR3 AtPMSR4 OsMsrA2.1 OsMsrA4 OsMsrB1 OsMsrB5 PtMsrA2.1 PtMsrA2.2 PtMsrA4.1 PtMsrA4.2 PtMsrA5 PtMsrB1 PtMsrB3.1 PtMsrB3.2	1	Late elongated hypocotyl 1
AtMsrA5 AtMsrB1 AtMsrB2 AtMsrB3 AtMsrB5 AtMsrB7 AtMsrB9 AtPMSR1 AtPMSR2 AtPMSR3 OsMsrA4 OsMsrA5 OsMsrB3 OsMsrB5 PtMsrA2.1 PtMsrA2.2 PtMsrA4.1 PtMsrA4.2 PtMsrA5 PtMsrB1 PtMsrB3.1 PtMsrB3.2 PtMsrB5	1	Target of early activation tagged 1 (RAP2.7) (tertiary DNA binding preference)
AtMsrA5 AtMsrB1 AtMsrB2 AtMsrB3 AtMsrB4 AtMsrB5 AtMsrB6 AtMsrB7 AtMsrB8 AtMsrB9 AtPMSR1 AtPMSR2 AtPMSR4 OsMsrA2.1 OsMsrB5 PtMsrA2.1 PtMsrA2.2 PtMsrA4.1 PtMsrA4.2 PtMsrA5 PtMsrB1 PtMsrB3.1 PtMsrB3.2	1	Yabby transcription factor CRABS CLAW
AtMsrA5 AtMsrB2 AtMsrB3 AtMsrB4 AtMsrB5 AtMsrB6 AtMsrB7 AtMsrB8 AtMsrB9 AtPMSR2 AtPMSR3 AtPMSR4 OsMsrA2.1 OsMsrA5 OsMsrB5 PtMsrA2.1 PtMsrA2.2 PtMsrA4.1 PtMsrA5 PtMsrB1 PtMsrB3.1 PtMsrB3.2 PtMsrB5	1	AS1/AS2 repressor complex binding motif II
AtMsrB1 AtMsrB3 AtMsrB4 AtMsrB5 AtMsrB6 AtMsrB7 AtMsrB8 AtMsrB9 AtPMSR1 AtPMSR2 AtPMSR3 AtPMSR4 OsMsrA2.1 OsMsrA4 OsMsrA5 OsMsrB1 PtMsrA2.1 PtMsrA2.2 PtMsrA4.1 PtMsrB1 PtMsrB3.1 PtMsrB5	1	NAC WITH TRANSMEMBRANE MOTIF 1-LIKE 6 (NTL6/NTM1)

AtMsrA5 AtMsrB1 AtMsrB2 AtMsrB5 AtMsrB6 AtMsrB7 AtMsrB8 AtMsrB9 AtPMSR1 AtPMSR2 AtPMSR3 OsMsrA4 OsMsrB1 OsMsrB3 OsMsrB5 PtMsrA2.2 PtMsrA4.1 PtMsrA4.2 PtMsrA5 PtMsrB1 PtMsrB3.1 PtMsrB3.2	1	Storekeeper (STK), plant specific DNA binding protein important for tuber-specific and sucrose-inducible gene expression
AtMsrA5 AtMsrB1 AtMsrB3 AtMsrB4 AtMsrB6 AtMsrB7 AtMsrB8 AtMsrB9 AtPMSR1 AtPMSR2 AtPMSR3 OsMsrA2.1 OsMsrB3 OsMsrB5 PtMsrA2.1 PtMsrA2.2 PtMsrA4.1 PtMsrA4.2 PtMsrA5 PtMsrB3.1 PtMsrB3.2 PtMsrB5	1	Cellular and viral TATA box elements
AtMsrB1 AtMsrB2 AtMsrB3 AtMsrB4 AtMsrB5 AtMsrB6 AtMsrB7 AtMsrB8 AtMsrB9 AtPMSR1 AtPMSR2 AtPMSR3 OsMsrA4 OsMsrB1 PtMsrA2.2 PtMsrA4.1 PtMsrA4.2 PtMsrA5 PtMsrB1 PtMsrB3.1 PtMsrB3.2 PtMsrB5	1	GAAA motif involved in pollen specific transcriptional activation
AtMsrB1 AtMsrB2 AtMsrB3 AtMsrB4 AtMsrB5 AtMsrB6 AtMsrB7 AtMsrB9 AtPMSR1 AtPMSR2 AtPMSR3 OsMsrA2.1 OsMsrA4 PtMsrA2.1 PtMsrA2.2 PtMsrA4.1 PtMsrA4.2 PtMsrA5 PtMsrB1 PtMsrB3.1 PtMsrB3.2 PtMsrB5	1	Sequence motif from the promoters of different sugar-responsive genes
AtMsrB2 AtMsrB3 AtMsrB4 AtMsrB5 AtMsrB6 AtMsrB8 AtMsrB9 AtPMSR1 AtPMSR2 AtPMSR3 OsMsrA2.1 OsMsrA4 OsMsrB5 PtMsrA2.1 PtMsrA2.2 PtMsrA4.1 PtMsrA4.2 PtMsrA5 PtMsrB1 PtMsrB3.1 PtMsrB3.2 PtMsrB5	1	Homeobox protein 34
AtMsrB1 AtMsrB2 AtMsrB3 AtMsrB4 AtMsrB5 AtMsrB6 AtMsrB8 AtMsrB9 AtPMSR1 AtPMSR3 AtPMSR4 OsMsrA2.1 OsMsrA5 OsMsrB3 PtMsrA2.1 PtMsrA2.2 PtMsrA4.1 PtMsrA4.2 PtMsrA5 PtMsrB1 PtMsrB3.1 PtMsrB5	1	Prolamin box, conserved in cereal seed storage protein gene promoters
AtMsrB1 AtMsrB2 AtMsrB3 AtMsrB5 AtMsrB6 AtMsrB8 AtMsrB9 AtPMSR2 AtPMSR3 AtPMSR4 OsMsrA2.1 OsMsrA2.2 OsMsrA4 OsMsrB1 OsMsrB3 PtMsrA2.1 PtMsrA2.2 PtMsrA4.1 PtMsrA4.2 PtMsrB1 PtMsrB3.2 PtMsrB5	1	bZIP protein G-Box binding factor 1
AtMsrB2 AtMsrB3 AtMsrB4 AtMsrB5 AtMsrB6 AtMsrB7 AtMsrB8 AtMsrB9 AtPMSR1 AtPMSR2 AtPMSR3 AtPMSR4 OsMsrA4 OsMsrA5 OsMsrB5 PtMsrA2.1 PtMsrA2.2 PtMsrA4.2 PtMsrA5 PtMsrB1 PtMsrB3.2	1	Circadian clock associated 1
AtMsrA5 AtMsrB1 AtMsrB2 AtMsrB3 AtMsrB4 AtMsrB5 AtMsrB6 AtMsrB8 AtMsrB9 AtPMSR1 AtPMSR2 AtPMSR3 OsMsrA2.1 OsMsrA5 OsMsrB5 PtMsrA2.1 PtMsrA2.2 PtMsrA4.1 PtMsrA5 PtMsrB1 PtMsrB5	1	Homeobox-leucine zipper protein REVOLUTA (REV, IFL1)
AtMsrA5 AtMsrB1 AtMsrB3 AtMsrB5 AtMsrB6 AtMsrB7 AtMsrB8 AtMsrB9 AtPMSR1 AtPMSR2 AtPMSR3 OsMsrA2.1 OsMsrA5 OsMsrB5 PtMsrA2.1 PtMsrA2.2 PtMsrA4.2 PtMsrA5 PtMsrB1 PtMsrB3.2 PtMsrB5	1	Mammalian C-type LTR TATA box
AtMsrA5 AtMsrB1 AtMsrB2 AtMsrB3 AtMsrB6 AtMsrB7 AtMsrB8 AtMsrB9 AtPMSR1 AtPMSR2 AtPMSR4 OsMsrB1 PtMsrA2.1 PtMsrA2.2 PtMsrA4.1 PtMsrA4.2 PtMsrA5 PtMsrB1 PtMsrB3.1 PtMsrB3.2 PtMsrB5	1	AT-hook motif nuclear-localized protein 20
AtMsrA5 AtMsrB1 AtMsrB4 AtMsrB5 AtMsrB6 AtMsrB7 AtMsrB8 AtMsrB9 AtPMSR1 AtPMSR2 AtPMSR4 OsMsrA4 OsMsrB3 OsMsrB5 PtMsrA2.1 PtMsrA2.2 PtMsrA4.1 PtMsrA4.2 PtMsrA5 PtMsrB1 PtMsrB5	1	MADS-box protein SQUAMOSA

AtMsrA5 AtMsrB1 AtMsrB2 AtMsrB3 AtMsrB4 AtMsrB6 AtMsrB7 AtMsrB8 AtPMSR1 AtPMSR3 AtPMSR4 OsMsrA2.1 OsMsrA4 OsMsrA5 OsMsrB3 OsMsrB5 PtMsrA2.1 PtMsrA2.2 PtMsrA5 PtMsrB1 PtMsrB3.1	1	GA-regulated myb gene from barley
AtMsrA5 AtMsrB1 AtMsrB2 AtMsrB3 AtMsrB4 AtMsrB5 AtMsrB6 AtMsrB7 AtMsrB8 AtMsrB9 AtPMSR2 AtPMSR3 AtPMSR4 OsMsrA4 OsMsrA5 OsMsrB5 PtMsrA5 PtMsrB1 PtMsrB3.1 PtMsrB3.2 PtMsrB5	1	GT2-box and GT3-box motifs
AtMsrA5 AtMsrB1 AtMsrB2 AtMsrB4 AtMsrB6 AtMsrB7 AtMsrB8 AtMsrB9 AtPMSR2 AtPMSR3 AtPMSR4 OsMsrA2.1 PtMsrA2.1 PtMsrA2.2 PtMsrA4.1 PtMsrA4.2 PtMsrA5 PtMsrB1 PtMsrB3.1 PtMsrB3.2 PtMsrB5	1	WRKY DNA-binding protein 70
AtMsrA5 AtMsrB1 AtMsrB3 AtMsrB5 AtMsrB6 AtMsrB7 AtMsrB8 AtMsrB9 AtPMSR2 AtPMSR3 AtPMSR4 OsMsrA4 OsMsrB5 PtMsrA2.1 PtMsrA2.2 PtMsrA4.1 PtMsrA4.2 PtMsrA5 PtMsrB3.1 PtMsrB3.2 PtMsrB5	1	Homeobox-leucine zipper protein ATHB-24
AtMsrA5 AtMsrB2 AtMsrB3 AtMsrB4 AtMsrB5 AtMsrB6 AtMsrB7 AtMsrB8 AtMsrB9 AtPMSR1 AtPMSR2 AtPMSR3 AtPMSR4 OsMsrA2.2 OsMsrB1 PtMsrA2.1 PtMsrA2.2 PtMsrA4.1 PtMsrB1 PtMsrB3.1	1	Heat shock element
AtMsrA5 AtMsrB2 AtMsrB5 AtMsrB6 AtMsrB7 AtMsrB8 AtMsrB9 AtPMSR1 AtPMSR2 AtPMSR3 AtPMSR4 OsMsrB1 OsMsrB3 PtMsrA4.1 PtMsrA4.2 PtMsrA5 PtMsrB1 PtMsrB3.1 PtMsrB3.2 PtMsrB5	1	TATA-binding protein, general transcription factor that interacts with other factors to form the preinitiation complex at promoters
AtMsrA5 AtMsrB1 AtMsrB2 AtMsrB3 AtMsrB4 AtMsrB8 AtPMSR1 AtPMSR2 AtPMSR3 OsMsrA4 OsMsrA5 OsMsrB1 PtMsrA2.1 PtMsrA2.2 PtMsrA4.1 PtMsrA4.2 PtMsrB1 PtMsrB3.1 PtMsrB3.2 PtMsrB5	1	CCAAT-box in plant promoters
AtMsrB2 AtMsrB3 AtMsrB4 AtMsrB5 AtMsrB6 AtMsrB8 AtMsrB9 AtPMSR1 AtPMSR3 AtPMSR4 OsMsrA2.1 OsMsrA4 OsMsrB1 PtMsrA2.1 PtMsrA2.2 PtMsrA4.1 PtMsrA5 PtMsrB1 PtMsrB3.1 PtMsrB3.2	1	APETALA2
AtMsrA5 AtMsrB1 AtMsrB2 AtMsrB3 AtMsrB5 AtMsrB6 AtMsrB8 AtMsrB9 AtPMSR1 AtPMSR3 OsMsrB1 OsMsrB5 PtMsrA2.1 PtMsrA2.2 PtMsrA4.1 PtMsrA4.2 PtMsrA5 PtMsrB1 PtMsrB3.1 PtMsrB5	1	Lentivirus LTR TATA box
AtMsrB1 AtMsrB2 AtMsrB3 AtMsrB4 AtMsrB5 AtMsrB6 AtMsrB7 AtMsrB9 AtPMSR2 AtPMSR3 AtPMSR4 OsMsrA2.1 OsMsrA4 PtMsrA2.1 PtMsrA4.1 PtMsrA4.2 PtMsrA5 PtMsrB1 PtMsrB3.1 PtMsrB3.2	1	Circadian clock associated 1 (secondary DNA binding preference)
AtMsrB1 AtMsrB2 AtMsrB3 AtMsrB4 AtMsrB5 AtMsrB6 AtMsrB8 AtMsrB9 AtPMSR2 OsMsrA2.1 OsMsrA5 OsMsrB1 OsMsrB3 OsMsrB5 PtMsrA2.1 PtMsrA2.2 PtMsrA4.1 PtMsrA4.2 PtMsrB1 PtMsrB3.2	1	LATE ELONGATED HYPOCOTYL
AtMsrB1 AtMsrB5 AtMsrB6 AtMsrB7 AtMsrB8 AtMsrB9 AtPMSR1 AtPMSR2 AtPMSR3 AtPMSR4 OsMsrA2.1 OsMsrA4 PtMsrA2.1 PtMsrA2.2 PtMsrA4.2 PtMsrA5 PtMsrB3.1 PtMsrB3.2 PtMsrB5	1	Zinc finger of Arabidopsis thaliana 6 (Cold induced zinc finger protein 2)

AtMsrB1 AtMsrB3 AtMsrB4 AtMsrB5 AtMsrB6 AtMsrB7 AtMsrB8 AtMsrB9 AtPMSR1 AtPMSR3 AtPMSR4 OsMsrA5 OsMsrB1 OsMsrB3 OsMsrB5 PtMsrA4.1 PtMsrB1 PtMsrB3.1 PtMsrB3.2	1	Trihelix DNA-binding factor GT-3a
AtMsrB1 AtMsrB2 AtMsrB3 AtMsrB5 AtMsrB6 AtMsrB8 AtMsrB9 AtPMSR2 AtPMSR3 AtPMSR4 OsMsrA2.1 OsMsrA5 PtMsrA2.1 PtMsrA2.2 PtMsrA4.2 PtMsrA5 PtMsrB1 PtMsrB3.2 PtMsrB5	1	WUSCHEL-related homeobox 13
AtMsrA5 AtMsrB1 AtMsrB4 AtMsrB5 AtMsrB6 AtMsrB7 AtPMSR2 AtPMSR3 OsMsrA2.1 OsMsrA5 OsMsrB1 OsMsrB3 PtMsrA2.1 PtMsrA2.2 PtMsrA4.1 PtMsrA4.2 PtMsrB1 PtMsrB3.1 PtMsrB5	1	Drosophila initiator motifs
AtMsrB1 AtMsrB2 AtMsrB5 AtMsrB6 AtMsrB7 AtMsrB8 AtMsrB9 AtPMSR1 AtPMSR2 AtPMSR3 OsMsrA5 OsMsrB3 OsMsrB5 PtMsrA2.1 PtMsrA2.2 PtMsrA4.1 PtMsrB3.1 PtMsrB5	1	S1F, site 1 binding factor of spinach rps1 promoter
AtMsrB1 AtMsrB6 AtMsrB8 AtMsrB9 AtPMSR1 AtPMSR2 AtPMSR4 OsMsrA4 OsMsrB1 OsMsrB5 PtMsrA2.1 PtMsrA2.2 PtMsrA4.1 PtMsrA4.2 PtMsrA5 PtMsrB3.1 PtMsrB3.2 PtMsrB5	1	TESMIN/TSO1-like CXC 6 (At2G20110)
AtMsrB1 AtMsrB2 AtMsrB3 AtMsrB6 AtMsrB7 AtMsrB8 AtMsrB9 AtPMSR1 AtPMSR2 OsMsrA2.2 OsMsrA4 OsMsrA5 OsMsrB1 OsMsrB3 PtMsrA2.1 PtMsrA5 PtMsrB1 PtMsrB5	1	Cis-element involved in SA (salicylic acid) induction of secretion-related genes via NPR1
AtMsrB3 AtMsrB4 AtMsrB5 AtMsrB6 AtMsrB7 AtMsrB8 AtMsrB9 AtPMSR1 AtPMSR2 OsMsrA2.1 OsMsrA4 PtMsrA2.1 PtMsrA2.2 PtMsrA4.1 PtMsrA4.2 PtMsrA5 PtMsrB3.2 PtMsrB5	1	Secondary wall NAC binding elements
AtMsrA5 AtMsrB1 AtMsrB2 AtMsrB9 AtPMSR1 AtPMSR3 AtPMSR4 OsMsrA2.1 OsMsrA4 OsMsrA5 OsMsrB3 OsMsrB5 PtMsrA2.1 PtMsrA4.1 PtMsrB1 PtMsrB3.1 PtMsrB3.2 PtMsrB5	1	Myb domain protein r1 (ATMYB44)
AtMsrB1 AtMsrB2 AtMsrB3 AtMsrB5 AtMsrB8 AtPMSR1 AtPMSR3 AtPMSR4 OsMsrA2.2 OsMsrA4 OsMsrA5 OsMsrB1 OsMsrB3 OsMsrB5 PtMsrA4.2 PtMsrB1 PtMsrB3.1 PtMsrB3.2	1	GAZ-like 3 (AT5G22990)
AtMsrA5 AtMsrB2 AtMsrB5 AtMsrB6 AtMsrB7 AtPMSR1 AtPMSR3 OsMsrA2.1 OsMsrA4 OsMsrB3 OsMsrB5 PtMsrA2.1 PtMsrA4.1 PtMsrA4.2 PtMsrA5 PtMsrB1 PtMsrB3.1 PtMsrB3.2	1	KH and zinc finger CCCH domain-containing protein
AtMsrB1 AtMsrB2 AtMsrB4 AtMsrB5 AtMsrB6 AtMsrB8 AtMsrB9 AtPMSR1 AtPMSR3 OsMsrB3 PtMsrA2.1 PtMsrA2.2 PtMsrA4.1 PtMsrA4.2 PtMsrA5 PtMsrB3.1 PtMsrB3.2 PtMsrB5	1	Soybean embryo factor 4
AtMsrA5 AtMsrB1 AtMsrB2 AtMsrB3 AtMsrB4 AtMsrB5 AtMsrB6 AtMsrB8 AtMsrB9 AtPMSR1 AtPMSR4 OsMsrA2.1 OsMsrB1 OsMsrB3 PtMsrA2.2 PtMsrA4.1 PtMsrA5 PtMsrB5	1	Arabidopsis 6b-interacting protein 1-like 1

AtMsrB2 AtMsrB6 AtMsrB7 AtMsrB8 AtMsrB9 AtPMSR1 AtPMSR4 OsMsrA2.1 OsMsrB1 OsMsrB5 PtMsrA2.1 PtMsrA2.2 PtMsrA4.1 PtMsrA4.2 PtMsrA5 PtMsrB3.1 PtMsrB3.2 PtMsrB5	1	Evening element
AtMsrA5 AtMsrB1 AtMsrB2 AtMsrB3 AtMsrB5 AtMsrB6 AtMsrB7 AtMsrB9 AtPMSR2 AtPMSR3 OsMsrA2.1 OsMsrA4 OsMsrA5 OsMsrB5 PtMsrA5 PtMsrB3.1 PtMsrB3.2 PtMsrB5	1	Dof3 - single zinc finger transcription factor
AtMsrA5 AtMsrB1 AtMsrB2 AtMsrB3 AtMsrB6 AtMsrB8 AtMsrB9 AtPMSR2 AtPMSR3 OsMsrB1 OsMsrB3 OsMsrB5 PtMsrA2.1 PtMsrA4.2 PtMsrA5 PtMsrB1 PtMsrB3.2 PtMsrB5	1	Zea mays MYB-related protein 1 (transfer cell specific)
AtMsrA5 AtMsrB5 AtMsrB6 AtMsrB7 AtMsrB8 AtMsrB9 AtPMSR3 OsMsrA2.1 OsMsrA4 PtMsrA2.1 PtMsrA2.2 PtMsrA4.1 PtMsrA4.2 PtMsrA5 PtMsrB1 PtMsrB3.1 PtMsrB3.2 PtMsrB5	1	Homeobox-leucine zipper protein ATHB-23
AtMsrB1 AtMsrB3 AtMsrB4 AtMsrB7 AtPMSR1 AtPMSR2 AtPMSR3 AtPMSR4 OsMsrA2.1 OsMsrA2.2 OsMsrA5 OsMsrB1 OsMsrB3 PtMsrA2.1 PtMsrA2.2 PtMsrB3.1 PtMsrB3.2	1	Type-B response regulator (ARR10), member of the GARP-family of plant myb-related DNA binding motifs
AtMsrB1 AtMsrB2 AtMsrB3 AtMsrB4 AtMsrB8 AtMsrB9 AtPMSR1 AtPMSR2 AtPMSR3 OsMsrA2.1 OsMsrA2.2 OsMsrA4 OsMsrA5 OsMsrB3 PtMsrA2.1 PtMsrB3.1 PtMsrB3.2	1	Myb-related protein 3R-1 (PC-MYB1)
AtMsrB4 AtMsrB5 AtMsrB6 AtMsrB7 AtMsrB8 AtMsrB9 AtPMSR1 AtPMSR2 AtPMSR3 OsMsrA2.1 OsMsrA4 PtMsrA2.2 PtMsrA4.1 PtMsrA4.2 PtMsrB1 PtMsrB3.2 PtMsrB5	1	Heat stress transcription factor B-3
AtMsrA5 AtMsrB1 AtMsrB2 AtMsrB3 AtMsrB4 AtMsrB5 AtMsrB6 AtMsrB7 AtMsrB8 AtMsrB9 AtPMSR1 AtPMSR2 OsMsrA2.1 OsMsrA4 OsMsrA5 PtMsrA4.2 PtMsrA5	1	Maize INDETERMINATE1 zinc finger protein
AtMsrA5 AtMsrB3 AtMsrB4 AtMsrB6 AtMsrB8 AtPMSR1 AtPMSR3 AtPMSR4 OsMsrA2.1 OsMsrA4 OsMsrA5 OsMsrB1 PtMsrA2.1 PtMsrA2.2 PtMsrA5 PtMsrB3.1 PtMsrB5	1	Calmodulin-binding NAC protein
AtMsrB1 AtMsrB4 AtMsrB5 AtMsrB6 AtMsrB7 AtMsrB8 AtMsrB9 AtPMSR1 AtPMSR3 AtPMSR4 OsMsrA2.1 OsMsrA5 OsMsrB5 PtMsrA4.1 PtMsrB1 PtMsrB3.1 PtMsrB3.2	1	Myb domain protein 98
AtMsrA5 AtMsrB1 AtMsrB2 AtMsrB3 AtMsrB4 AtMsrB5 AtMsrB7 AtMsrB9 AtPMSR1 AtPMSR4 OsMsrA2.1 OsMsrB1 OsMsrB3 PtMsrA2.1 PtMsrB1 PtMsrB3.1 PtMsrB5	1	NAC with transmembrane mtif 1-like 8 (NTL8/NTM1-like 8)
AtMsrB1 AtMsrB3 AtMsrB4 AtMsrB5 AtMsrB7 AtMsrB8 AtPMSR1 OsMsrA2.1 OsMsrA4 OsMsrA5 OsMsrB1 OsMsrB3 OsMsrB5 PtMsrA2.1 PtMsrA4.2 PtMsrB3.2 PtMsrB5	1	Squamosa promoter-binding-like protein 9

AtMsrA5 AtMsrB1 AtMsrB2 AtMsrB4 AtMsrB6 AtMsrB7 AtMsrB8 AtPMSR2 AtPMSR3 AtPMSR4 OsMsrA5 OsMsrB1 PtMsrA2.1 PtMsrA4.1 PtMsrB3.1 PtMsrB3.2 PtMsrB5	1	Motif similar to the conserved 8 bp distal element of the 43 bp pseudo-palindromic nitrogen response element (NRE)
AtMsrA5 AtMsrB1 AtMsrB5 AtMsrB6 AtMsrB8 AtPMSR2 AtPMSR3 AtPMSR4 OsMsrA4 OsMsrB1 PtMsrA2.1 PtMsrA2.2 PtMsrA4.2 PtMsrB1 PtMsrB3.1 PtMsrB3.2 PtMsrB5	1	Myb domain protein 107
AtMsrB1 AtMsrB2 AtMsrB4 AtMsrB5 AtMsrB6 AtMsrB9 AtPMSR2 AtPMSR3 AtPMSR4 OsMsrA4 OsMsrA5 PtMsrA2.1 PtMsrA2.2 PtMsrA4.1 PtMsrA4.2 PtMsrA5 PtMsrB3.2	1	Telomere repeat-binding protein 5
AtMsrA5 AtMsrB1 AtMsrB3 AtMsrB4 AtMsrB5 AtMsrB6 AtMsrB7 AtPMSR2 AtPMSR3 OsMsrA2.1 OsMsrA5 OsMsrB5 PtMsrA2.1 PtMsrA4.2 PtMsrB1 PtMsrB3.1 PtMsrB5	1	AGL15, Arabidopsis MADS-domain protein AGAMOUS-like 15
AtMsrA5 AtMsrB1 AtMsrB2 AtMsrB3 AtMsrB4 AtMsrB5 AtMsrB6 AtMsrB9 AtPMSR2 OsMsrA4 OsMsrB1 OsMsrB3 OsMsrB5 PtMsrA5 PtMsrB1 PtMsrB3.1 PtMsrB3.2	1	PBF (MPBF)
AtMsrA5 AtMsrB1 AtMsrB2 AtMsrB3 AtMsrB4 AtMsrB6 AtMsrB8 AtPMSR2 OsMsrA2.1 OsMsrA5 OsMsrB1 OsMsrB3 PtMsrA2.2 PtMsrA4.2 PtMsrA5 PtMsrB1 PtMsrB3.1	1	NAC domain containing protein 103
AtMsrB1 AtMsrB4 AtMsrB5 AtMsrB6 AtMsrB7 AtMsrB8 AtPMSR2 OsMsrA2.1 OsMsrA4 OsMsrB3 OsMsrB5 PtMsrA2.1 PtMsrA2.2 PtMsrA4.1 PtMsrB3.1 PtMsrB3.2 PtMsrB5	1	DNA-binding protein of sweet potato that binds to the SP8a (ACTGTGTA) and SP8b (TACTATT) sequences of sporamin and beta-amylase genes
AtMsrB1 AtMsrB2 AtMsrB4 AtMsrB6 AtMsrB7 AtMsrB8 AtMsrB9 AtPMSR3 AtPMSR4 OsMsrA2.1 OsMsrA5 OsMsrB5 PtMsrA2.2 PtMsrA4.1 PtMsrA4.2 PtMsrB1 PtMsrB5	1	Early Flowering MYB Protein (AT2G03500)
AtMsrA5 AtMsrB2 AtMsrB4 AtMsrB5 AtMsrB6 AtMsrB8 AtMsrB9 AtPMSR1 AtPMSR2 AtPMSR4 OsMsrA2.1 OsMsrB1 OsMsrB3 PtMsrA2.2 PtMsrA5 PtMsrB3.1	1	ICE (inducer of CBF expression 1), AtMYC2 (rd22BP1)
AtMsrB2 AtMsrB3 AtMsrB4 AtMsrB6 AtMsrB7 AtPMSR1 AtPMSR2 AtPMSR4 OsMsrA2.1 OsMsrA5 OsMsrB5 PtMsrA2.1 PtMsrA2.2 PtMsrA4.2 PtMsrB1 PtMsrB5	1	Phosphate starvation response 1
AtMsrA5 AtMsrB1 AtMsrB3 AtMsrB5 AtMsrB6 AtMsrB7 AtMsrB8 AtMsrB9 AtPMSR1 AtPMSR2 OsMsrA4 OsMsrA5 PtMsrA2.1 PtMsrA2.2 PtMsrA5 PtMsrB5	1	Homeobox-leucine zipper protein ATHB-6
AtMsrA5 AtMsrB3 AtMsrB4 AtMsrB6 AtMsrB7 AtMsrB8 AtMsrB9 AtPMSR1 AtPMSR2 OsMsrA2.1 OsMsrA4 PtMsrA2.1 PtMsrA2.2 PtMsrA5 PtMsrB1 PtMsrB3.2	1	NAC domain containing protein 5

AtMsrA5 AtMsrB1 AtMsrB3 AtMsrB4 AtMsrB7 AtMsrB9 AtPMSR1 AtPMSR3 AtPMSR4 OsMsrB1 OsMsrB3 PtMsrA2.1 PtMsrA2.2 PtMsrA4.1 PtMsrA4.2 PtMsrA5	1	Dof1 / MNB1a - single zinc finger transcription factor
AtMsrB3 AtMsrB6 AtMsrB7 AtMsrB8 AtMsrB9 AtPMSR1 AtPMSR3 AtPMSR4 OsMsrA2.1 OsMsrA4 OsMsrB5 PtMsrA2.1 PtMsrA5 PtMsrB1 PtMsrB3.1 PtMsrB5	1	Botrytis-susceptible1 (MYB108)
AtMsrB4 AtMsrB5 AtMsrB6 AtMsrB7 AtMsrB8 AtPMSR1 AtPMSR3 AtPMSR4 OsMsrA4 OsMsrA5 OsMsrB1 OsMsrB5 PtMsrA2.1 PtMsrA4.2 PtMsrB1 PtMsrB5	1	Myb domain protein 3R-4
AtMsrA5 AtMsrB5 AtMsrB6 AtMsrB7 AtMsrB8 AtMsrB9 AtPMSR1 OsMsrB1 PtMsrA2.1 PtMsrA2.2 PtMsrA4.1 PtMsrA4.2 PtMsrA5 PtMsrB1 PtMsrB3.1 PtMsrB3.2	1	High mobility group I/Y-like proteins
AtMsrA5 AtMsrB1 AtMsrB2 AtMsrB5 AtMsrB8 AtPMSR2 AtPMSR3 AtPMSR4 OsMsrA2.1 OsMsrA2.2 OsMsrA4 OsMsrB1 PtMsrA4.1 PtMsrA4.2 PtMsrB3.2 PtMsrB5	1	Transcription factor of rice and barley binding to the iron deficiency-responsive cis-acting element 2 (IDE2)
AtMsrA5 AtMsrB2 AtMsrB3 AtMsrB7 AtMsrB8 AtMsrB9 AtPMSR2 AtPMSR3 AtPMSR4 OsMsrA2.2 OsMsrB3 PtMsrA2.1 PtMsrA4.2 PtMsrA5 PtMsrB3.1 PtMsrB5	1	NIN-binding nucleotide sequences
AtMsrA5 AtMsrB2 AtMsrB7 AtMsrB8 AtPMSR2 AtPMSR3 AtPMSR4 OsMsrA5 OsMsrB1 OsMsrB5 PtMsrA2.1 PtMsrA2.2 PtMsrA4.2 PtMsrA5 PtMsrB3.2 PtMsrB5	1	Zinc-dependent activator protein-1, WRKY 1
AtMsrA5 AtMsrB1 AtMsrB2 AtMsrB3 AtMsrB9 AtPMSR2 AtPMSR3 OsMsrA2.1 OsMsrA4 PtMsrA2.1 PtMsrA2.2 PtMsrA4.1 PtMsrA5 PtMsrB1 PtMsrB3.1 PtMsrB5	1	WRKY DNA-binding protein 50
AtMsrB1 AtMsrB2 AtMsrB4 AtMsrB5 AtMsrB6 AtMsrB7 AtMsrB9 AtPMSR2 AtPMSR3 OsMsrA2.1 OsMsrA4 PtMsrA2.1 PtMsrA2.2 PtMsrA4.1 PtMsrA5 PtMsrB3.1	1	NAC with transmembrane motif 1
AtMsrA5 AtMsrB1 AtMsrB5 AtMsrB7 AtPMSR2 AtPMSR4 OsMsrA2.1 OsMsrA2.2 OsMsrA4 OsMsrA5 OsMsrB5 PtMsrA2.1 PtMsrA4.1 PtMsrA4.2 PtMsrA5 PtMsrB3.2	1	Opaque-2 regulatory protein
AtMsrA5 AtMsrB1 AtMsrB2 AtMsrB6 AtMsrB8 AtMsrB9 AtPMSR2 OsMsrA2.1 OsMsrA4 PtMsrA2.1 PtMsrA2.2 PtMsrA4.1 PtMsrA4.2 PtMsrB1 PtMsrB3.1 PtMsrB5	1	Trihelix transcription factor GTL1
AtMsrA5 AtMsrB2 AtMsrB3 AtMsrB4 AtMsrB5 AtMsrB7 AtMsrB9 AtPMSR2 OsMsrA4 OsMsrB5 PtMsrA2.2 PtMsrA4.1 PtMsrA4.2 PtMsrA5 PtMsrB1 PtMsrB5	1	Muscle TATA box
AtMsrB2 AtMsrB3 AtMsrB5 AtMsrB6 AtMsrB7 AtPMSR3 OsMsrA4 OsMsrA5 OsMsrB5 PtMsrA2.1 PtMsrA2.2 PtMsrA4.1 PtMsrA5 PtMsrB1 PtMsrB3.1 PtMsrB3.2	1	Myb family transcription factor At3g10113

AtMsrA5 AtMsrB1 AtMsrB2 AtMsrB3 AtMsrB4 AtMsrB5 AtMsrB6 AtMsrB7 AtMsrB9 OsMsrA2.1 OsMsrA5 OsMsrB1 OsMsrB3 PtMsrA2.1 PtMsrB3.2 PtMsrB5	1	Recognition site for BZIP transcription factors that belong to the group of Opaque-2 like proteins
AtMsrA5 AtMsrB6 AtMsrB7 AtMsrB9 AtPMSR1 AtPMSR2 AtPMSR3 AtPMSR4 OsMsrB1 PtMsrA2.1 PtMsrA2.2 PtMsrA4.2 PtMsrA5 PtMsrB3.1 PtMsrB5	1	Secondary wall MYB-responsive element, MYB46 and MYB83 binding sites
AtMsrB2 AtMsrB3 AtMsrB5 AtMsrB7 AtMsrB9 AtPMSR1 AtPMSR2 AtPMSR3 AtPMSR4 OsMsrA2.1 OsMsrB1 OsMsrB3 PtMsrA4.2 PtMsrA5 PtMsrB1	1	WRKY plant specific zinc-finger-type factor associated with pathogen defence, W box
AtMsrA5 AtMsrB1 AtMsrB2 AtMsrB3 AtMsrB5 AtMsrB6 AtMsrB9 AtPMSR1 AtPMSR2 AtPMSR4 OsMsrA5 PtMsrA2.2 PtMsrA4.1 PtMsrA4.2 PtMsrB1	1	Myb domain protein 52
AtMsrA5 AtMsrB3 AtMsrB4 AtMsrB6 AtMsrB7 AtMsrB9 AtPMSR1 AtPMSR2 AtPMSR4 OsMsrA4 OsMsrA5 OsMsrB5 PtMsrA2.1 PtMsrA4.2 PtMsrB5	1	B3 domain-containing transcription factor FUS3
AtMsrB1 AtMsrB3 AtMsrB4 AtMsrB5 AtMsrB7 AtMsrB8 AtMsrB9 AtPMSR1 AtPMSR2 OsMsrA2.2 OsMsrA5 OsMsrB3 PtMsrA5 PtMsrB1 PtMsrB5	1	RWP-RK domain containing 2
AtMsrB2 AtMsrB4 AtMsrB5 AtMsrB6 AtMsrB7 AtMsrB9 AtPMSR1 AtPMSR2 OsMsrA2.1 OsMsrA2.2 OsMsrA5 OsMsrB3 PtMsrA2.2 PtMsrB1 PtMsrB5	1	Growth-regulating factor 9
AtMsrA5 AtMsrB1 AtMsrB2 AtMsrB7 AtMsrB8 AtMsrB9 AtPMSR1 AtPMSR3 AtPMSR4 OsMsrA5 OsMsrB3 OsMsrB5 PtMsrA2.1 PtMsrA2.2 PtMsrA4.1	1	NAC domain containing protein 3
AtMsrA5 AtMsrB1 AtMsrB2 AtMsrB3 AtMsrB6 AtMsrB7 AtMsrB8 AtMsrB9 AtPMSR1 AtPMSR4 OsMsrA2.2 OsMsrA5 OsMsrB1 PtMsrA2.2 PtMsrA4.2	1	Nodulin consensus sequence 2
AtMsrA5 AtMsrB2 AtMsrB3 AtMsrB5 AtMsrB7 AtPMSR2 AtPMSR3 OsMsrA2.1 PtMsrA2.1 PtMsrA2.2 PtMsrA4.2 PtMsrA5 PtMsrB1 PtMsrB3.1 PtMsrB5	1	GT1-Box binding factors with a trihelix DNA-binding domain
AtMsrB2 AtMsrB6 AtMsrB7 AtMsrB9 AtPMSR2 AtPMSR3 OsMsrA2.1 OsMsrA2.2 OsMsrA4 OsMsrA5 OsMsrB1 OsMsrB3 OsMsrB5 PtMsrA4.2 PtMsrB1	1	Brassinazole-resistant 1
AtMsrB1 AtMsrB2 AtMsrB4 AtMsrB6 AtMsrB7 AtMsrB9 AtPMSR2 AtPMSR4 OsMsrA2.2 OsMsrA5 PtMsrA2.1 PtMsrA5 PtMsrB1 PtMsrB3.1 PtMsrB5	1	Hormone up-regulated at dawn element
AtMsrB5 AtMsrB6 AtMsrB7 AtMsrB9 AtPMSR4 OsMsrA2.2 OsMsrA4 OsMsrA5 OsMsrB1 PtMsrA4.1 PtMsrA4.2 PtMsrB1 PtMsrB3.1 PtMsrB3.2 PtMsrB5	1	Arabidopsis NAC domain containing protein 19
AtMsrB3 AtMsrB4 AtMsrB5 AtMsrB6 AtMsrB7 AtPMSR1 AtPMSR2 AtPMSR3 AtPMSR4 OsMsrB3 OsMsrB5 PtMsrA2.1 PtMsrA5 PtMsrB1	1	Tracheary-element-regulating cis-element
AtMsrB6 AtMsrB7 AtMsrB9 AtPMSR1 AtPMSR2 AtPMSR3 AtPMSR4 OsMsrA5 OsMsrB1 OsMsrB5 PtMsrA2.1 PtMsrA4.1 PtMsrB3.1 PtMsrB3.2	1	Myb domain protein 49
AtMsrB1 AtMsrB2 AtMsrB4 AtMsrB5 AtMsrB6 AtMsrB7 AtMsrB8 AtPMSR1 AtPMSR2 AtPMSR3 OsMsrA4 OsMsrA5 PtMsrA5 PtMsrB1	1	Myb family transcription factor (G2-like family)
AtMsrB1 AtMsrB2 AtMsrB3 AtMsrB8 AtMsrB9 AtPMSR1 AtPMSR2 AtPMSR4 OsMsrA2.2 OsMsrA4 PtMsrA4.2 PtMsrB1 PtMsrB3.2 PtMsrB5	1	NAC WITH TRANSMEMBRANE MOTIF 1-LIKE 8 (NTL8/NTM1-like 8)
AtMsrA5 AtMsrB1 AtMsrB3 AtMsrB4 AtMsrB6 AtMsrB7 AtMsrB8 AtMsrB9 AtPMSR1 AtPMSR2 OsMsrA2.1 OsMsrB1 PtMsrA2.1 PtMsrA5	1	NAC domain containing protein 4

AtMsrB1 AtMsrB5 AtMsrB7 AtMsrB8 AtMsrB9 AtPMSR1 AtPMSR2 PtMsrA2.1 PtMsrA2.2 PtMsrA4.1 PtMsrA4.2 PtMsrA5 PtMsrB1 PtMsrB5	1	Homeobox 51, Late Meristem Identity 1
AtMsrA5 AtMsrB2 AtMsrB3 AtMsrB4 AtMsrB5 AtMsrB7 AtPMSR1 AtPMSR3 AtPMSR4 OsMsrA2.2 OsMsrA4 OsMsrB3 PtMsrA2.1 PtMsrA2.2	1	Basic pentacysteine proteins
AtMsrA5 AtMsrB1 AtMsrB2 AtMsrB3 AtMsrB6 AtMsrB7 AtMsrB8 AtPMSR1 AtPMSR4 OsMsrA5 OsMsrB5 PtMsrA4.1 PtMsrB3.1 PtMsrB5	1	Wheat NAC-domain DNA binding factor (DNA binding site II)
AtMsrB1 AtMsrB2 AtMsrB9 AtPMSR1 AtPMSR4 OsMsrA2.2 OsMsrA5 OsMsrB1 OsMsrB3 OsMsrB5 PtMsrA2.1 PtMsrA4.2 PtMsrB3.1 PtMsrB3.2	1	Oryza sativa CaM-binding transcription factor
AtMsrA5 AtMsrB3 AtMsrB4 AtMsrB5 AtPMSR2 AtPMSR3 AtPMSR4 OsMsrA2.1 OsMsrA2.2 OsMsrA4 OsMsrA5 OsMsrB1 OsMsrB3 PtMsrB5	1	Arabidopsis 6B-interacting protein 1-like 2 (AT3G14180)
AtMsrA5 AtMsrB2 AtMsrB4 AtMsrB7 AtMsrB8 AtPMSR2 AtPMSR3 OsMsrA2.1 OsMsrA5 OsMsrB1 PtMsrA2.1 PtMsrA5 PtMsrB1 PtMsrB3.2	1	Paired amphipathic helix domain-containing protein
AtMsrB1 AtMsrB3 AtMsrB7 AtMsrB8 AtMsrB9 AtPMSR2 AtPMSR3 OsMsrB3 OsMsrB5 PtMsrA4.1 PtMsrB1 PtMsrB3.1 PtMsrB3.2 PtMsrB5	1	Protein ethylene insensitive 3
AtMsrB2 AtMsrB3 AtMsrB5 AtPMSR2 AtPMSR3 OsMsrA2.1 OsMsrA4 OsMsrA5 OsMsrB5 PtMsrA2.1 PtMsrA4.1 PtMsrA4.2 PtMsrA5 PtMsrB3.2	1	Telomere binding protein 3 (TRB2)
AtMsrA5 AtMsrB1 AtMsrB2 AtMsrB3 AtMsrB4 AtPMSR2 AtPMSR4 OsMsrA2.1 OsMsrA5 OsMsrB3 OsMsrB5 PtMsrA2.1 PtMsrA4.1 PtMsrB3.1	1	ETTIN (Auxin Response Factor 3)
AtMsrB1 AtMsrB2 AtMsrB5 AtMsrB8 AtPMSR2 AtPMSR4 OsMsrA2.1 OsMsrA2.2 PtMsrA2.1 PtMsrA4.1 PtMsrA4.2 PtMsrB1 PtMsrB3.2 PtMsrB5	1	Phytochrome interacting factor3-like 5
AtMsrA5 AtMsrB3 AtMsrB6 AtMsrB7 AtMsrB8 AtPMSR3 AtPMSR4 OsMsrA2.1 OsMsrA4 OsMsrA5 OsMsrB3 OsMsrB5 PtMsrB3.1 PtMsrB5	1	NACL-inducible gene 1
AtMsrA5 AtMsrB2 AtMsrB4 AtMsrB6 AtMsrB7 AtMsrB8 AtMsrB9 AtPMSR3 OsMsrA2.1 OsMsrA5 OsMsrB3 OsMsrB5 PtMsrA2.1 PtMsrB1	1	AP2/ERF and B3 domain-containing transcription factor RAV1
AtMsrA5 AtMsrB2 AtMsrB4 AtMsrB6 AtMsrB7 AtMsrB8 AtPMSR3 OsMsrA2.1 OsMsrA4 OsMsrB1 OsMsrB3 PtMsrA4.1 PtMsrA4.2 PtMsrB3.2	1	Morning element (sequence motifs enriched in morning-phased genes)
AtMsrB1 AtMsrB3 AtMsrB7 AtMsrB8 AtMsrB9 AtPMSR1 AtPMSR2 AtPMSR4 OsMsrA4 OsMsrA5 OsMsrB3 PtMsrB1 PtMsrB3.2	1	NAC domain containing protein 71
AtMsrA5 AtMsrB1 AtMsrB3 AtMsrB5 AtMsrB7 AtMsrB9 AtPMSR1 AtPMSR2 OsMsrB5 PtMsrA2.1 PtMsrA4.2 PtMsrA5 PtMsrB3.2	1	Homeobox-leucine zipper protein ATHB-15 (INCURVATA 4)
AtMsrA5 AtMsrB1 AtMsrB3 AtMsrB5 AtPMSR1 AtPMSR2 OsMsrA2.2 OsMsrA4 OsMsrA5 OsMsrB1 OsMsrB5 PtMsrA2.2 PtMsrB3.1	1	Shoot-apical-meristem arrest 2 (AT5G08750)
AtMsrA5 AtMsrB6 AtMsrB7 AtPMSR1 AtPMSR2 OsMsrB1 PtMsrA2.2 PtMsrA4.1 PtMsrA4.2 PtMsrB1 PtMsrB3.1 PtMsrB3.2 PtMsrB5	1	Homeobox-leucine zipper protein ATHB-5
AtMsrB2 AtMsrB4 AtMsrB6 AtMsrB9 AtPMSR1 AtPMSR2 OsMsrA2.1 OsMsrA5 OsMsrB1 OsMsrB5 PtMsrA2.2 PtMsrA4.1 PtMsrB3.2	1	Myb domain protein 99 (ATMYBCU15)
AtMsrB2 AtMsrB3 AtMsrB4 AtMsrB7 AtPMSR1 AtPMSR3 AtPMSR4 OsMsrB5 PtMsrA2.1 PtMsrA2.2 PtMsrA4.1 PtMsrA4.2 PtMsrB1	1	PHR1-like 2 (G2-like family)(AT3G24120)

AtMsrB1 AtMsrB2 AtMsrB8 AtPMSR1 AtPMSR4 OsMsrA2.1 OsMsrA2.2 OsMsrA5 OsMsrB1 PtMsrA4.1 PtMsrA4.2 PtMsrB1 PtMsrB3.2	1	Rice transcription activator-1 (RITA), basic leucin zipper protein, highly expressed during seed development
AtMsrB2 AtMsrB3 AtMsrB5 AtMsrB7 AtMsrB8 AtMsrB9 AtPMSR1 AtPMSR4 OsMsrA4 PtMsrA4.1 PtMsrA4.2 PtMsrB3.1 PtMsrB3.2	1	Homeodomain glabrous 9
AtMsrB2 AtMsrB5 AtMsrB7 AtMsrB8 AtMsrB9 AtPMSR1 AtPMSR4 OsMsrA2.1 OsMsrA4 OsMsrA5 PtMsrA2.1 PtMsrA4.1 PtMsrA4.2	1	Myb domain protein 57
AtMsrA5 AtMsrB1 AtMsrB3 AtMsrB5 AtPMSR2 AtPMSR3 AtPMSR4 OsMsrA4 PtMsrA2.1 PtMsrA4.2 PtMsrA5 PtMsrB1 PtMsrB5	1	5'-part of bipartite RAV1 binding site, interacting with AP2 domain
AtMsrA5 AtMsrB4 AtMsrB7 AtMsrB9 AtPMSR2 AtPMSR3 AtPMSR4 OsMsrA4 OsMsrB5 PtMsrA5 PtMsrB1 PtMsrB3.2 PtMsrB5	1	Arabidopsis thaliana meristem layer 1
AtMsrB2 AtMsrB3 AtMsrB6 AtMsrB7 AtMsrB9 AtPMSR2 AtPMSR3 OsMsrA5 OsMsrB5 PtMsrA4.1 PtMsrA4.2 PtMsrB1 PtMsrB5	1	KANADI 4 (Aberrant Testa Shape)
AtMsrA5 AtMsrB6 AtMsrB7 AtMsrB9 AtPMSR2 AtPMSR4 OsMsrA5 OsMsrB3 PtMsrA2.1 PtMsrA4.2 PtMsrA5 PtMsrB1 PtMsrB3.1	1	CA-rich element
AtMsrB3 AtMsrB4 AtMsrB8 AtMsrB9 AtPMSR2 AtPMSR4 OsMsrB5 PtMsrA2.2 PtMsrA4.1 PtMsrA4.2 PtMsrA5 PtMsrB1 PtMsrB3.2	1	L1-specific homeodomain protein ATML1 (A. thaliana meristem layer 1)
AtMsrA5 AtMsrB1 AtMsrB3 AtMsrB5 AtMsrB6 AtMsrB7 AtMsrB9 AtPMSR2 PtMsrA2.1 PtMsrA2.2 PtMsrA4.2 PtMsrA5 PtMsrB1	1	PHAVOLUTA (Homeobox-leucine zipper protein ATHB-9)
AtMsrA5 AtMsrB1 AtMsrB6 AtMsrB8 AtPMSR2 OsMsrA2.1 OsMsrA4 OsMsrB1 PtMsrA2.1 PtMsrA2.2 PtMsrA4.1 PtMsrA4.2 PtMsrB1	1	Binding sites for AP1, AP3-PI and AG dimers
AtMsrB1 AtMsrB3 AtMsrB4 AtMsrB5 AtMsrB6 AtMsrB7 AtMsrB9 AtPMSR2 OsMsrA2.1 OsMsrB3 PtMsrA4.1 PtMsrA4.2 PtMsrB5	1	AG-motif binding protein 1
AtMsrB7 AtMsrB8 AtMsrB9 AtPMSR3 OsMsrA2.1 OsMsrA4 OsMsrA5 OsMsrB5 PtMsrA2.1 PtMsrA2.2 PtMsrA4.1 PtMsrB1 PtMsrB3.2	1	Male sterile 188 (MYB103)
AtMsrA5 AtMsrB1 AtMsrB3 AtMsrB4 AtMsrB7 AtMsrB9 OsMsrA4 OsMsrB3 PtMsrA2.1 PtMsrA2.2 PtMsrA5 PtMsrB3.1 PtMsrB3.2	1	AGL3, MADS Box protein
AtMsrB1 AtMsrB2 AtMsrB3 AtMsrB4 AtMsrB5 AtMsrB9 OsMsrA2.1 OsMsrA5 OsMsrB1 OsMsrB5 PtMsrA5 PtMsrB1 PtMsrB3.1	1	I-Box in rbcS genes and other light regulated genes
AtMsrB1 AtMsrB2 AtMsrB4 AtMsrB6 AtMsrB7 AtPMSR1 AtPMSR2 AtPMSR3 AtPMSR4 OsMsrA2.1 OsMsrB1 PtMsrA4.1	1	Abscisic acid responsive elements-binding factor 2 (AREB-1)
AtMsrB2 AtMsrB4 AtMsrB5 AtMsrB7 AtPMSR1 AtPMSR2 AtPMSR3 AtPMSR4 OsMsrA2.2 OsMsrA5 PtMsrA4.2 PtMsrA5	1	R2R3-type myb-like transcription factor (I-type binding site)
AtMsrB2 AtMsrB4 AtMsrB7 AtMsrB8 AtMsrB9 AtPMSR1 AtPMSR2 AtPMSR3 OsMsrA4 PtMsrA2.2 PtMsrA4.1 PtMsrA4.2	1	NAC domain containing protein 87
AtMsrB4 AtMsrB5 AtMsrB6 AtPMSR1 AtPMSR2 AtPMSR4 OsMsrA2.1 OsMsrA2.2 OsMsrA5 OsMsrB1 OsMsrB3 PtMsrA5	1	bZIP transcription factor implicated in ABA induced gene expression
AtMsrA5 AtMsrB5 AtMsrB7 AtMsrB8 AtMsrB9 AtPMSR1 AtPMSR2 OsMsrA4 OsMsrB1 PtMsrA4.1 PtMsrA4.2 PtMsrB3.2	1	AS1/AS2 repressor complex binding motif I

AtMsrB1 AtMsrB2 AtPMSR1 AtPMSR2 OsMsrA2.1 OsMsrA2.2 OsMsrA4 OsMsrA5 OsMsrB1 OsMsrB3 OsMsrB5 PtMsrA4.1	1	WRINKLED 1
AtMsrB2 AtMsrB3 AtMsrB5 AtMsrB9 AtPMSR1 AtPMSR3 OsMsrA2.1 OsMsrA4 OsMsrA5 PtMsrA4.2 PtMsrA5 PtMsrB3.1	1	Calmodulin binding WRKY transcription factor 11
AtMsrB2 AtMsrB3 AtMsrB7 AtMsrB8 AtPMSR1 AtPMSR4 OsMsrA5 OsMsrB1 OsMsrB5 PtMsrA4.2 PtMsrB1 PtMsrB5	1	Ethylene-responsive transcription factor SCHLAFMUTZE
AtMsrA5 AtMsrB1 AtMsrB6 AtMsrB7 AtPMSR1 OsMsrA2.1 OsMsrA5 PtMsrA2.1 PtMsrA2.2 PtMsrA4.1 PtMsrA4.2 PtMsrB1	1	Homeobox protein 40
AtMsrA5 AtMsrB3 AtMsrB4 AtMsrB8 AtPMSR1 OsMsrA2.1 OsMsrA4 OsMsrA5 OsMsrB3 PtMsrA2.2 PtMsrB3.1 PtMsrB3.2	1	Zinc-finger protein in alfalfa roots, regulates salt tolerance
AtMsrA5 AtMsrB4 AtMsrB6 AtMsrB9 AtPMSR2 AtPMSR3 AtPMSR4 OsMsrA4 OsMsrB5 PtMsrA2.1 PtMsrA4.1 PtMsrB3.1	1	Ethylene-responsive transcription factor RAP2.1
AtMsrB2 AtMsrB4 AtMsrB8 AtPMSR2 OsMsrA2.1 OsMsrA5 OsMsrB1 OsMsrB3 PtMsrA2.1 PtMsrA4.1 PtMsrB1 PtMsrB5	1	P1BS, PHR1 binding sequences
AtMsrA5 AtMsrB2 AtMsrB4 AtMsrB6 AtMsrB9 AtPMSR3 OsMsrA2.1 OsMsrB5 PtMsrA2.1 PtMsrB1 PtMsrB3.1 PtMsrB3.2	1	Two-component response regulator ARR11
AtMsrA5 AtMsrB6 AtMsrB7 AtMsrB8 AtPMSR3 OsMsrA4 OsMsrB5 PtMsrA4.1 PtMsrA5 PtMsrB3.1 PtMsrB3.2 PtMsrB5	1	CXC domain containing TSO1-like protein 1 (TCX3)
AtMsrB4 AtMsrB6 AtMsrB9 AtPMSR4 OsMsrA2.1 OsMsrA4 OsMsrA5 OsMsrB3 PtMsrA2.1 PtMsrA4.1 PtMsrA5 PtMsrB1	1	Heat shock transcription factor C1
AtMsrA5 AtMsrB4 AtMsrB5 AtMsrB7 AtMsrB9 OsMsrA2.1 OsMsrA4 OsMsrB1 OsMsrB5 PtMsrA4.1 PtMsrA5 PtMsrB5	1	Arabidopsis NAC domain containing protein 92 (ATNAC6)
AtMsrA5 AtMsrB1 AtMsrB3 AtMsrB6 AtMsrB9 AtPMSR1 AtPMSR2 AtPMSR3 AtPMSR4 OsMsrA2.1 PtMsrB5	1	Homeobox-leucine zipper protein ATHB-7
AtMsrB9 AtPMSR1 AtPMSR2 OsMsrA2.1 OsMsrA2.2 OsMsrA4 OsMsrA5 OsMsrB1 PtMsrA5 PtMsrB1 PtMsrB3.1	1	Arabidopsis thaliana signal-responsive gene1, Ca2+/ calmodulin binding protein homolog to NtER1 (tobacco early ethylene-responsive gene)
AtMsrB2 AtMsrB5 AtMsrB7 AtMsrB8 AtMsrB9 AtPMSR1 AtPMSR3 AtPMSR4 OsMsrB5 PtMsrA2.2 PtMsrA4.1	1	NAC secondary wall thickening promoting factor 2
AtMsrB5 AtPMSR1 AtPMSR3 AtPMSR4 OsMsrA2.1 OsMsrA2.2 OsMsrA4 OsMsrB1 OsMsrB3 OsMsrB5 PtMsrB5	1	bZIP transcription factor binding to OCS-elements
AtMsrB1 AtMsrB2 AtMsrB4 AtMsrB7 AtMsrB9 AtPMSR1 AtPMSR3 OsMsrA4 OsMsrB1 PtMsrB3.2 PtMsrB5	1	Salt tolerance zinc finger (ZAT10)
AtMsrB2 AtMsrB3 AtMsrB7 AtMsrB8 AtPMSR1 AtPMSR4 OsMsrA2.1 PtMsrA4.1 PtMsrB3.1 PtMsrB3.2 PtMsrB5	1	Wheat bZIP transcription factor HBP1B (histone gene binding protein 1b)
AtMsrB3 AtMsrB5 AtMsrB7 AtPMSR1 AtPMSR4 OsMsrA2.2 OsMsrB1 OsMsrB3 PtMsrA2.1 PtMsrA5 PtMsrB5	1	Root hair-specific element with a 2-nucleotid spacer between left part (LP) and right part (RP)
AtMsrB3 AtMsrB6 AtMsrB7 AtMsrB8 AtMsrB9 AtPMSR2 AtPMSR3 OsMsrA5 PtMsrA2.1 PtMsrB1 PtMsrB5	1	Heat shock transcription factor B2A

AtMsrA5 AtMsrB3 AtMsrB7 AtPMSR2 AtPMSR4 OsMsrB1 OsMsrB5 PtMsrA4.1 PtMsrA5 PtMsrB1 PtMsrB3.2	1	Reproductive Meristem 1 (secondary DNA binding preference)
AtMsrA5 AtMsrB2 AtMsrB5 AtMsrB7 AtPMSR2 OsMsrA2.2 OsMsrA4 OsMsrB3 PtMsrA2.1 PtMsrA4.2 PtMsrB1	1	(GA)n/(CT)n binding proteins (GBP, soybean; BBR, barley)
AtMsrA5 AtMsrB5 AtMsrB9 AtPMSR2 OsMsrA2.1 OsMsrA4 OsMsrA5 OsMsrB1 OsMsrB3 OsMsrB5 PtMsrB5	1	CAACTC regulatory elements, GA-inducible
AtMsrB2 AtMsrB4 AtMsrB5 AtMsrB6 AtMsrB8 AtPMSR2 OsMsrA5 OsMsrB3 PtMsrA4.2 PtMsrB1 PtMsrB3.2	1	NAC domain containing protein 16
AtMsrB5 AtPMSR3 AtPMSR4 OsMsrA2.2 OsMsrA4 OsMsrA5 PtMsrA2.1 PtMsrA2.2 PtMsrA4.2 PtMsrB3.1 PtMsrB5	1	SEF3, Soybean embryo factor 3
AtMsrA5 AtMsrB4 AtMsrB5 AtMsrB6 AtMsrB8 AtMsrB9 AtPMSR3 OsMsrA2.2 OsMsrB5 PtMsrA4.1 PtMsrB3.1	1	Myb domain protein 65
AtMsrB1 AtMsrB3 AtMsrB4 AtMsrB5 AtMsrB6 AtPMSR3 OsMsrA2.1 OsMsrA5 OsMsrB1 PtMsrA5 PtMsrB3.1	1	LOB domain-containing protein 19
AtMsrB5 AtMsrB7 AtMsrB8 AtMsrB9 AtPMSR3 OsMsrA4 OsMsrB5 PtMsrA2.2 PtMsrA4.2 PtMsrB3.1 PtMsrB3.2	1	Homeobox protein 25
AtMsrB4 AtMsrB5 AtMsrB6 AtPMSR4 OsMsrA2.1 OsMsrA4 OsMsrA5 OsMsrB5 PtMsrA2.2 PtMsrB1 PtMsrB5	1	Squamosa promoter binding protein-like 14
AtMsrA5 AtMsrB1 AtMsrB5 AtMsrB8 PtMsrA2.1 PtMsrA2.2 PtMsrA4.1 PtMsrA4.2 PtMsrA5 PtMsrB3.1 PtMsrB3.2	1	ARID/BRIGHT DNA-binding domain-containing protein
AtMsrA5 AtMsrB2 AtMsrB6 AtMsrB7 OsMsrB5 PtMsrA2.1 PtMsrA4.1 PtMsrA4.2 PtMsrA5 PtMsrB3.1 PtMsrB3.2	1	Dof zinc finger protein DOF3.4 (OBF binding protein 1)
AtMsrB1 AtMsrB2 AtMsrB5 AtMsrB9 OsMsrA2.1 OsMsrA5 OsMsrB3 OsMsrB5 PtMsrA2.1 PtMsrB3.1 PtMsrB3.2	1	Myb domain protein 62 (BW62B)
AtMsrB1 AtMsrB2 AtMsrB6 AtMsrB8 OsMsrA2.1 PtMsrA2.1 PtMsrA2.2 PtMsrA4.2 PtMsrB1 PtMsrB3.2 PtMsrB5	1	ABA response elements
AtMsrB1 AtMsrB3 AtMsrB5 OsMsrA2.1 OsMsrA2.2 OsMsrA4 OsMsrA5 OsMsrB1 OsMsrB3 OsMsrB5 PtMsrB3.1	1	Lateral organ boundaries
AtMsrB1 AtMsrB7 AtMsrB8 OsMsrA4 OsMsrA5 OsMsrB5 PtMsrA2.1 PtMsrA5 PtMsrB1 PtMsrB3.1 PtMsrB5	1	Floral homeotic protein AGL15
AtMsrB5 AtMsrB6 AtMsrB7 AtMsrB8 OsMsrB1 PtMsrA2.2 PtMsrA4.2 PtMsrA5 PtMsrB3.1 PtMsrB3.2 PtMsrB5	1	Arabidopsis thaliana homeo box protein 1
AtMsrA5 AtMsrB2 AtMsrB8 AtPMSR1 AtPMSR2 AtPMSR4 OsMsrA2.1 OsMsrB1 PtMsrA2.1 PtMsrB3.2	1	Root hair-specific element with a 3-nucleotid spacer between left part (LP) and right part (RP)
AtMsrB1 AtMsrB2 AtMsrB7 AtPMSR1 AtPMSR2 AtPMSR4 OsMsrB3 PtMsrA2.1 PtMsrA2.2 PtMsrA4.2	1	Flowering locus C
AtMsrA5 AtMsrB5 AtMsrB6 AtPMSR1 AtPMSR2 OsMsrA5 PtMsrA2.2 PtMsrA4.1 PtMsrB1 PtMsrB3.1	1	GCN4, conserved in cereal seed storage protein gene promoters, similar to yeast GCN4 and vertebrate AP-1

AtMsrB6 AtPMSR1 AtPMSR2 OsMsrA2.1 OsMsrA2.2 OsMsrA4 OsMsrA5 OsMsrB1 OsMsrB3 PtMsrB1	1	Octamer motif found in plant histone H3 and H4 genes
AtMsrA5 AtMsrB1 AtMsrB2 AtMsrB4 AtMsrB6 AtMsrB7 AtPMSR1 AtPMSR3 OsMsrA2.1 PtMsrA2.2	1	NAC domain containing protein 79 (AT5G07680)
AtMsrB1 AtMsrB2 AtMsrB3 AtMsrB5 AtMsrB7 AtMsrB8 AtPMSR1 AtPMSR3 OsMsrB5 PtMsrA2.1	1	Myb domain protein 56
AtMsrB2 AtMsrB3 AtMsrB6 AtMsrB7 AtPMSR1 AtPMSR3 OsMsrA5 PtMsrA2.1 PtMsrA5 PtMsrB3.2	1	TEF cis acting elements in both RNA polymerase II-dependent promoters and rDNA spacer sequences
AtMsrB2 AtMsrB7 AtMsrB9 AtPMSR1 AtPMSR3 OsMsrA2.1 PtMsrA4.1 PtMsrA4.2 PtMsrA5 PtMsrB3.2	1	Myb domain protein 46
AtMsrA5 AtMsrB2 AtMsrB5 AtMsrB7 AtMsrB9 AtPMSR1 AtPMSR4 OsMsrA5 PtMsrA4.2 PtMsrB5	1	SQUA promoter binding proteins
AtMsrB1 AtMsrB2 AtMsrB4 AtMsrB5 AtPMSR1 AtPMSR4 OsMsrB1 OsMsrB3 PtMsrA5 PtMsrB3.2	1	Common plant regulatory factor (CPRF) from parsley
AtMsrB3 AtMsrB4 AtMsrB6 AtMsrB8 AtMsrB9 AtPMSR1 AtPMSR4 PtMsrA4.1 PtMsrB1 PtMsrB3.2	1	SEPALLATA3, transcription factor AGL9
AtMsrA5 AtMsrB1 AtMsrB9 AtPMSR1 OsMsrB1 PtMsrA4.1 PtMsrA4.2 PtMsrA5 PtMsrB3.1 PtMsrB3.2	1	Target of early activation tagged (EAT) 2 (secondary DNA binding preference)
AtMsrA5 AtMsrB2 AtMsrB3 AtMsrB4 AtMsrB5 AtPMSR1 OsMsrA4 OsMsrA5 PtMsrA4.2 PtMsrA5	1	AGL2, Arabidopsis MADS-domain protein AGAMOUS-like 2
AtMsrA5 AtMsrB6 AtPMSR1 OsMsrA2.1 OsMsrA4 OsMsrB3 PtMsrA2.1 PtMsrA2.2 PtMsrB3.2 PtMsrB5	1	Dof zinc finger protein DOF1.6 (AT1G47655)
AtMsrB9 AtPMSR1 OsMsrA2.1 OsMsrA2.2 OsMsrA4 OsMsrA5 OsMsrB1 PtMsrA5 PtMsrB1 PtMsrB3.1	1	Calmodulin-binding transcription activator 1 (AtSR2)
AtMsrA5 AtMsrB5 AtMsrB6 AtMsrB9 AtPMSR2 AtPMSR3 OsMsrA2.1 OsMsrA5 OsMsrB1 PtMsrA4.2	1	GATA transcription factor 19 (HANABA TARANU LIKE 2, HANL2)
AtMsrB4 AtMsrB8 AtPMSR2 AtPMSR3 OsMsrA4 OsMsrA5 OsMsrB5 PtMsrA2.2 PtMsrB3.2 PtMsrB5	1	MYB protein from wheat
AtMsrB3 AtMsrB6 AtMsrB7 AtMsrB8 AtPMSR2 AtPMSR4 OsMsrA2.1 PtMsrA2.1 PtMsrA2.2 PtMsrA4.2	1	Myc recognition sequences
AtMsrB5 AtPMSR2 AtPMSR4 OsMsrA2.1 OsMsrA2.2 OsMsrA5 OsMsrB3 OsMsrB5 PtMsrA4.1 PtMsrA4.2	1	Arabidopsis leucine zipper protein TGA1
AtMsrA5 AtMsrB1 AtMsrB2 AtMsrB3 AtMsrB5 AtPMSR2 OsMsrA2.2 OsMsrA5 OsMsrB1 OsMsrB3	1	Dehydration-responsive element-binding protein A-4
AtMsrB3 AtMsrB4 AtMsrB5 AtMsrB6 AtMsrB9 AtPMSR2 OsMsrA5 OsMsrB5 PtMsrA5 PtMsrB3.2	1	Sunflower homeodomain leucine-zipper protein Hahb-4
AtMsrA5 AtMsrB3 AtMsrB4 AtMsrB6 AtPMSR3 AtPMSR4 OsMsrA4 OsMsrA5 OsMsrB3 PtMsrB3.2	1	Squamosa promoter-binding-like protein 5

AtMsrA5 AtMsrB5 AtMsrB7 AtMsrB8 AtPMSR3 AtPMSR4 OsMsrB3 OsMsrB5 PtMsrA5 PtMsrB3.1	1	E2F class I sites
AtMsrA5 AtMsrB2 AtMsrB4 AtMsrB5 AtPMSR3 OsMsrA2.2 OsMsrA5 OsMsrB3 PtMsrA4.1 PtMsrB3.1	1	Drosophila motif ten element
AtMsrA5 AtMsrB3 AtMsrB5 AtPMSR3 OsMsrA2.2 OsMsrA5 OsMsrB1 OsMsrB3 OsMsrB5 PtMsrB3.1	1	Trihelix-domain transcription factor VFP5 (AT5G05550)
AtMsrB1 AtMsrB3 AtMsrB5 AtPMSR3 OsMsrA2.1 OsMsrB1 OsMsrB5 PtMsrA4.2 PtMsrB3.1 PtMsrB3.2	1	Agamous, required for normal flower development, similarity to SRF (human) and MCM (yeast) proteins
AtMsrB1 AtMsrB4 AtMsrB5 AtMsrB6 AtPMSR3 OsMsrA2.1 OsMsrB1 OsMsrB5 PtMsrA2.2 PtMsrB1	1	Trihelix transcription factor GTL2
AtMsrA5 AtMsrB5 AtPMSR4 OsMsrA2.1 OsMsrA5 OsMsrB1 OsMsrB5 PtMsrA4.2 PtMsrA5 PtMsrB3.2	1	MybSt1 (Myb Solanum tuberosum 1) with a single myb repeat
AtMsrB2 AtMsrB4 AtMsrB5 AtMsrB6 AtMsrB7 AtMsrB9 OsMsrA2.2 PtMsrA5 PtMsrB1 PtMsrB5	1	Phytochrome interacting factor 4
AtMsrB1 AtMsrB4 AtMsrB5 AtPMSR1 AtPMSR4 OsMsrA5 OsMsrB1 OsMsrB3 PtMsrA5	1	BBES1/BZR1-like protein 3 (AT4G18890)
AtMsrB2 AtMsrB4 AtMsrB6 AtMsrB9 AtPMSR1 AtPMSR4 OsMsrA5 PtMsrB1 PtMsrB5	1	Mycorrhiza transcription factor
AtMsrB4 AtMsrB7 AtPMSR1 AtPMSR4 OsMsrA2.2 OsMsrA4 OsMsrB1 OsMsrB5 PtMsrB5	1	Squamosa promoter-binding-like protein 1
AtMsrB2 AtMsrB6 AtMsrB7 AtMsrB8 AtPMSR1 OsMsrA2.1 OsMsrA4 OsMsrB3 PtMsrA2.1	1	WRKY DNA-binding protein 45
AtMsrB5 AtMsrB8 AtMsrB9 AtPMSR1 OsMsrA4 OsMsrA5 PtMsrA2.1 PtMsrA4.2 PtMsrB5	1	Homeobox protein 33
AtMsrB5 AtMsrB9 AtPMSR1 OsMsrA2.1 OsMsrA4 OsMsrB1 OsMsrB3 PtMsrB1 PtMsrB3.1	1	Anther-specific myb gene from tobacco
AtMsrB5 AtPMSR2 AtPMSR3 AtPMSR4 OsMsrA2.1 OsMsrB3 OsMsrB5 PtMsrA4.1 PtMsrA4.2	1	Basic region / leucine zipper motif 60
AtMsrA5 AtMsrB2 AtMsrB3 AtMsrB4 AtPMSR2 AtPMSR3 OsMsrA4 OsMsrA5 OsMsrB3	1	Heat stress transcription factor A-6b
AtMsrB2 AtMsrB5 AtMsrB6 AtMsrB7 AtPMSR2 AtPMSR3 OsMsrA4 PtMsrA2.1 PtMsrB1	1	KANADI 4 (Aberrant Testa Shape) (secondary DNA binding preference)
AtMsrB1 AtMsrB2 AtPMSR2 AtPMSR4 OsMsrA2.2 OsMsrA5 OsMsrB1 OsMsrB3 PtMsrB3.1	1	Callose Defective Microspore 1 (zinc finger CCCH domain-containing protein 15)
AtMsrB3 AtMsrB5 AtMsrB7 AtPMSR2 AtPMSR4 PtMsrA2.2 PtMsrA4.2 PtMsrB3.2 PtMsrB5	1	Myb family transcription factor REVEILLE 1
AtMsrB6 AtMsrB7 AtMsrB8 AtPMSR2 AtPMSR4 OsMsrA2.1 OsMsrA5 OsMsrB3 PtMsrB1	1	Oryza sativa bZIP protein 8

AtMsrB1 AtMsrB2 AtMsrB3 AtMsrB4 AtPMSR2 OsMsrA2.1 OsMsrA5 PtMsrA2.2 PtMsrB5	1	Basic leucine-zipper 52
AtMsrB1 AtMsrB4 AtMsrB5 AtPMSR2 OsMsrA5 OsMsrB1 PtMsrA2.2 PtMsrA5 PtMsrB3.2	1	Maize activator P of flavonoid biosynthetic genes
AtMsrA5 AtMsrB5 AtMsrB6 AtMsrB9 AtPMSR3 PtMsrA4.1 PtMsrA5 PtMsrB3.1 PtMsrB5	1	Late Meristem Identity 1 (AtHB51, LMI1)
AtMsrB1 AtMsrB6 AtMsrB9 AtPMSR3 OsMsrA5 PtMsrA4.2 PtMsrA5 PtMsrB3.1 PtMsrB5	1	Reproductive Meristem 1
AtMsrB1 AtPMSR3 OsMsrA4 PtMsrA2.1 PtMsrA2.2 PtMsrA4.1 PtMsrA5 PtMsrB3.1 PtMsrB3.2	1	High mobility group I/Y-like protein isolated from pea
AtMsrB4 AtMsrB5 AtMsrB9 AtPMSR3 OsMsrA2.2 OsMsrA4 OsMsrA5 OsMsrB3 PtMsrB5	1	ABORTED MICROSPORES
AtMsrB5 AtMsrB9 AtPMSR3 OsMsrA5 OsMsrB5 PtMsrA4.1 PtMsrA4.2 PtMsrA5 PtMsrB3.1	1	Myb domain protein 121
AtMsrA5 AtMsrB1 AtMsrB5 AtPMSR4 OsMsrA2.1 OsMsrA2.2 OsMsrB1 PtMsrA2.1 PtMsrA4.2	1	Ethylene-responsive transcription factor RAP2-6 (secondary DNA binding preference)
AtMsrB1 AtMsrB2 AtMsrB9 AtPMSR4 OsMsrA2.2 OsMsrA4 OsMsrA5 OsMsrB3 PtMsrB1	1	Coupling element 3 (CE3), non-ACGT ABRE
AtMsrA5 AtMsrB1 AtMsrB2 OsMsrA4 OsMsrA5 OsMsrB1 OsMsrB3 OsMsrB5 PtMsrB5	1	M-phase-specific activators (NtmybA1, NtmybA2, NtmybB)
AtMsrA5 AtMsrB8 AtMsrB9 OsMsrA4 OsMsrB3 PtMsrA2.1 PtMsrA4.1 PtMsrB1 PtMsrB3.1	1	Myb family transcription factor AT1G74840
AtMsrA5 OsMsrA2.1 OsMsrA2.2 OsMsrA5 PtMsrA2.2 PtMsrA4.1 PtMsrA4.2 PtMsrB3.1 PtMsrB5	1	Sugar and ABA responsive element conserved in several rbcS promoters
AtMsrB1 AtMsrB2 AtMsrB5 OsMsrB1 PtMsrA2.2 PtMsrA4.1 PtMsrA4.2 PtMsrA5 PtMsrB5	1	HDZip class I protein ATHB5
AtMsrB4 OsMsrA2.1 OsMsrA2.2 OsMsrA4 OsMsrA5 PtMsrA2.2 PtMsrA4.1 PtMsrA4.2 PtMsrB3.2	1	Member of the EPF family of zinc finger transcription factors
AtMsrB6 OsMsrA2.1 OsMsrA2.2 OsMsrA5 OsMsrB1 PtMsrA2.2 PtMsrA4.2 PtMsrB1 PtMsrB5	1	ERSE I (ER stress-response element I)-like motif
AtMsrB9 AtPMSR1 AtPMSR2 AtPMSR4 OsMsrA5 OsMsrB5 PtMsrA4.1 PtMsrB5	1	SHI related sequence 1 (STYLISH 1) (secondary DNA binding preference)
AtMsrB7 AtPMSR1 AtPMSR2 OsMsrA2.2 OsMsrA4 OsMsrA5 OsMsrB5 PtMsrA2.2	1	FAR-RED ELONGATED HYPOCOTYLS 3 and FAR-RED IMPAIRED RESPONSE 1
AtMsrB3 AtPMSR1 AtPMSR3 OsMsrA2.2 OsMsrB5 PtMsrA4.2 PtMsrB3.2 PtMsrB5	1	Myb domain protein 67 (ATY53)
AtMsrA5 AtMsrB5 AtMsrB9 AtPMSR1 AtPMSR4 OsMsrB1 PtMsrA4.1 PtMsrB1	1	Myb domain protein R1 (ATMYB44)
AtMsrB5 AtPMSR1 OsMsrA2.1 OsMsrA2.2 OsMsrA5 OsMsrB5 PtMsrA2.1 PtMsrA2.2	1	C-repeat/dehydration response element

AtMsrA5 AtMsrB4 AtMsrB6 AtPMSR2 AtPMSR3 OsMsrB1 OsMsrB5 PtMsrA2.1	1	Two-component response regulator ARR14
AtMsrA5 AtMsrB5 AtPMSR2 AtPMSR3 OsMsrA4 OsMsrA5 PtMsrA5 PtMsrB3.1	1	Asymmetric leaves2-like 18 (LOB domain-containing protein 16)
AtMsrB1 AtMsrB5 AtPMSR2 AtPMSR3 OsMsrA5 OsMsrB3 PtMsrA2.1 PtMsrB1	1	Plant specific floral meristem identity gene LEAFY (LFY)
AtMsrB6 AtMsrB9 AtPMSR2 AtPMSR3 OsMsrA2.1 PtMsrA2.1 PtMsrA5 PtMsrB5	1	Putative cis-acting element in various PAL and 4CL gene promoters
AtMsrB3 AtPMSR2 AtPMSR4 OsMsrB5 PtMsrA2.1 PtMsrA5 PtMsrB1 PtMsrB5	1	Hordeum vulgare Myb-related CAB-promoter-binding protein 1
AtMsrA5 AtMsrB3 AtPMSR2 OsMsrA5 PtMsrA2.1 PtMsrA5 PtMsrB3.1 PtMsrB3.2	1	HD-ZIP class III protein ATHB9
AtMsrB1 AtMsrB4 AtMsrB8 AtPMSR2 OsMsrA4 PtMsrA5 PtMsrB3.2 PtMsrB5	1	Myb-domain transcription factor werewolf
AtMsrB4 AtMsrB8 AtPMSR2 OsMsrA4 OsMsrA5 PtMsrA2.1 PtMsrA2.2 PtMsrB1	1	Myb domain protein 31 (AtMYB31)
AtMsrA5 AtMsrB2 AtMsrB4 AtPMSR3 AtPMSR4 PtMsrA4.1 PtMsrB3.2 PtMsrB5	1	AGL1, Arabidopsis MADS-domain protein AGAMOUS-like 1
AtMsrA5 AtMsrB9 AtPMSR3 AtPMSR4 OsMsrA2.2 OsMsrA4 PtMsrA4.2 PtMsrB1	1	VirE2-interacting protein 1
AtMsrA5 AtMsrB1 AtMsrB3 AtMsrB5 AtPMSR3 OsMsrA2.1 PtMsrA2.2 PtMsrA4.1	1	NAC domain containing protein 38
AtMsrA5 AtMsrB3 AtMsrB5 AtPMSR3 OsMsrA2.1 PtMsrA4.1 PtMsrA5 PtMsrB3.1	1	Homeobox-leucine zipper protein ATHB-53
AtMsrA5 AtMsrB3 AtPMSR3 OsMsrA4 OsMsrA5 PtMsrB1 PtMsrB3.2 PtMsrB5	1	Heat shock transcription factor C1 (secondary DNA binding preference)
AtMsrB7 AtPMSR4 OsMsrA2.1 OsMsrA2.2 OsMsrA5 OsMsrB3 PtMsrA5 PtMsrB5	1	bZIP transcription factor from Antirrhinum majus
AtMsrA5 AtMsrB1 AtMsrB2 AtMsrB4 AtMsrB8 OsMsrA2.1 OsMsrA4 OsMsrB1	1	WRKY DNA-binding protein 20
AtMsrA5 AtMsrB2 AtMsrB5 AtMsrB6 PtMsrA2.1 PtMsrA4.2 PtMsrB1 PtMsrB3.1	1	Ribosomal protein box, appears unique to plant RP genes and genes associated with gene expression
AtMsrA5 AtMsrB4 AtMsrB6 AtMsrB7 OsMsrA4 OsMsrA5 PtMsrB1 PtMsrB3.1	1	Myb domain protein 33
AtMsrB1 AtMsrB3 AtMsrB6 AtMsrB7 OsMsrB3 PtMsrA4.1 PtMsrA4.2 PtMsrA5	1	Transcription factor NAC2
AtMsrB1 AtMsrB4 AtMsrB6 OsMsrA2.1 OsMsrA2.2 OsMsrB1 OsMsrB3 PtMsrA5	1	Brassinosteroid insensitive 1-EMS suppressor 1
AtMsrB1 AtMsrB4 AtMsrB9 OsMsrA4 OsMsrA5 PtMsrB1 PtMsrB3.1 PtMsrB3.2	1	Rice MYB proteins with single DNA binding domains, binding to the amylase element (TATCCA)
AtMsrB2 AtMsrB3 AtMsrB9 OsMsrA2.1 OsMsrA4 PtMsrA2.1 PtMsrA2.2 PtMsrA4.2	1	Myb domain protein 59
AtMsrB2 OsMsrA2.1 OsMsrA2.2 OsMsrA4 OsMsrA5 OsMsrB1 OsMsrB5 PtMsrB1	1	ABA (abscisic acid) inducible transcriptional activator
AtMsrB3 AtMsrB4 AtMsrB5 AtMsrB7 OsMsrA2.1 OsMsrB1 OsMsrB3 PtMsrA4.1	1	Two-component response regulator ARR14 (secondary DNA binding preference)
AtMsrB3 AtMsrB6 AtMsrB8 PtMsrA2.1 PtMsrB1 PtMsrB3.1 PtMsrB3.2 PtMsrB5	1	Myb domain protein 93

AtMsrB3 AtMsrB7 AtPMSR1 AtPMSR3 AtPMSR4 PtMsrA2.1 PtMsrA4.1	1	Myb family transcription factor At5g56840
AtMsrB2 AtMsrB7 AtPMSR1 AtPMSR3 PtMsrA2.1 PtMsrA4.1 PtMsrB3.1	1	High Cambial Activity 2 (AT5G62940, DOF5.6)
AtMsrB4 AtMsrB7 AtMsrB9 AtPMSR1 AtPMSR3 OsMsrA4 OsMsrB3	1	NAC domain containing protein 58
AtMsrB4 AtPMSR1 AtPMSR3 OsMsrA4 OsMsrA5 PtMsrB3.1 PtMsrB5	1	Arabidopsis Telo-box interacting protein related to the conserved animal protein Pur-alpha
AtMsrB9 AtPMSR1 OsMsrA2.2 OsMsrA4 OsMsrA5 OsMsrB3 PtMsrB1	1	Oriza sativa leaf and tiller angle increased controller
AtMsrB1 AtMsrB9 AtPMSR2 AtPMSR4 OsMsrA2.1 OsMsrA5 OsMsrB3	1	bZIP factors DPBF-1 and 2 (Dc3 promoter binding factor-1 and 2)
AtMsrB1 AtMsrB2 AtMsrB5 AtMsrB9 AtPMSR2 OsMsrB5 PtMsrA2.1	1	MADS-box protein GORDITA (AGL63)
AtMsrB1 AtMsrB2 AtPMSR3 AtPMSR4 PtMsrA5 PtMsrB3.1 PtMsrB3.2	1	TCP class I transcription factor (Arabidopsis)
AtMsrA5 AtPMSR3 OsMsrA2.2 OsMsrA5 OsMsrB1 OsMsrB3 PtMsrB1	1	Myb domain protein 88
AtMsrB1 AtPMSR3 OsMsrA2.2 OsMsrB1 OsMsrB5 PtMsrA4.2 PtMsrB1	1	Target of early activation tagged 1 (RAP2.7)
AtMsrB7 AtMsrB8 AtMsrB9 AtPMSR3 OsMsrA2.1 PtMsrA2.1 PtMsrA5	1	Early-phytochrome-responsive 1 (REVEILLE 7)
AtMsrA5 AtMsrB4 AtMsrB7 AtPMSR4 OsMsrA2.1 OsMsrA5 OsMsrB3	1	GATA transcription factor 4
AtMsrA5 AtMsrB5 OsMsrA2.1 OsMsrA2.2 OsMsrA4 OsMsrA5 OsMsrB1	1	Promoter elements involved in MgProto (Mg-protoporphyrin IX) and light-mediated induction
AtMsrA5 AtMsrB9 OsMsrA2.1 PtMsrA2.1 PtMsrA4.2 PtMsrB1 PtMsrB5	1	Arabidopsis thaliana class A heat shock factor 1a
AtMsrB3 AtMsrB4 AtMsrB6 AtMsrB9 PtMsrA2.2 PtMsrB1 PtMsrB5	1	ULT1 interacting factor 1 (AT4G37180)
AtMsrB5 OsMsrA2.1 OsMsrA2.2 OsMsrA4 OsMsrA5 OsMsrB1 OsMsrB3	1	Mitochondrial transcription termination factor family protein (mTERF)
OsMsrA2.1 OsMsrA2.2 OsMsrA4 PtMsrA4.2 PtMsrA5 PtMsrB3.2 PtMsrB5	1	Anaerobic basic leucine zipper
OsMsrA2.1 OsMsrA2.2 OsMsrA4 OsMsrA5 OsMsrB1 OsMsrB3 PtMsrB1	1	H. vulgare dehydration-response factor 1
OsMsrA2.1 OsMsrA2.2 OsMsrA4 OsMsrA5 OsMsrB3 OsMsrB5 PtMsrB1	1	ABA insensitive protein 4 (ABI4)
AtPMSR1 AtPMSR2 OsMsrA5 OsMsrB1 PtMsrA2.1 PtMsrA4.2	1	AC-type motifs, MYB46/MYB83-responsive elements
AtMsrA5 AtMsrB6 AtPMSR1 OsMsrB5 PtMsrB1 PtMsrB5	1	Iron-deficiency-responsive element 1 (IDEF1)
AtMsrB2 AtPMSR2 AtPMSR3 OsMsrB1 PtMsrA5 PtMsrB3.1	1	DREB and EAR motif protein 3
AtMsrB7 AtMsrB8 AtPMSR2 AtPMSR3 OsMsrA2.1 OsMsrB1	1	Flowering bHLH 3
AtMsrA5 AtMsrB3 AtMsrB7 AtPMSR2 OsMsrB5 PtMsrA4.1	1	Tomato MADS-box transcription factor MADS-RIN
AtMsrA5 AtMsrB4 AtMsrB6 AtPMSR2 OsMsrB1 PtMsrA2.1	1	Tobacco bHLH transcription factor MYC2
AtMsrA5 AtMsrB5 AtMsrB9 AtPMSR2 PtMsrB3.2 PtMsrB5	1	Upstream sequence elements in the promoters of U-snRNA genes of higher plants
AtMsrB1 AtMsrB2 AtPMSR2 OsMsrA4 PtMsrA5 PtMsrB3.1	1	Cis-acting element conserved in various PAL and 4CL promoters
AtMsrB1 AtMsrB5 AtPMSR2 OsMsrA5 OsMsrB3 PtMsrB5	1	GATA transcription factor 11
AtMsrB4 AtMsrB7 AtPMSR2 OsMsrA2.1 PtMsrA2.1 PtMsrA5	1	Elicitor response element
AtPMSR2 OsMsrA2.1 OsMsrA2.2 OsMsrA4 PtMsrA4.1 PtMsrB1	1	Far-red elongated hypocotyls 3 protein
AtMsrB5 AtPMSR3 AtPMSR4 OsMsrA5 PtMsrA4.1 PtMsrB3.1	1	Myb domain protein 101 (ATM1)
AtMsrA5 AtMsrB1 AtMsrB9 AtPMSR3 OsMsrA5 OsMsrB3	1	ETTIN (secondary DNA binding preference)
AtMsrA5 AtPMSR3 OsMsrA2.2 OsMsrA4 OsMsrB1 OsMsrB3	1	GCC-box, ethylene-responsive element (ERE)
AtPMSR3 OsMsrA2.2 OsMsrA5 OsMsrB3 OsMsrB5 PtMsrA2.1	1	Dwarf and Delayed Flowering 1

AtMsrB1 AtMsrB2 AtMsrB9 AtPMSR4 PtMsrA5 PtMsrB1	1	Phytochrome B-regulated transcription factor PIF3 (nuclear-localized bHLH-protein)
AtMsrB1 AtMsrB2 AtPMSR4 PtMsrA5 PtMsrB3.1 PtMsrB3.2	1	Promoter motif enriched in a cluster of Arabidopsis genes co-expressed during immune response
AtMsrB3 AtMsrB9 AtPMSR4 OsMsrA4 OsMsrB3 PtMsrB3.1	1	DIVARICATA 6 (myb/SANT-like DNA-binding domain-containing protein AT5G05790)
AtMsrA5 AtMsrB2 AtMsrB3 AtMsrB7 AtMsrB8 PtMsrB3.1	1	Myb domain protein 3R-5
AtMsrA5 AtMsrB2 AtMsrB3 OsMsrA4 OsMsrB1 PtMsrA2.1	1	SHI related sequence 1 (STYLISH 1)
AtMsrB2 AtMsrB5 AtMsrB7 OsMsrA2.2 OsMsrA4 PtMsrA2.1	1	FAR1-related sequence 9
OsMsrA2.1 OsMsrA2.2 OsMsrA4 OsMsrA5 OsMsrB3 OsMsrB5	1	LOB domain-containing protein 13
AtMsrB7 AtPMSR1 AtPMSR2 AtPMSR3 PtMsrA4.2	1	NAC domain containing protein 92 (NAC6)
AtMsrB6 AtMsrB7 AtPMSR1 AtPMSR2 PtMsrB3.1	1	Myb-like HTH transcriptional regulator-like protein (G2-like family protein)
AtPMSR1 AtPMSR2 OsMsrA4 OsMsrA5 PtMsrA4.1	1	Putative cis-acting element on various PAL and 4CL gene promoters
AtPMSR1 AtPMSR3 AtPMSR4 OsMsrB1 PtMsrB3.2	1	Myb/SANT-like DNA-binding domain-containing protein MYBS1
AtMsrB4 AtMsrB5 AtMsrB7 AtPMSR1 AtPMSR4	1	Dwarf and Delayed Flowering 2
AtMsrB1 AtMsrB3 AtMsrB8 AtPMSR1 PtMsrA4.1	1	Short Vegetative Phase
AtMsrB4 AtPMSR1 OsMsrA2.2 OsMsrA4 OsMsrA5	1	Squamosa promoter-binding-like protein 1 (secondary DNA binding preference)
AtMsrB5 AtMsrB7 AtPMSR1 OsMsrA2.2 PtMsrA2.1	1	Basic Pentacysteine 1
AtMsrB2 AtMsrB6 AtPMSR2 PtMsrA4.2 PtMsrB3.2	1	Myb family transcription factor AT1G19000
AtMsrB4 AtMsrB8 AtPMSR2 OsMsrA2.1 OsMsrB5	1	WRKY transcription factor 7
AtMsrB5 AtMsrB7 AtMsrB8 AtPMSR2 OsMsrA2.2	1	bZIP transcription factor Elongated Hypocotyl 5
AtMsrB6 AtMsrB8 AtPMSR2 OsMsrA4 OsMsrB5	1	Floral homeotic protein APETALA1
AtPMSR2 OsMsrA4 OsMsrB1 PtMsrA2.2 PtMsrB3.1	1	DOF Affecting Germination 2
AtMsrB5 AtMsrB9 AtPMSR3 AtPMSR4 PtMsrA4.2	1	Auxin Response Element
AtPMSR3 OsMsrA2.1 OsMsrA2.2 OsMsrA5 OsMsrB5	1	Myb related transcriptional activator, direct ortholog of S.pombe CDC5 protein
AtMsrA5 AtMsrB4 AtMsrB6 PtMsrA5 PtMsrB3.1	1	Zinc finger CCCH domain-containing protein 67 (AT5G63260)
AtMsrA5 AtMsrB8 OsMsrA2.1 OsMsrB1 PtMsrB1	1	TCP domain protein 16
AtMsrA5 OsMsrA2.2 OsMsrA4 PtMsrA4.1 PtMsrB1	1	Myb domain protein 61
AtMsrB1 AtMsrB2 AtMsrB6 AtMsrB7 PtMsrA5	1	Trihelix transcription factor GT-2
AtMsrB1 AtMsrB3 AtMsrB6 PtMsrB1 PtMsrB5	1	Homeobox-leucine zipper protein HAT2
AtMsrB1 AtMsrB3 OsMsrA2.2 OsMsrA5 PtMsrA2.2	1	myb domain protein 58
AtMsrB1 AtMsrB7 AtMsrB8 AtMsrB9 OsMsrB5	1	OCS-like elements
AtMsrB2 AtMsrB6 OsMsrA2.2 OsMsrA5 OsMsrB1	1	Rice iron-related transcription factor 2
AtMsrB3 AtMsrB4 AtMsrB7 OsMsrA2.2 PtMsrB1	1	Transcription factor DYSFUNCTIONAL TAPETUM 1
AtMsrB3 AtMsrB5 PtMsrA4.1 PtMsrA5 PtMsrB1	1	Dof2 - single zinc finger transcription factor
AtMsrB3 AtMsrB7 OsMsrA4 PtMsrA2.1 PtMsrA4.2	1	Myb domain protein 27

AtMsrB3 OsMsrA5 PtMsrA4.1 PtMsrA4.2 PtMsrB5	1	Heat stress transcription factor B-2a (HSF6)
AtMsrB4 AtMsrB6 OsMsrA4 PtMsrB3.1 PtMsrB3.2	1	Myb family transcription factor At5g61620
AtMsrB5 AtMsrB6 AtMsrB9 OsMsrA4 PtMsrB1	1	Myb hypocotyl elongation-related (AT5G47390, KUODA1)
AtMsrB6 AtMsrB7 PtMsrA4.1 PtMsrA4.2 PtMsrB3.2	1	Suppressor of overexpression of CO 1 (AGL20)
AtMsrB6 AtMsrB9 OsMsrA2.1 OsMsrA5 OsMsrB1	1	Zinc finger and SCAN domain containing 4
AtMsrB6 OsMsrB3 PtMsrA4.2 PtMsrB3.1 PtMsrB3.2	1	B-box domain protein 31
AtMsrB7 AtMsrB8 PtMsrA2.1 PtMsrA4.2 PtMsrB1	1	Squamosa promoter-binding-like protein 13
OsMsrA2.1 OsMsrA2.2 OsMsrA4 OsMsrA5 OsMsrB3	2	Ethylene-responsive transcription factor ERF057 (AT5G65130) Ethylene and salt inducible 3
OsMsrA2.2 OsMsrA4 OsMsrA5 OsMsrB1 OsMsrB5	1	Dehydration-responsive element-binding protein 2C
AtMsrB3 AtPMSR1 AtPMSR4 PtMsrB3.2	1	Telomere repeat-binding protein 1
AtMsrB3 AtPMSR1 OsMsrA5 OsMsrB1	1	Ethylene-responsive elements (ERE) and jasmonate- and elicitor-responsive elements (JERE)
AtMsrB4 AtMsrB5 AtPMSR1 PtMsrA5	1	Transcription factor Big Petal (AT1G59640)
AtMsrB9 AtPMSR2 AtPMSR3 PtMsrA5	1	Myb domain protein 96
AtMsrB1 AtPMSR2 OsMsrB3 OsMsrB5	1	Arabidopsis NAC domain containing protein 92 (ATNAC2/ATNAC6)
AtMsrB3 AtMsrB9 AtPMSR2 PtMsrA2.2	1	WRKY transcription factor 70
AtMsrB4 AtMsrB9 AtPMSR2 PtMsrB3.1	1	R2R3-MYB transcription factor AtMYB77
AtMsrB5 AtPMSR2 OsMsrB5 PtMsrB1	1	Transcription factor TGA4 (OBF4)
AtMsrB6 AtMsrB9 AtPMSR2 OsMsrA4	1	Brassinazole-resistant 1 protein
AtMsrB2 AtPMSR3 OsMsrA2.1 PtMsrA4.1	1	Maize C1 myb-domain protein
AtMsrB9 AtPMSR3 PtMsrA2.1 PtMsrB3.2	1	WRKY transcription factor 22
AtMsrB2 AtPMSR4 OsMsrA2.1 PtMsrB5	1	DREB and EAR motif protein 4 (RAP2.10)
AtPMSR4 OsMsrA2.2 OsMsrA5 OsMsrB1	1	Tobacco bZip transcription activator (TAF-1)
AtMsrA5 OsMsrA5 OsMsrB1 PtMsrA2.1	1	Ethylene-responsive transcription factor ERF043 (AT4G32800)
AtMsrB2 AtMsrB7 PtMsrA5 PtMsrB1	1	Dof zinc finger protein DOF4.2
AtMsrB2 PtMsrB3.1 PtMsrB3.2 PtMsrB5	1	Homeobox-leucine zipper protein ATHB-20
OsMsrA4 OsMsrB1 OsMsrB3 PtMsrA2.2	1	Transcription factor TGA2 (secondary DNA binding preference)
OsMsrA2.1 OsMsrA2.2 OsMsrA4 OsMsrA5	1	Ethylene-responsive transcription factor ERF017 (AT1G19210)
OsMsrA2.2 OsMsrA5 OsMsrB1 OsMsrB3	3	Ethylene-responsive transcription factor 1A (AT4G17500) TCP class I transcription factor Human motif ten element
OsMsrA2.2 OsMsrA4 OsMsrA5 OsMsrB3	2	Dehydration-responsive element-binding protein 2G (AT5G18450) FAR-RED Impaired Response 1
OsMsrA2.2 OsMsrA4 OsMsrB1 OsMsrB3	1	Ethylene-responsive transcription factor 1B (AT3G23240)
OsMsrA2.2 OsMsrA4 OsMsrB3 OsMsrB5	1	Ethylene-responsive transcription factor RAP2-6
AtMsrB3 AtPMSR1 OsMsrB5	1	Ethylene-responsive transcription factor ERF055 (AT1G36060)
AtPMSR1 OsMsrB5 PtMsrA5	1	Dehydration response element-binding protein 19

AtPMSR1 OsMsrB3 PtMsrB1	1	DREB and EAR motif protein 4 (RAP2.10) (secondary DNA binding preference)
AtMsrA5 AtPMSR2 AtPMSR3	1	NAC (No Apical Meristem) domain transcriptional regulator superfamily protein
AtPMSR2 AtPMSR4 PtMsrA2.1	1	Myb domain protein 74
AtMsrB7 AtPMSR2 OsMsrA2.2	1	Wheat NAC-domain DNA binding factor
AtMsrB6 AtPMSR3 OsMsrA4	1	LHY/CCA1-like 1 (REVEILLE 4)
AtPMSR3 PtMsrA2.1 PtMsrA5	1	Sugar responsive elements
AtPMSR3 OsMsrA4 PtMsrB3.1	1	Dof zinc finger protein DOF5.4 (OBF binding protein 4)
AtMsrB1 AtPMSR4 PtMsrA4.2	1	NAC domain containing protein 17
AtMsrB6 AtPMSR4 PtMsrA4.1	1	Transcription factor TCP22 (AT1G72010)
AtMsrA5 AtMsrB8 PtMsrA5	1	Homeobox-leucine zipper protein ATHB-13
AtMsrA5 OsMsrB3 PtMsrA5	1	Dof zinc finger protein DOF3.5 (AT3G52440)
AtMsrB1 AtMsrB2 PtMsrB5	1	HRS1 Homolog 3 (AT1G25550)
AtMsrB2 OsMsrA2.1 OsMsrA4	1	Abscisic acid insensitive 5 (bZIP12, EEL)
AtMsrB3 OsMsrB1 OsMsrB3	1	Myb domain protein 119
AtMsrB3 OsMsrA5 PtMsrA2.1	1	Homeobox-leucine zipper protein ATHB-21
AtMsrB3 PtMsrA2.2 PtMsrB1	1	Homeobox-leucine zipper protein ATHB-18
AtMsrB3 OsMsrB1 PtMsrB3.2	1	Silencing element binding factor - transcriptional repressor
AtMsrB4 OsMsrA2.1 OsMsrB5	1	Duplicated SANT DNA-binding domain-containing protein MYBS2
AtMsrB5 OsMsrA2.2 OsMsrA4	1	Ethylene-responsive transcription factor RAP2-3
AtMsrB6 PtMsrA4.2 PtMsrB3.2	1	Dof zinc finger protein DOF4.5
AtMsrB7 OsMsrA2.2 OsMsrA5	1	Ethylene-responsive transcription factor ERF018
AtMsrB9 OsMsrA5 PtMsrA4.1	1	Heat stress transcription factor A-4a (HSF21)
OsMsrA2.1 OsMsrA5 OsMsrB1	1	Wheat NAC-domain DNA binding factor (DNA binding site I)
OsMsrA2.1 OsMsrA5 OsMsrB3	1	FYF Up-regulating 321 Factor 1 (AT1G71450)
OsMsrA2.2 OsMsrA5 OsMsrB1	1	Ethylene-responsive transcription factor RAP2-11
OsMsrA2.2 OsMsrA5 OsMsrB3	3	Redox responsive transcription factor 1 Ethylene-responsive transcription factor 8 X gene core promoter element 1
OsMsrA2.2 OsMsrB1 OsMsrB3	2	Ethylene-responsive transcription factor RAP2-3 (secondary DNA binding preference) Target of early activation tagged (EAT) 2
OsMsrA2.2 OsMsrA4 OsMsrA5	1	Ethylene-responsive transcription factor 15 (AT2G31230)
OsMsrA2.2 OsMsrA4 OsMsrB5	1	Transcription factor TGA2
OsMsrA5 OsMsrB1	1	Phytochrome B-regulated transcription factor PIF3
AtPMSR1 AtPMSR4	2	NAM-like protein CUP-SHAPED COTYLEDON 1 BES1-interacting Myc-like protein 2

AtPMSR1 PtMsrA2.2	1	KNOTTED1 (KN1) and KNOTTED interacting protein (KIP) are TALE class homeodomain proteins. The KN1-KIP complex binds this DNA motif with high affinity.
AtMsrB8 AtPMSR2	1	NAC domain containing protein 2
AtPMSR2 PtMsrA4.1	1	BES1-interacting Myc-like protein 1
AtPMSR2 PtMsrA5	1	WRKY DNA-binding protein 40
AtPMSR3 OsMsrA5	1	Ethylene-responsive transcription factor ERF112 (AT2G33710)
AtMsrB2 AtPMSR3	1	HBP-1a, suggested to be involved in the cell cycle-dependent expression
AtMsrB3 AtPMSR3	1	CUP-SHAPED COTYLEDON 3
AtPMSR4 OsMsrA5	1	TCP domain protein 7
AtPMSR4 OsMsrB5	2	LOB domain-containing protein 18 Initiator (INR) and downstream promoter element (DPE) with strictly maintained spacing
AtMsrA5 AtPMSR4	1	RNA polymerase III transcription initiation factor complex (TFIIIC)
AtMsrB2 AtPMSR4	1	DNA-binding protein S1FA3
AtPMSR4 PtMsrB5	1	bZIP transcription factor TGA1 (bZIP47)
AtPMSR4 OsMsrA2.1	1	VIRE2-interacting protein 1
AtMsrA5 OsMsrB3	1	Ethylene-responsive transcription factor ERF039 (AT4G16750)
AtMsrA5 AtMsrB2	1	WRKY DNA-binding protein 18
AtMsrA5 AtMsrB5	1	ANT (Arabidopsis protein AINTEGUMENTA), member of the plant-specific family of AP2/EREBP-transcription factors
AtMsrA5 OsMsrA2.1	1	Mitochondrial dysfunction motif
AtMsrB1 OsMsrA2.1	1	Basic region/leucine zipper transcription factor 68
AtMsrB2 OsMsrB1	1	Vascular related NAC-domain protein 1
AtMsrB2 PtMsrA4.1	1	Basic leucine-zipper 28
AtMsrB3 OsMsrB1	1	Homeodomain protein of the Knotted class1
AtMsrB3 PtMsrB3.1	1	Myb domain protein 55
AtMsrB4 OsMsrA5	1	PHR1-like 1 (AT5G29000)
AtMsrB4 AtMsrB5	1	BES1/BZR1 homolog 4 (AT1G78700)
AtMsrB4 AtMsrB9	1	CUP-SHAPED COTYLEDON 2
AtMsrB5 PtMsrA2.1	1	DIVARICATA 5 (VHA-B1-interacting transcription factor AT3G11280)
AtMsrB5 PtMsrA5	1	UPRE (unfolded protein response element) like motif
AtMsrB6 PtMsrA4.1	1	TCP domain protein 2 (Teosinte branched 1)
AtMsrB8 OsMsrB3	1	WRKY DNA binding protein 12
AtMsrB9 PtMsrB3.2	1	WRKY DNA binding protein 38
OsMsrB1 PtMsrA2.2	1	NAC domain containing protein 13
PtMsrA4.1 PtMsrA4.2	1	Ethylene-responsive transcription factor ERF014 (AT1G44830)
OsMsrA5 PtMsrB3.1	1	Squamosa promoter-binding-like protein 14

OsMsrA2.1 PtMsrB3.2	1	CCHC-type zinc knuckle protein
OsMsrA2.1 PtMsrB5	1	TCP domain protein 24
OsMsrA2.2 PtMsrB5	1	Myb domain protein 55 (secondary DNA binding preference)
OsMsrA2.2 OsMsrA5	4	Cytokinin Response Factor 10 Transcription factor TCP23 Ethylene-responsive transcription factor ERF019 (AT1G22810) Ethylene-responsive transcription factor ERF018 (secondary DNA binding preference)
OsMsrA2.2 OsMsrB3	1	Transcription factor II B (TFIIB) recognition element
OsMsrA5	8	Ethylene-responsive transcription factor 2 Rice bHLH protein Ethylene-responsive transcription factor 10 Nodulin consensus sequence 3 Ethylene-responsive transcription factor 115 C-repeat-binding factor 2 (DREB1B) Ethylene-responsive transcription factor 5 TCP domain protein 21 (AT5G08330)
OsMsrB1	4	Dehydration-responsive element-binding protein 2C (secondary DNA binding preference) LIM domain protein binding to a PAL-box like sequence E2F transcription factor 3 BBES1/BZR1-like protein 2 (AT4G36780)
OsMsrB3	1	Transcription factor TCP15 (AT1G69690)
OsMsrB5	1	Heat stress transcription factor A-1b (HSF3)
AtPMSR1	1	NAC domain containing protein 3 (ORE1 SISTER1)
AtPMSR3	3	WRKY transcription factor 8 R2R3-type myb-like transcription factor (IIG-type binding site) Cooperatively regulated by ethylene and jasmonate 1 (DEAR1)
AtPMSR4	2	Myb domain protein 116 RY and Sph motifs conserved in seed-specific promoters
AtMsrA5	1	Heat stress transcription factor C-1
AtMsrB1	1	NAC domain containing protein 45
AtMsrB2	4	Transcription factor TGA3 Arabidopsis NAC domain containing protein 81; ATAF2 WRKY transcription factor 55 NTM1-LIKE 6 (NAC domain containing protein 62)
AtMsrB6	1	Indeterminate(ID)-domain 11 (AtIDD11)
AtMsrB7	2	REVEILLE 5 Dof zinc finger protein DOF4.7 (AT4G38000)
AtMsrB8	2	Indeterminate(ID)-domain 5 protein (RAVEN) ABA-responsive element binding protein 3
AtMsrB9	1	Phytochrome-interacting fator 5 (PIL6)
PtMsrA2.1	2	Dof zinc finger protein DOF5.1 (AT5G02460) Dof zinc finger protein DOF2.2 (AT2G28810)
PtMsrA4.1	1	C-repeat-binding factor 3 (DREB1A)
PtMsrA4.2	1	Myb domain protein 118 (Plant Growth Activator 37, PGA37)

PtMsrA5	2	B3 domain-containing transcription factor NGA4 Myb domain protein 111 (secondary DNA binding preference)
PtMsrB3.1	2	C-repeat-binding factor 2 (DREB1C) Dof zinc finger protein DOF5.8 (AT5G66940)
OsMsrA2.2	2	Iron-dependent regulatory sequence TCP class II transcription factor
OsMsrA4	3	NAC domain containing protein 96 Ethylene-responsive transcription factor 105 Redox responsive transcription factor 1 (secondary DNA binding preference)

Table S3 List of (A) TFs and (B) REs sensitive to factors other than proteins. The TFs and REs were predicted to bind to the promoter region of 30 *Msr* gene sequences originating from the *Arabidopsis thaliana*, *Populus trichocarpa*, and *Oryza sativa* genomes. The predictions were made using MatInspector software. The abbreviation and gene identification number (ID) of each gene were derived from the NCBI database (https://www.ncbi.nlm.nih.gov/).

A)

TF NAME	ABBREVIATION	ID
Protodermal factor 2	PDF2	825828
SBF-1 (Swi4 and Swi6)	SWI6, SWI4, SBF1	856847, 850879
Homeodomain protein WUSCHEL	WUSCHEL	816305
Homeodomain GLABROUS 1	HDG1	825287
TESMIN/TSO1-like CXC 2	TCX2	827132
KANADI box	KAN, KAN1	831518
G2-like family protein	GLK1, GLK2	816579
Stomatal Carpenter 1 (dof5.7) (secondary DNA binding preference)	DOF5.7	836685
DIVARICATA 1 (myb related R-R-type factor AT5G58900)	AT5G58900	836007
Trihelix transcription factor GT-1	GT-1	837905
Transcriptional repressor BELLRINGER	BLH-9	831745
Nodulin consensus sequence 1	ENODL11	816933
RAP2.2, involved in carotenoid and tocopherol biosynthesis and in the expression of photosynthesis-related genes	RAP2.2	820643

DNA-binding storekeeper protein-related transcriptional regulator (AT4G00250)	STKL2 , AT4G00250	828046
Class I GATA factors	GATA22	828721
Homeobox protein 32	HB32	838033
Myb-like protein of Petunia hybrid (odorant 1)	ODO1	
Heterodimer of NAC-domain transcription factors GmNAC30 and GmNAC81	NAC030, NAC081	843524, 830779
Myb domain protein 96 (MYBCOV1)	MYB96	836367
Late elongated hypocotyl 1	LHY	839341
Target of early activation tagged 1 (RAP2.7)	RAP2-7	817403
Yabby transcription factor CRABS CLAW	CRC	843249
AS1/AS2 repressor complex binding motif II	AS1, AS2	842873, 818340
NAC WITH TRANSMEMBRANE MOTIF 1-LIKE 6 (NTL6/NTM1)	NAC062	824115
Storekeeper (STK), plant specific DNA binding protein important for tuber-specific and sucrose-inducible gene expression	AT4G00238	825892
GAAA motif involved in pollen specific transcriptional activation	DUO1	825217
Homeobox protein 34	HB34	822527
bZIP protein G-Box binding factor 1	GBF1	829826
Circadian clock associated 1	CCA1	819296
Homeobox-leucine zipper protein REVOLUTA (REV, IFL1)	REV	836190
AT-hook motif nuclear-localized protein 20	AHL20	827093
MADS-box protein SQUAMOSA	AP1	843244
GT2-box and GT3-box motifs	GT2	844024
WRKY DNA-binding protein 70	WRKY70	824807
Homeobox-leucine zipper protein ATHB-24	HB24	816350
HSE	HSF1	827496
APETALA2	AP2	829845
LATE ELONGATED HYPOCOTYL	LHY	839341
Zinc finger of Arabidopsis thaliana 6 (Cold induced zinc finger protein 2)	ZAT6	830313
Trihelix DNA-binding factor GT-3a	GT-3A, AT5G01380	831895
WUSCHEL-related homeobox 13	WOX13	829707
S1F, site 1 binding factor of spinach rps1 promoter	AT3G53370	824505
TESMIN/TSO1-like CXC 6 (At2G20110)	AT2G20110	816530
Cis-element involved in SA (salicylic acid) induction of secretion-related genes via NPR1	NPR1	842733
Secondary wall NAC binding elements	NAC012	840171
GAZ-like 3 (AT5G22990)	AT5G22990	832363
Myb domain protein r1 (ATMYB44)	MYBR1	836865
KH and zinc finger CCCH domain-containing protein	AT5G06770	830566

Soybean embryo factor 4	AGL15	831224
Arabidopsis 6b-interacting protein 1-like 1	ASIL1	841844
Evening element	RVE8	820117
Dof3 - single zinc finger transcription factor	DOF2	821681
Zea mays MYB-related protein 1 (transfer cell specific)	MYR1	831942
Homeobox-leucine zipper protein ATHB-23	HB23	833972
Type-B response regulator (ARR10), member of the GARP-family of plant myb-related DNA binding motifs	RR10	829322
Myb-related protein 3R-1 (PC-MYB1)	PC-MYB1	829409
Heat stress transcription factor B-3	HSFB3	818767
Maize INDETERMINATE1 zinc finger protein	IDD1	836806
Calmodulin-binding NAC protein	NTL9	829710
Myb domain protein 98	MYB98	827611
NAC with transmembrane mtif 1-like 8 (NTL8/NTM1-like 8)	NTL8	817273
Squamosa promoter-binding-like protein 9	SPL9	818820
Myb domain protein 107	MYB107	821178
Telomere repeat-binding protein 5	TRFL2	837268
AGL15, Arabidopsis MADS-domain protein AGAMOUS-like 15	AGL15	831224
PBF (MPBF)	pbf1	542353
NAC domain containing protein 103	NAC103	836527
DNA-binding protein of sweet potato that binds to the SP8a (ACTGTGTA) and SP8b (TACTATT) sequences of sporamin and beta-amylase genes	WRKY26	830601
Early Flowering MYB Protein (AT2G03500)	AT2G03500	814878
ICE (inducer of CBF expression 1), AtMYC2 (rd22BP1)	MYC2	840158
Phosphate starvation response 1	PHR1	828979
Homeobox-leucine zipper protein ATHB-6	HB6	816775
NAC domain containing protein 5	NAC005	839390
Dof1 / MNB1a - single zinc finger transcription factor	OBP2	837277
Botrytis-susceptible1 (MYB108)	MYB108	819827
Myb domain protein 3R-4	MYB3R-4	831023
High mobility group I/Y-like proteins	HMG	822505
Transcription factor of rice and barley binding to the iron deficiency-responsive cis-acting element 2 (IDE2)	FRU	817362
NIN-binding nucleotide sequences	NLP7	828502
Zinc-dependent activator protein-1, WRKY 1	ZAP1	815035
WRKY DNA-binding protein 50	WRKY50	832686

NAC with transmembrane motif 1	NTM1	828149
Opaque-2 regulatory protein	BZIP10	828219
Trihelix transcription factor GTL1	GTL1	840218
Myb family transcription factor At3g10113	At3g10113	820173
Recognition site for BZIP transcription factors that belong to the group of Opaque-2 like proteins	BZO2H1	828219
Secondary wall MYB-responsive element, MYB46 and MYB83 binding sites	MYB46<br>MYB83	831127<br>819997
WRKY plant specific zinc-finger-type factor associated with pathogen defence, W box	WRKY4	837956
Myb domain protein 52	MYB52	838374
Myb domain protein 49	MYB49	835511
Myb family transcription factor (G2-like family)	GLK1<br>GLK2	816579
B3 domain-containing transcription factor FUS3	FUS3	822293
RWP-RK domain containing 2	RKD1	838461
Growth-regulating factor 9	GRF9	819156
NAC domain containing protein 3	NAC003	839476
NAC domain containing protein 4	NAC004	837630
NAC domain containing protein 71	NAC071	827523
Nodulin consensus sequence 2	NRP1	814873
GT1-Box binding factors with a trihelix DNA-binding domain	GT-3B<br>AT2G38250	818404
Brassinazole-resistant 1	BES1	838518
Arabidopsis NAC domain containing protein 19	NAC019	841722
Tracheary-element-regulating cis-element	NAC101	836359
Homeobox 51, Late Meristem Identity 1	HB51	831723
Basic pentacysteine proteins	BPC6	834259
Oryza sativa CaM-binding transcription factor	BAG6	819232
Arabidopsis 6B-interacting protein 1-like 2 (AT3G14180)	ASIL2	820635
Paired amphipathic helix domain-containing protein	LOC9319679	9319679
Protein ethylene insensitive 3	ABI4	818614
Telomere binding protein 3 (TRB2)	TRB2	836894
ETTIN (Auxin Response Factor 3)	ARF3	817014
Phytochrome interacting factor3-like 5	PIL5	816538
NACL-inducible gene 1	NIG1	834727
AP2/ERF and B3 domain-containing transcription factor RAV1	RAV1	837886
Morning element (sequence motifs enriched in morning-phased genes)	CCA1	819296

	LHY	839341
Homeobox-leucine zipper protein ATHB-15 (INCURVATA 4)	ATHB-15	841645
Shoot-apical-meristem arrest 2 (AT5G08750)	SHA1	836498
Homeobox-leucine zipper protein ATHB-5	HB5	836656
Myb domain protein 99 (ATMYBCU15)	MYB99	836353
PHR1-like 2 (G2-like family)(AT3G24120)	AT3G24120	821998
Rice transcription activator-1 (RITA), basic leucin zipper protein, highly expressed during seed development	GBF6	829611
Homeodomain glabrous 9	HDG9	831598
Myb domain protein 57	MYB57	821113
Arabidopsis thaliana meristem layer 1	ATML1	828263
KANADI 4 (Aberrant Testa Shape)	ATS	834270
PHAVOLUTA(Homeobox-leucine zipper protein ATHB-9	PHV	839928
Binding sites for AP1, AP3-PI and AG dimers	Ap1<br>NAP<br>AG	843244<br>843282<br>827631
AG-motif binding protein 1	LOC4326175	4326175
Male sterile 188 (MYB103)	MYB108	819827
AGL3, MADS Box protein	SEP4	814898
Abscisic acid responsive elements-binding factor 2 (AREB-1)	ABF2	841095
R2R3-type myb-like transcription factor (I-type binding site)	MYB0	822415
NAC domain containing protein 87	822415	831591
WRINKLED 1	WRI1	824599
Calmodulin binding WRKY transcription factor 11	WRKY11	829282
Ethylene-responsive transcription factor SCHLAFMUTZE	SMZ	824664
Homeobox protein 40	HB40	829827
Ethylene-responsive transcription factor RAP2.1	RAP2.1	841117
P1BS, PHR1 binding sequences	PHR1	828979
Two-component response regulator ARR11	ARR11	843096
CXC domain containing TSO1-like protein 1 (TCX3)	TSO1	821849
Heat shock transcription factor C1	HSFC1	822047
Heat shock transcription factor B2A	HSFB2A	836322
Arabidopsis NAC domain containing protein 92 (ATNAC6)	NAC6	833957
Homeobox-leucine zipper protein ATHB-7	HB-7	819280
Arabidopsis thaliana signal-responsive gene1, Ca2+/ calmodulin binding protein homolog to NtER1 (tobacco early ethylene-responsive gene)	ESR1	837856
NAC secondary wall thickening promoting factor 2	NAC012	840171

bZIP transcription factor binding to OCS-elements	TGA1	836646
Salt tolerance zinc finger (ZAT10)	STZ	839666
Wheat bZIP transcription factor HBP1B (histone gene binding protein 1b)	AHBP-1B	830586
Root hair-specific element with a 2-nucleotid spacer between left part (LP) and right part (RP)	RHS17	829996
Reproductive Meristem 1	FLR1	820391
(GA)n/(CT)n binding proteins (GBP, soybean; BBR, barley)	BPC1	814724
NAC domain containing protein 16	NAC016	840317
SEF3, Soybean embryo factor 3	AGL15	831224
Myb domain protein 65	MYB65	820317
ARID/BRIGHT DNA-binding domain-containing protein	AT1G76510	843984
Squamosa promoter binding protein-like 14	SPL14	838692
LOB domain-containing protein 19	LBD19	819149
Homeobox protein 25	HB25	836666
Dof zinc finger protein DOF3.4 (OBF binding protein 1	LOC109223803	109223803
Myb domain protein 62 (BW62B)	MYB62	843161
Lateral organ boundaries	LOB	836429
Floral homeotic protein AGL15	AGL15	831224
Arabidopsis thaliana homeo box protein 1	HB-1	821138
Root hair-specific element with a 3-nucleotid spacer between left part (LP) and right part (RP)	RSH2	837853
Flowering locus C	LOC9309509	9309509
GCN4, conserved in cereal seed storage protein gene promoters	ABCF4	824619
NAC domain containing protein 79 (AT5G07680)	NAC080	830661
Myb domain protein 56	MYB56	831648
Myb domain protein 46	MYB46	831127
Common plant regulatory factor (CPRF) from parsley	APG3	825466
SEPALLATA3, transcription factor AGL9	AGL9	18438039
Target of early activation tagged (EAT) 2	TOE2	836134
AGL2, Arabidopsis MADS-domain protein AGAMOUS-like 2	SEP1	831436
Dof zinc finger protein DOF1.6 (AT1G47655)	AT1G47655, DOF1.6	841175
Calmodulin-binding transcription activator 1 (AtSR2)	EICBP.B	830800
GATA transcription factor 19 (HANABA TARANU LIKE 2, HANL2)	GATA19	829814
Myc recognition sequences	NDL3	816481
Arabidopsis leucine zipper protein TGA1	TGA1	836646
Dehydration-responsive element-binding protein A-4	DREB2A	830424
Sunflower homeodomain leucine-zipper protein Hahb-4	HB4	819100

E2F class I sites	E2F1 E2F	832283
Trihelix-domain transcription factor VFP5 (AT5G05550)	AT5G05550	830438
Squamosa promoter-binding-like protein 5	SPL5	820758
Agamous, required for normal flower development,	AP1	843244
Trihelix transcription factor GTL2	At5g28300	832914
Phytochrome interacting factor 4	PIF4	818903
BBES1/BZR1-like protein 3 (AT4G18890)	BEH3	827623
Mycorrhiza transcription factor	SHR	829919
Squamosa promoter-binding-like protein 1	SPL1	819321
WRKY DNA-binding protein 45	WRKY45	821270
Homeobox protein 33	HB33	843861
Basic region / leucine zipper motif 60	BZIP60	840897
Heat stress transcription factor A-6b	HSFA6b	821854
Callose Defective Microspore 1 (zinc finger CCCH domain-containing protein 15)	AT2G24830	817020
Myb family transcription factor REVEILLE 1	RVE1	831595
Basic leucine-zipper 52	bZIP52	837197
Oryza sativa bZIP protein 8	bZIP1	835005
Maize activator P of flavonoid biosynthetic genes	P1	542272
Late Meristem Identity 1 (AtHB51, LMI1)	HB51	831723
Reproductive Meristem 1	DCL1	839574
ABORTED MICROSPORES	MYB12	819359
High mobility group I/Y-like protein isolated from pea	HMGB1	824351
Myb domain protein 121	MYB121	822714
Ethylene-responsive transcription factor RAP2-6	RAP2.6	840915
M-phase-specific activators (NtmybA1, NtmybA2, NtmybB)	MYB3R-4	831023
Myb family transcription factor AT1G74840	AT1G74840	843823
HDZip class I protein ATHB5	HB5	836656
Member of the EPF family of zinc finger transcription factors	SUP	821888
ERSE I (ER stress-response element I)-like motif	BZIP60	840897
SHI related sequence 1 (STYLISH 1)	STY1	824270
FAR-RED ELONGATED HYPOCOTYLS 3 and FAR-RED IMPAIRED RESPONSE 1	FHY3<br>HFR1	821781<br>839300
Myb domain protein 67 (ATY53)	MYB67	820454
Myb domain protein R1 (ATMYB44)	MYBR1	836865
C-repeat/dehydration response element	DREB1A	828652
Two-component response regulator ARR14	RR14	814707

Asymmetric leaves2-like 18 (LOB domain-containing protein 16)	LBD16	818843
Plant specific floral meristem identity gene LEAFY (LFY)	LFY	836307
Hordeum vulgare Myb-related CAB-promoter-binding protein 1	CAB1	839871
HD-ZIP class III protein ATHB9	PHV	839928
Myb-domain transcription factor werewolf	MYB66	831327
Myb domain protein 31 (AtMYB31)	MYB31	843804
AGL1, Arabidopsis MADS-domain protein AGAMOUS-like 1	SHP1	825047
VirE2-interacting protein 1	VIP1	840957
NAC domain containing protein 38	NAC038	816979
Homeobox-leucine zipper protein ATHB-53	HB53	836803
Heat shock transcription factor C1	HSFC1	822047
WRKY DNA-binding protein 20	WRKY20	828771
Myb domain protein 33	MYB33	830497
Transcription factor NAC2	NAC2	830320
Brassinosteroid insensitive 1-EMS suppressor 1	BES1	838518
Myb domain protein 59	MYB59	836099
ABA (abscisic acid) inducible transcriptional activator	AIB	819262
Myb domain protein 93	MYB93	840371
Myb family transcription factor At5g56840	At5g56840	835786
High Cambial Activity 2 (AT5G62940, DOF5.6)	HCA2	836414
NAC domain containing protein 58	NAC058	821369
Oriza sativa leaf and tiller angle increased controller	MAC5B	817507
bZIP factors DPBF-1 and 2 (Dc3 promoter binding factor-1 and 2)	ABI5	818199
MADS-box protein GORDITA (AGL63)	GOA	839999
Myb domain protein 88	MYB88	814812
Target of early activation tagged 1 (RAP2.7)	RAP2.7	817403
TCP class I transcription factor (Arabidopsis)	TCP4	820732
TCP class II transcription factor	BRC1	821386
Early-phytochrome-responsive 1 (REVEILLE 7)	RVE8	820117
GATA transcription factor 4	GATA4	825224
Arabidopsis thaliana class A heat shock factor 1a	HSF1	827496
ULT1 interacting factor 1 (AT4G37180)	AT4G37180	829872
Mitochondrial transcription termination factor family protein (mTERF	BSM	828116
Anaerobic basic leucine zipper	abz1	544180
H. vulgare dehydration-response factor 1	DREB1A	828652
ABA insensitive protein 4 (ABI4)	ABI4	818614

Iron-deficiency-responsive element 1 (IDEF1)	FRU	817362
DREB and EAR motif protein 3	DEAR3	816866
Flowering bHLH 3	MYC3	834719
Tomato MADS-box transcription factor MADS-RIN	MYC2	840158
Tobacco bHLH transcription factor MYC2	MYC2	840158
GATA transcription factor 11	GATA11	837316
Elicitor response element	WRKY33	818429
Myb domain protein 101 (ATM1)	MYB101	817807
Dwarf and Delayed Flowering 1	ddf2	842606
Phytochrome B-regulated transcription factor PIF3 (nuclear-localized bHLH-protein)	PIF3	837479
DIVARICATA 6 (myb/SANT-like DNA-binding domain-containing protein AT5G05790)	AT5G05790	830464
Myb domain protein 3R-5	MYB3R-5	831802
FAR1-related sequence 9	FRS3	817253
LOB domain-containing protein 13	LBD13	817584
NAC domain containing protein 92 (NAC6)	NAC6	833957
Myb-like HTH transcriptional regulator-like protein (G2-like family protein)	APRR2	827527
Myb/SANT-like DNA-binding domain-containing protein MYBS1	AT5G08520	830751
Short Vegetative Phase	SVP	816787
Myb family transcription factor AT1G19000	AT1G19000	838481
WRKY transcription factor 7	WRKY7	828525
bZIP transcription factor Elongated Hypocotyl 5	HY5	830996
Floral homeotic protein APETALA1	AP1	843244
DOF Affecting Germination 2	DAG2	819271
Auxin Response Element	IAA7	821879
Myb related transcriptional activator, direct ortholog of S.pombe CDC5 protein	CDC5	837506
Zinc finger CCCH domain-containing protein 67 (AT5G63260)	AT5G63260	836446
TCP domain protein 16	TCP16	823651
Myb domain protein 61	MYB61	837480
myb domain protein 58	MYB58	838219
Myb domain protein 27	MYB27	824486
Myb family transcription factor At5g61620	AT5G61620	836284
Trihelix transcription factor GT-2	GTL1	840218
Homeobox-leucine zipper protein HAT2	HAT2	834784
Rice iron-related transcription factor 2	LOC4325750	4325750
Transcription factor DYSFUNCTIONAL TAPETUM 1	DYT1	827883
Dof2 - single zinc finger transcription factor	DOF2	821681

Myb hypocotyl elongation-related (AT5G47390, KUODA	AT5G47390	834786
Suppressor of overexpression of CO 1 (AGL20)	AGL20	819174
Heat stress transcription factor B-2a (HSF6)	**HSFB2A**	836322
Squamosa promoter-binding-like protein 13	SPL13A	835126
Ethylene-responsive transcription factor ERF057 (AT5G65130) Ethylene and salt inducible 3	AT5G65130	836637
Dehydration-responsive element-binding protein 2C	DREB2C	818627
B-box domain protein 31	BBX31	821744
Telomere repeat-binding protein 1	TRB2	836894
Transcription factor TGA4 (OBF4)	TGA4	830866
R2R3-MYB transcription factor AtMYB77	MYB77	824168
Brassinazole-resistant 1 protein	BZR1	843845
Arabidopsis NAC domain containing protein 92 (ATNAC2/ATNAC6)	LOC109243243	109243243
Transcription factor Big Petal (AT1G59640)	BPEp	842254
Myb domain protein 96	MYB96	836367
WRKY transcription factor 22	WRKY22	827896
DREB and EAR motif protein 4 (RAP2.10)	RAP2.10	829843
Tobacco bZip transcription activator (TAF-1)	HAF2	821437
Ethylene-responsive transcription factor ERF043 (AT4G32800)	AT4G32800	829416
Dof zinc finger protein DOF4.2	827850	827850
Homeobox-leucine zipper protein ATHB-20	HB20	821232
Transcription factor TGA2	AHBP-1B	830586
Ethylene-responsive transcription factor ERF017 (AT1G19210)	AT1G19210	838504
Ethylene-responsive transcription factor 1A (AT4G17500)	ERF-1	827464
Dehydration-responsive element-binding protein 2G (AT5G18450)	AT5G18450,	831963,
FAR-RED Impaired Response 1	AT3G59470	825116
Ethylene-responsive transcription factor 1B (AT3G23240)	ERF1	821902
Ethylene-responsive transcription factor RAP2-6	RAP2.6	840915
Dehydration response element-binding protein 19	DREB19	818414
Ethylene-responsive transcription factor ERF055 (AT1G36060)	AT1G36060	840510
NAC (No Apical Meristem) domain transcriptional regulator superfamily protein	NAC018	100779770
Myb domain protein 74	MYB74	825855
Dof zinc finger protein DOF5.4 (OBF binding protein 4)	820929	836206
NAC domain containing protein 17	NAC017	840318
Transcription factor TCP22 (AT1G72010)	AT1G72010	843532

Homeobox-leucine zipper protein ATHB-13	ATHB13	843314
Dof zinc finger protein DOF3.5 (AT3G52440)	AT3G52440	824409
HRS1 Homolog 3 (AT1G25550)	AT1G25550	839142
Abscisic acid insensitive 5 (bZIP12, EEL)	ABI5	818199
Myb domain protein 119	MYB119	836002
Homeobox-leucine zipper protein ATHB-21	820929	814784
Homeobox-leucine zipper protein ATHB-18	820929	843431
Silencing element binding factor - transcriptional repressor	AT2G42940	818895
Duplicated SANT DNA-binding domain-containing protein MYBS2	AT5G08520	830751
Ethylene-responsive transcription factor RAP2-3	EBP	820929
Dof zinc finger protein DOF4.5	DOF4.5	827855
Ethylene-responsive transcription factor ERF018	ERF018, ORA47	843832
Heat stress transcription factor A-4a (HSF21)	HSFA4A	827622
FYF Up-regulating 321 Factor 1 (AT1G71450	AT1G71450	843486
Ethylene-responsive transcription factor RAP2-11	RAP2.11	832099
Redox responsive transcription factor 1	RRTF1	829591
Ethylene-responsive transcription factor 8	ERF8	841751
Ethylene-responsive transcription factor RAP2-3	EBP	820929
Target of early activation tagged (EAT) 2	TOE2	836134
Ethylene-responsive transcription factor 15 (AT2G3123	ERF15	817680
Transcription factor TGA2	AHBP-1B	830586
Cooperatively regulated by ethylene and jasmonate 1 (DEAR1)	CEJ1	824188
CUP-SHAPED COTYLEDON 3	CUC3	843975
CUP-SHAPED COTYLEDON 2	CUC2	835478
NAM-like protein CUP-SHAPED COTYLEDON 1	CUC1	820748
BES1-interacting Myc-like protein 1	BIM3	833877
BES1-interacting Myc-like protein 2	BIM2	843233
KNOTTED1 (KN1) and KNOTTED interacting protein (KIP)	KNAT1	826364
NAC domain containing protein 2	NAC2	830320
WRKY DNA-binding protein 40	WRKY40	844423
WRKY DNA-binding protein 18	WRKY18	829308
WRKY DNA binding protein 12	WRKY12	819083
WRKY DNA binding protein 38	WRKY38	832320
Ethylene-responsive transcription factor ERF112 (AT2G33710)	AT2G33710	817936
HBP-1a, suggested to be involved in the cell cycle-dependent expression	LOC103654950	103654950
TCP domain protein 7	AT5G23280	832392
LOB domain-containing protein 18	LBD18	819150

DNA-binding protein S1FA3	AT3G09735	820131
bZIP transcription factor TGA1 (bZIP47)	TGA1	836646
VIRE2-interacting protein 1	VIP1	840957
Ethylene-responsive transcription factor ERF039 (AT4G16750)	AT4G16750	827379
ANT (Arabidopsis protein AINTEGUMENTA), member of the plant-specific family of AP2/EREBP-transcription factors	ANT	829931
Basic region/leucine zipper transcription factor 68	bZIP68	840107
Vascular related NAC-domain protein 1	VND1	816318
Basic leucine-zipper 28	LOC9318764	9318764
Myb domain protein 55	MYB55	826853
PHR1-like 1 (AT5G29000)	PHL1	833026
BES1/BZR1 homolog 4 (AT1G78700)	BEH4	844206
DIVARICATA 5 (VHA-B1-interacting transcription factor AT3G11280)	AT3G11280	820299
TCP domain protein 2 (Teosinte branched 1)	TCP2	827568
TCP domain protein 24	TCP24	839901
NAC domain containing protein 13	NAC13	840181
Ethylene-responsive transcription factor ERF014 (AT1G44830)	AT1G44830	841047
Squamosa promoter-binding-like protein 14	SPL14	838692
CCHC-type zinc knuckle protein	TZP	834383
Cytokinin Response Factor 10	CRF10	843184
Transcription factor TCP23	AT1G35560	840452
Ethylene-responsive transcription factor ERF019 (AT1G22810)	AT1G22810	838887
Ethylene-responsive transcription factor 2	ERF2	834768
Ethylene-responsive transcription factor 10	ERF10	839173
Rice bHLH protein	bHLH39	824865
Ethylene-responsive transcription factor 115	AT5G07310	830622
Dehydration-responsive element-binding protein 2C	DREB2C	818627
TCP domain protein 21 (AT5G08330)	TCP21	830730
Ethylene-responsive transcription factor 5	ERF5	834770
WRKY transcription factor 8	WRKY8	834678
C-repeat-binding factor 2 (DREB1B)	CBF1	828653
C-repeat-binding factor 3 (DREB1A)	DREB1A	828652
C-repeat-binding factor 2 (DREB1C)	CBF2	828651
E2F transcription factor 3	E2F3	818174
LIM domain protein binding to a PAL-box like sequence	WLIM1	837558
BBES1/BZR1-like protein 2 (AT4G36780	BEH2	829831
Transcription factor TCP15 (AT1G69690)	TCP15	843305

Heat stress transcription factor A-1b (HSF3)	HSF3	831545
NAC domain containing protein 3 (ORE1 SISTER1)	NAC3	820789
Heat stress transcription factor C-1	HSFC1	822047
Myb domain protein 116 RY and Sph motifs conserved in seed-specific promoters	MYB116	839118
NAC domain containing protein 45	NAC045	821226
Transcription factor TGA3	TGA3	838812
Arabidopsis NAC domain containing protein 81	LOC106396474	106396474
ATAF2 WRKY transcription factor 55	WRKY55	818669
Indeterminate(ID)-domain 11 (AtIDD11)	IDD11	820593
Dof zinc finger protein DOF4.7 (AT4G38000)	DOF4.7	829956
Indeterminate(ID)-domain 5 protein (RAVEN)	IDD5	814738
ABA-responsive element binding protein 3	AREB3	824852
Phytochrome-interacting fator 5 (PIL6)	PIL6	825075
Dof zinc finger protein DOF5.1 (AT5G02460)	AT5G02460	830976
Dof zinc finger protein DOF2.2 (AT2G28810)	OBP2	837277
Myb domain protein 118 (Plant Growth Activator 37, PGA37)	MYB118	822399
B3 domain-containing transcription factor NGA4	NGA4	826750
Myb domain protein 111	MYB111	834993
Dof zinc finger protein DOF5.8 (AT5G66940)	AT5G66940	836828
NAC domain containing protein 96	NAC096	834702
Ethylene-responsive transcription factor 105	AT5G51190	835193
REVEILLE 5	AT4G01280	827903

Table S3B

**Responsive element**	**Activating factor**
Iron-dependent regulatory sequence	iron
Sugar responsive elements	sugar
Ethylene-responsive elements (ERE) and jasmonate- and elicitor-responsive elements (JERE)	ethylene<br>jasmonate
CA-rich element	microRNA
Auxin Response Element	auxin

Promoter elements involved in MgProto (Mg-protoporphyrin IX) and light-mediated induction	Mg<br>Light
Sequence motif from the promoters of different sugar-responsive genes	sugar
Coupling element 3 (CE3), non-ACGT ABRE	ABA
Sugar and ABA responsive element conserved in several rbcS promoters	sugar, ABA
ABA response elements	ABA
CAACTC regulatory elements, GA-inducible	GA
I-Box in rbcS genes and other light regulated genes	LIGHT
Prolamin box, conserved in cereal seed storage protein gene promoters	prolamin
Hormone up-regulated at dawn element	hormones
GA-regulated myb gene from barley	gibberellins
Heat shock element	heat
Motif similar to the conserved 8 bp distal element of the 43 bp pseudo-palindromic nitrogen response element (NRE)	nitrogen
Jasmonate response element JRE	jasmonate
TEF cis acting elements in both RNA polymerase II-dependent promoters and rDNA spacer sequences	
Ribosomal protein box, appears unique to plant RP genes and genes associated with gene expression	
Plant TATA box	
Avian C-type LTR TATA box	
Cellular and viral TATA box elements	
Mammalian C-type LTR TATA box	
